# (Salen)Mn(iii)-catalyzed chemoselective acylazidation of olefins†
†Electronic supplementary information (ESI) available. CCDC 1546115. For ESI and crystallographic data in CIF or other electronic format see DOI: 10.1039/c8sc01882k


**DOI:** 10.1039/c8sc01882k

**Published:** 2018-06-19

**Authors:** Liang Zhang, Shuya Liu, Zhiguo Zhao, Hongmei Su, Jingcheng Hao, Yao Wang

**Affiliations:** a School of Chemistry and Chemical Engineering , Key Laboratory of the Colloid and Interface Chemistry , Shandong University , 27 Shanda Nanlu , Jinan 250100 , Shandong , China . Email: yaowang@sdu.edu.cn

## Abstract

We describe a (salen)Mn(iii)-catalyzed three-component reaction of aldehydes, olefins, and sodium azide for the installation of two useful groups (C=O and N_3_) into the double bond. Traditionally, (salen)Mn(iii) in conjunction with iodosobenzene is a classical catalysis system for epoxidation of olefins. Owing to the highly competitive oxygenation approaches, it is a true challenge to establish a distinct strategy for the exploration of new olefin transformations based on this (salen)Mn(iii) catalysis system. Herein, the key to this (salen)Mn(iii)-catalyzed acylazidation of olefins was the rational application of the distinct reactivity of oxomanganese(v) species which is capable of abstracting a hydrogen atom from a substrate C–H bond. This chemoselective reaction occurred in a precisely designed reaction sequence and tolerates complex molecular structures.

## Abstract 2

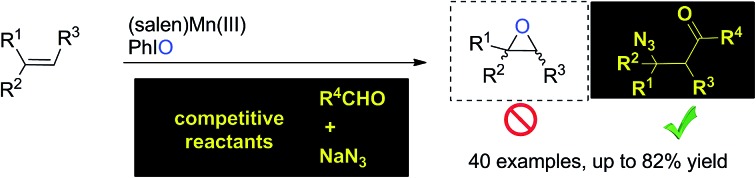
A (salen)Mn(iii)-catalyzed chemoselective acylazidation of olefins was developed based on strategic application of the distinct reactivity of oxomanganese(v) species.

## Introduction

**Fig. 1:**
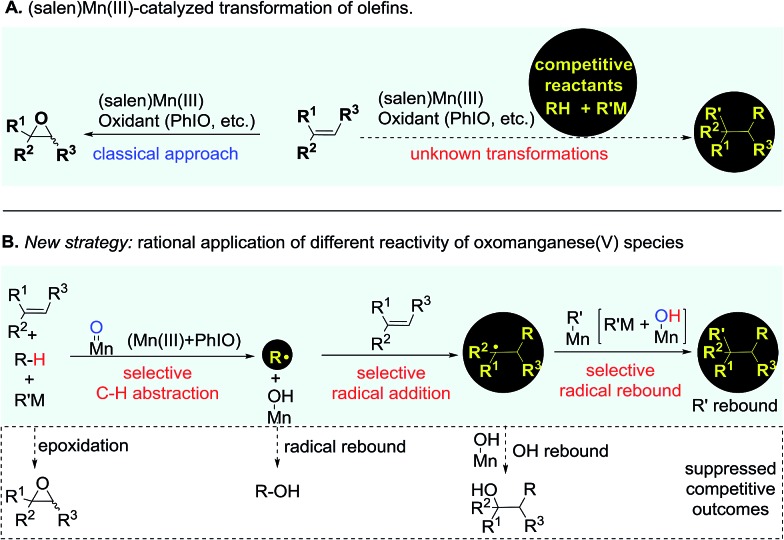
(Salen)Mn(iii)-catalyzed competitive transformation of olefins.

Catalytic transformation of olefins is one of the fundamental approaches for the generation of molecular complexity and diversity. A range of elegant and powerful strategies have been established for the installation of a diverse array of useful functional groups into the double bonds, which has essentially advanced the art and practical use of organic synthesis.^1^ Amongst various catalysts for olefin transformations, manganese Schiff-base complexes have been well-established as powerful catalysts for epoxidation of olefins in the last three decades.^2^–^5^ It has been a general notion that the oxomanganese(v) species *in situ* generated through oxidation of the (salen)Mn(iii) complex is an efficient oxygen-transfer species for epoxidation of various olefins.^6^,^7^ Owing to the fast and strong oxygenative background reactions,^5^–^7^ it remains a substantial challenge to develop a distinct strategy from a fresh perspective that enables the discovery of previously unknown olefin transformations based on this oxidative (salen)Mn(iii) catalysis system (Fig. 1A).

The reactive oxomanganese(v) species can readily abstract a hydrogen atom even from inert C–H bonds such as those in alkanes to generate a substrate-derived radical and a hydroxomanganese(iv) intermediate under mild reaction conditions.^8^,^9^ This distinct reactivity of oxomanganese(v) species provides a basis and opens up new opportunities for the development of a useful strategy from a fresh perspective enabling the transformation of olefins. As outlined in Fig. 1B, we envisioned that the addition of a carefully selected reactant bearing a weak C–H bond to the oxidative (salen)Mn(iii) catalysis system would lead to a preferential C–H abstraction, thus shutting down the highly competitive epoxidation pathway. Considering the fast radical rebound pathway,^10^,^11^ it is essential that the *in situ* generated radical is capable of immediately reacting with the ‘spectator’ olefin to start the precisely designed reaction sequence towards the desirable outcome. Finally, a selective radical rebound reaction would finish the whole reaction sequence. A standing challenge for implementing the designed strategy has been the suppression of these consecutively competitive approaches.^12^,^13^

In contrast to alkanes, aldehydes have a weaker (O=)C–H bond which can be feasibly cleaved to generate acyl radicals.^14^,^15^ Hydroacylation of olefins using aldehydes as an acyl source represents a simple and efficient method for the preparation of ketones.^15^–^18^ Under substantially different reaction pathways, aldehydes have been applied in acylation of highly electron-deficient olefins^19^–^22^ or unactivated olefins.^23^–^28^ Despite the important achievements in radical acylation of olefins, there are considerable limitations that remain to be resolved with regard to the scope of aldehydes and olefins.^19^–^22^ At room temperature, acyl radicals are generally reactive towards the addition of highly electron-deficient olefins while they show very poor or no reactivity towards less electron-deficient olefins. Aldehydes are generally restricted to a specific class to avoid the decarbonylation problem and to enable the desirable acyl radical addition of olefins. Furthermore, for an intermolecular hydroacylation approach, the installation of a functional group instead of a hydrogen atom through trapping of the acylation intermediate remains a largely elusive problem. We envisioned that the rationally designed strategy could provide a promising solution to these synthetic problems. Herein, we present our findings on the establishment and application of this strategy in the development of a chemoselective acylazidation of olefins.

## Results and discussion

Extensive investigation of numerous possible combinations of competitive reactants was carried out. Encouragingly, we were able to identify that the addition of a combination of aldehyde and sodium azide to the (salen)Mn(iii) catalysis system could suppress oxygenation approaches, resulting in the chemoselective formation of an acylazidation product. Organic azides can participate in a range of important reactions including Staudinger ligation,^29^ 1,3-dipolar cycloaddition,^30^ and the aza-Wittig reaction,^31^ making them highly attractive targets for organic synthesis.^32^ A range of elegant methods have been developed for azidation of olefins^33^ and C–H azidation.^34^ To optimize the reaction conditions, styrene was used as a model substrate while *n*-butylaldehyde and sodium azide were employed as a combination of competitors. As shown in Table 1, initially, several control experiments were conducted. No product was observed in the absence of either the (salen)Mn(iii) catalyst or PhIO (entries 1–2). In the absence of *n*-butylaldehyde and sodium azide, as previously reported by Kochi and co-workers,^12^ a fast consumption of styrene was observed (<1 h, >95% conversion of styrene, 30% epoxide, entry 3). The acylazidation product was formed in 57% yield catalyzed by **C1** in CH_3_CN (entry 4). Solvent optimization showed that a mixed solvent of EtOAc and H_2_O is an optimal choice, which afforded the desired product in 75% yield (entries 5–9). The evaluation of different (salen)Mn(iii) complexes revealed that catalyst **C1** is a promising candidate (entries 10–14). Upon using 1 mol% of catalyst **C1**, the reaction yield was decreased (entry 15). Further optimization of the amount of aldehyde and iodosobenzene did not improve the reaction yield (entries 16–18). The optimized reaction conditions did not provide an observable quantity of the epoxidation product. In contrast, 41% epoxide was obtained in the absence of *n*-butylaldehyde and sodium azide (entry 19).

**Table 1:** Optimization of reaction conditions^*a*^

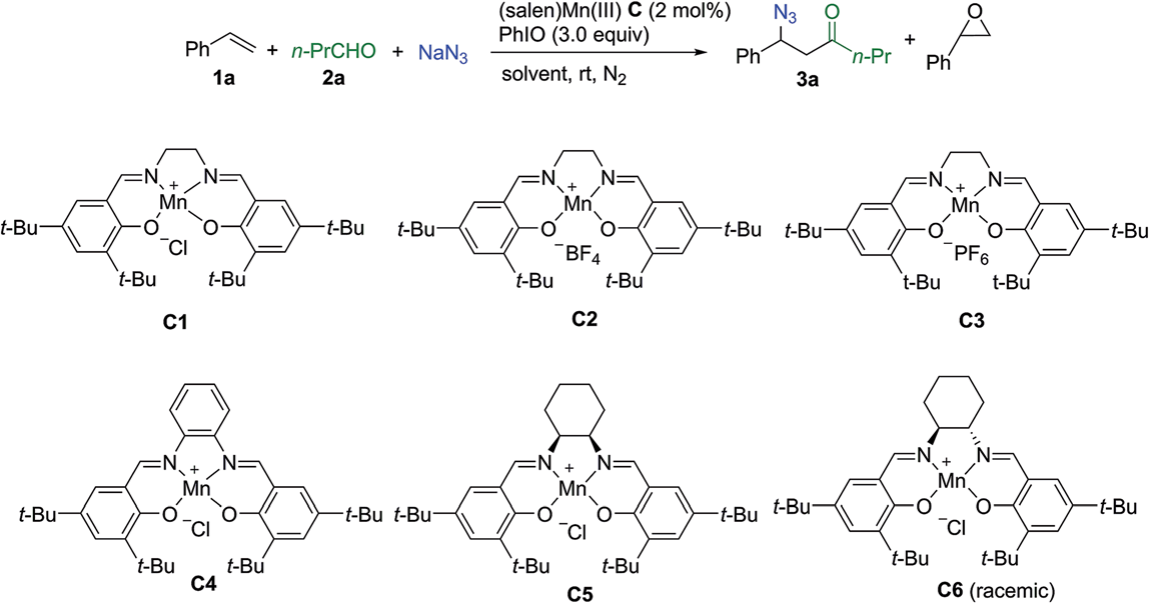					
Entry	Catalyst	Solvent	Time (h)	Epoxide^*b*^ (%)	Yield^*b*^ (**3a**%)
1	—	CH_3_CN	12	n.r.	n.r.
2^*c*^	**C1**	CH_3_CN	12	n.r.	n.r.
3^*d*^	**C1**	CH_3_CN	<1	30	—
4	**C1**	CH_3_CN	6	<5	57
5	**C1**	EtOAc	6	6	45
6	**C1**	DCM	6	<5	27
7	**C1**	Toluene	6	<5	39
8^*e*^	**C1**	H_2_O/CH_3_CN	3	<5	52
9	**C1**	H_2_O/EtOAc	3	<5	75
10	**C2**	H_2_O/EtOAc	3	<5	65
11	**C3**	H_2_O/EtOAc	3	<5	68
12	**C4**	H_2_O/EtOAc	5	7	11
13	**C5**	H_2_O/EtOAc	9	<5	65
14	**C6**	H_2_O/EtOAc	5	<5	55
15^*f*^	**C1**	H_2_O/EtOAc	6	<5	58
16^*g*^	**C1**	H_2_O/EtOAc	6	<5	54
17^*h*^	**C1**	H_2_O/EtOAc	6	<5	49
18^*i*^	**C1**	H_2_O/EtOAc	6	<5	51
19^*d*^	**C1**	H_2_O/EtOAc	4	41	—

^*a*^Unless otherwise noted, all the reactions were carried out with **1a** (0.3 mmol), **2a** (1.5 mmol), NaN_3_ (1.2 mmol), PhIO (0.9 mmol) and **C** (2 mol%) in 3.0 mL solvent as indicated at room temperature.

^*b*^Isolated yield.

^*c*^Without PhIO.

^*d*^Without **2a** and NaN_3_.

^*e*^6.4 mL mixed solvent was used (H_2_O/organic solvent = 1/0.6, entries 8–19).

^*f*^1 mol% of **C** was used.

^*g*^0.9 mmol of **2a** was used.

^*h*^2.0 equiv. of PhIO were used.

^*i*^4.0 equiv. of PhIO were used. n.r. = no reaction.

**Scheme 1:**
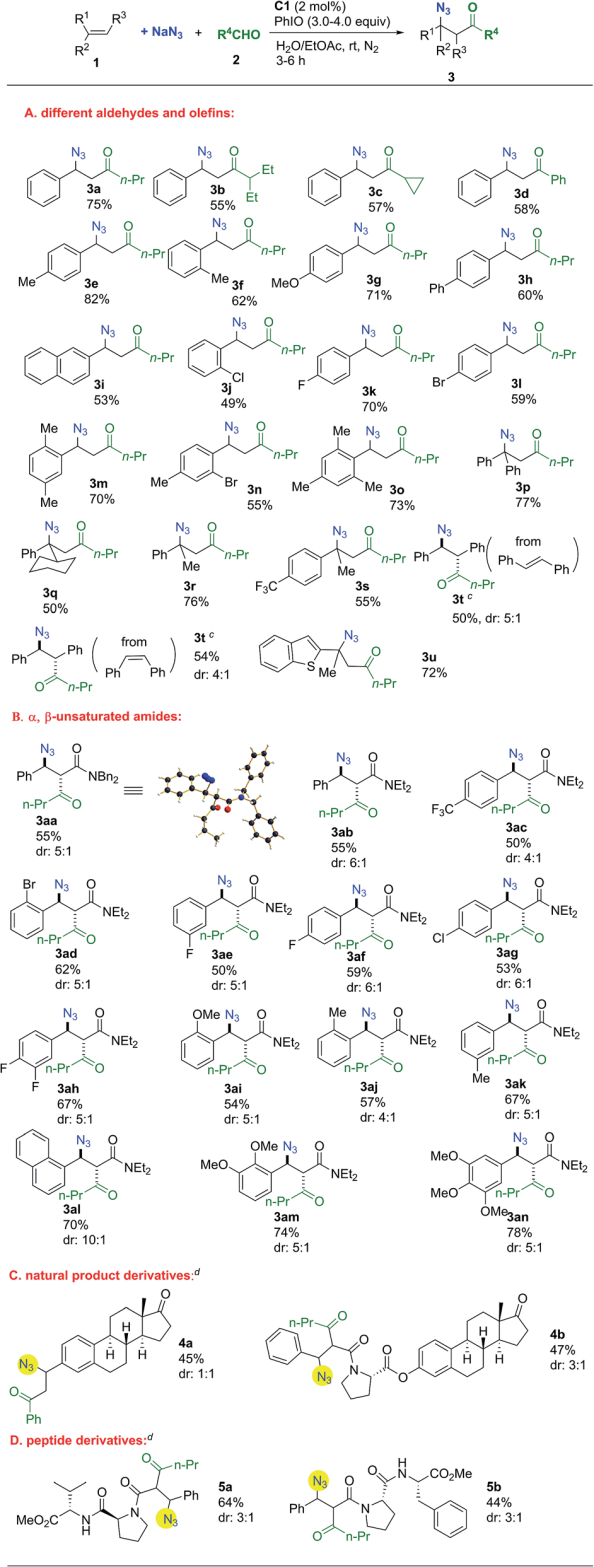
Substrate scope^a,b^. ^a^Unless otherwise noted, all the reactions were carried out with **1a** (0.3 mmol), **2a** (1.5 mmol), NaN_3_ (1.2 mmol) and **C** (2 mol%) in 6.4 mL H_2_O/EtOAc (1/0.6) at room temperature. ^b^The diastereoselectivity was determined by ^1^H NMR analysis of the crude reaction mixture and yields refer to the isolated major product. ^c^1 mol% of catalyst **C1** and 6.0 equiv. of PhIO were used (compound **3t**). ^d^The diastereoselectivities of **4b**, **5a** and **5b** refer to the ratio of the two major isomers.

The substrate scope was investigated next. As shown in Scheme 1, remarkably, all the representative aldehydes, including linear- and branched-aliphatic aldehydes and aromatic aldehydes, could be tolerated and the desired products were obtained in reasonable yields (**3a–d**). Aromatic rings of olefins bearing electron-donating or electron-withdrawing groups were tolerated. Meanwhile, substituent groups could be incorporated in diversified positions, delivering *ortho*-, *meta*-, and *para*-substituted products. Furthermore, multi-substituted aromatic rings were amenable to this transformation (**3m–o**). The employment of several α-substituted styrene derivatives for the synthesis of a range of tertiary azides was successful (**3p–s**). Both *trans*- and *cis*-stilbene could be used as substrates to afford the same product with similar diastereoselectivities in 50% and 54% yield, respectively (**3t**). Under standard conditions using *cis*-stilbene as a substrate, acylazidation product **3t** was obtained in 30% yield along with 17% epoxide. Interestingly, reducing the catalyst loading to 1 mol% was effective in bringing about a vigorous competition between the reactions of acylazidation and epoxidation of stilbene since the acylazidation approach overwhelmingly dominated the reaction pathways. A heterocyclic substrate could also be tolerated in this reaction (**3u**).

Next, the use of electron-deficient α,β-unsaturated amides as substrates was studied. All the α,β-unsaturated amides smoothly afforded the desired products with moderate to good diastereoselectivity in reasonable yields (Scheme 1). Aromatic groups with different electrical properties such as electron-withdrawing or electron-donating groups were tolerated, as were *ortho*-, *meta*-, and *para*- substituted aromatic rings. The structure of **3aa** was confirmed by single-crystal X-ray diffraction.^35^ This methodology could be applied to more complex contexts. Estrone derivatives could be applied in this transformation. It is of significance to incorporate the azido group into peptide derivatives for the synthesis of a wide range of complex molecules with promising biological activities.^36^,^37^ This methodology provides a concise pathway for the preparation of azidopeptide derivatives (Scheme 1).

**Fig. 2:**
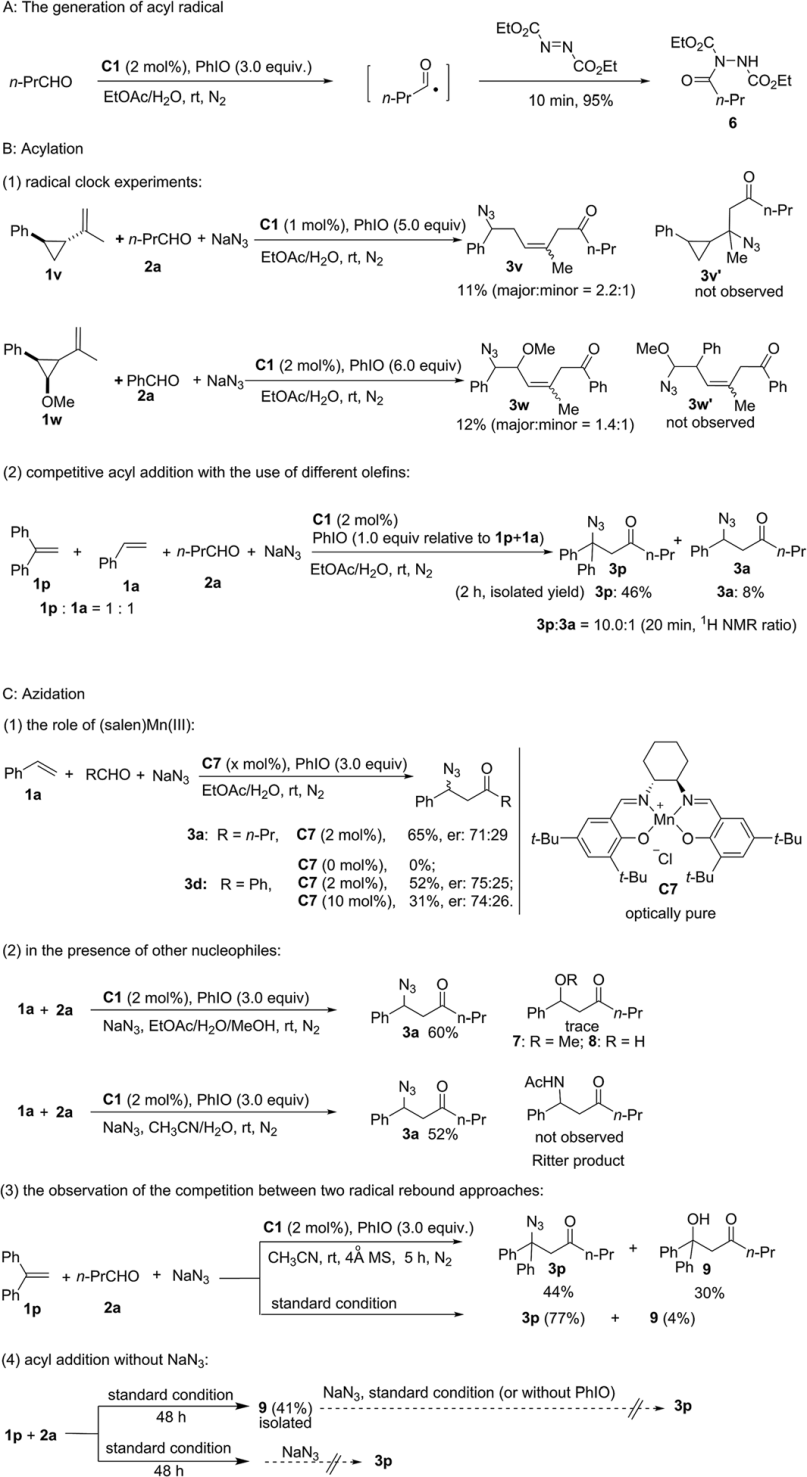
Probing mechanism.

As shown in Fig. 2, a series of control experiments were carried out to probe the mechanism (see the ESI†). Considering the significant work done by Jacobsen and co-workers who established that azide ring-opening of epoxides can be efficiently catalyzed by metal–salen complexes,^38^,^39^ the replacement of styrene with styrene oxide did not generate product **3a** either under standard reaction conditions or without PhIO. This experiment can exclude the possibility that **3a** was generated from complex transformation of styrene oxide. Furthermore, established work revealed that α,β-unsaturated ketones could be generated through copper-catalyzed oxidative coupling of alkenes with aldehydes.^40^ Meanwhile, Jacobsen and co-workers discovered that a Lewis acid was able to catalyze conjugate addition of azide to α,β-unsaturated ketones.^41^,^42^ However, no α,β-unsaturated ketone ((*E*)-1-phenylhex-1-en-3-one) was observed under standard reaction conditions using **1a** and **2a** as the starting materials. The reaction between a synthetic α,β-unsaturated ketone ((*E*)-1-phenylhex-1-en-3-one) and sodium azide catalyzed by (salen)Mn(iii) **C1** with (or without) PhIO failed to deliver the conjugate product **3a**, which excludes the possibility of a Lewis acid-catalyzed conjugate addition pathway.

The generation of an acyl radical in the presence of Mn(iii) and PhIO was observed by using diethyl azodicarboxylate as a trapping agent.^43^ Furthermore, using radical clock substrate **1v** afforded the ring-opening product **3v**. Newcomb-type cyclopropane substrate **1w** was employed to differentiate between a radical approach and a cationic pathway.^44^,^45^ Acyl addition to the olefin **1w** occurs with subsequent cleavage of the benzylic cyclopropyl bond rather than the α-methoxy cyclopropyl bond. Furthermore, an intermolecular competitive reaction between substrates **1p** and **1a** was carried out. Despite greater steric hindrance, compound **3p** was obtained as a major product and a ratio of 10 : 1 (20 min, **3p** : **3a**) was observed upon using a 1 : 1 mixture of **1p** and **1a**. This observation can be attributed to the competitive generation of a more stable dibenzylic radical. All these results clearly point to an acyl radical addition pathway.

To probe the azidation process, several control experiments were carried out. Nearly the same moderate enantioselectivity of **3d** was obtained in the presence of 2 mol% and 10 mol% of Jacobsen's catalyst **C7** while no product was observed without this catalyst. However, an intramolecular reaction using 2-(allyloxy)benzaldehyde as the substrate could proceed in the absence of a catalyst to afford chroman derivatives.^46^ Considering the potential pathway in which the oxidation of radical intermediate by manganese would lead to the formation of a carbocation, 20 equiv. of methanol was added to the standard reaction system to competitively trap the carbocation. However, neither methoxylated product **7** nor hydroxylated product **8** was observed. Furthermore, no product of the competitive Ritter reaction was detected upon using CH_3_CN/H_2_O as the solvent. Furthermore, competition between the two radical rebound approaches was observed and a considerable amount of hydroxylated product **9** (30%) was isolated in dry CH_3_CN. This experiment suggests that the formation of an acyl radical and a hydroxomanganese(iv) intermediate originated from (O=)C–H abstraction by the reactive oxomanganese(v) species. These experiments indicate an azido-rebound pathway. Only 4% of **9** was isolated, which indicates that the rate of azido-rebound is much faster than the rate of hydroxy-rebound under standard reaction conditions. Furthermore, in the absence of NaN_3_, product **9** was isolated in 41% yield, which clearly revealed that both the generation of the acyl radical and the subsequent radical addition of the olefin can occur in the absence of NaN_3_. Further control experiments revealed that the acylazidation product **3p** is not generated from the transformation of hydroxylated compound **9**.

**Fig. 3:**
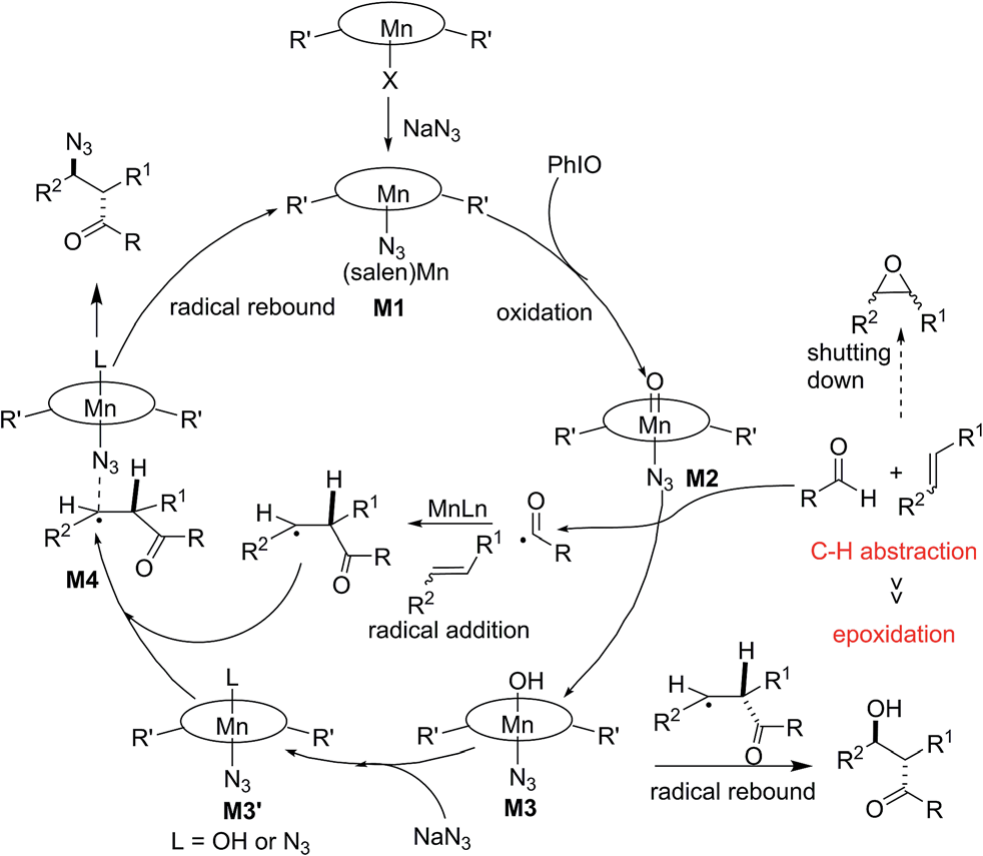
Plausible mechanism.

Based on these obtained results, a possible mechanism was proposed and is depicted in Fig. 3. Initially, a ligand exchange process resulted in a manganese-bound azide, **M1**. The remaining (salen)Mn(iii) is oxidized by PhIO, generating a reactive oxomanganese(v) intermediate, **M2**. The oxomanganese(v) species is capable of selectively abstracting a hydrogen from the aldehyde to form intermediate **M3** and an acyl radical. The subsequent acyl radical addition of a manganese-activated alkene would generate a pending radical intermediate. Finally, azido-rebound *via* complex **M4** releases the final product and (salen)Mn(iii) catalyst.

## Conclusions

In summary, enabled by the rational application of the distinct reactivity of oxomanganese(v) species, a new strategy was established for the transformation of olefins. A unique finding was that (salen)Mn(iii) in conjunction with iodosobenzene can induce an intermolecular acylazidation of olefins using aldehydes and sodium azide as competitive reactants. Both carbonyl and azido groups were incorporated into simple olefinic compounds as well as complex targets. This transformation enables electron-deficient, neutral and even electron-rich alkenes to participate in an acyl radical addition process at room temperature. The representative aldehydes, including linear- and branched-aliphatic aldehydes and aromatic aldehydes, could be used as effective reactants. Potentially, the strategy described herein could open the doors to new catalytic reactivity that has been unexplored.

## Conflicts of interest

There are no conflicts to declare.

## Supplementary Material

Supplementary information

Crystal structure data
