# Supplementary material for: (Salen)Mn(iii)-catalyzed chemoselective acylazidation of olefins
Source: Chem Sci. 2018 Jun 19;9(28):6085–90. doi: 10.1039/c8sc01882k (PMC6053955; doi:10.1039/c8sc01882k)
Supplement: Supplementary file 1 [file SC-009-C8SC01882K-s001.pdf]

Electronic Supplementary Material (ESI) for Chemical Science.
This journal is © The Royal Society of Chemistry 2018

Supporting Information

(Salen)Mn(III)-Catalyzed Chemoselective Acylazidation of Olefins

Liang Zhang, Shuya Liu, Zhiguo Zhao, Hongmei Su, Jingcheng Hao, and Yao Wang*

School of Chemistry and Chemical Engineering, Key Laboratory of the Colloid and Interface Chemistry, Ministry of Education, Shandong University, Jinan 250100, China

Email: yaowang@sdu.edu.cn

Table of Contents:

1. General information ....…………………………………………………………………S2
2. Preparation of substrates and catalysts……………………………………………......S2-S3
3. General procedure for acylazidation of olefinic compounds ……………………………….S3
4. Analytical data ……………………………………………………………………...S4-S12
5. Mechanistic study……………………………………………………...………..S12-S15
6. X-ray crystallographic data………………………………………………………….....S16
7. Copies of NMR spectra……………………………………………………………….S16-S62
8. HPLC trace of **3a** and **3d**…………………………………………………………..S63-S64
9. References……………………………………………………………………………….S65

1. General Information

All the chemicals were either purchased from commercial suppliers or purified by standard procedures as specified in *Purification of Laboratory Chemicals*, 4th Ed (Armarego, W. L. F.; Perrin, D. D. Butterworth Heinemann: 1997). All reactions were carried out under nitrogen atmosphere. Optically pure catalyst **C7** was purchased from Sigma-Aldrich: product number 404446, CAS Number: 138124-32-0. Analytical thin-layer chromatography (TLC) was performed on silica gel plates and analyzed by UV light or by potassium permanganate stains followed by heating. Flash chromatography was carried out utilizing silica gel (200-300 mesh). ^{1}H NMR, ^{13}C NMR spectra were recorded in $CDCl_3$ at room temperature on a Bruker AM-400 spectrometer (400MHz ^{1}H, 100MHz ^{13}C). The chemical shifts are reported in ppm relative to either the residual solvent peak (^{13}C) (δ = 77 ppm) or TMS (^{1}H) (δ = 0 ppm) as an internal standard. Data for ^{1}H NMR are reported as follows: chemical shift (δ ppm), multiplicity (s = singlet, d = doublet, t = triplet, m = multiplet, dd = doublet doublet), coupling constant (Hz), integration. Data for ^{13}C NMR are reported as chemical shift. HRMS were performed on a Bruker Apex II mass instrument (ESI).

2. Preparation of the substrates and catalysts

The substrates **1q**, **1s** were prepared according to the literature procedure as reported by Liwosz.[1] The substrates **1aa**–**1an** were prepared according to the literature procedure.[2-4] The substrate vinylestrone **4a'** was prepared according to the literature procedure.[5-6] The substrate **1u** was prepared according to the literature procedure.[7] (salen)Mn(III) complexes were prepared according to the literature procedure.[8-9]

The substrates **4b'**, **5a'**, and **5b'** were prepared according to the similar methods as reported by literature procedure.[10-11]

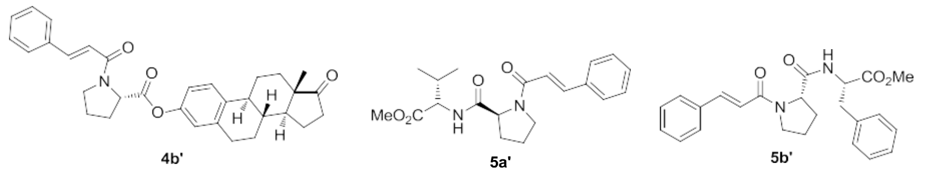

4b' **5a'** **5b'**

The preparation of **4b'**:

N-(3-dimethylaminopropyl)-*N*'-ethylcarbodiimide hydrochloride (920.0 mg, 4.8 mmol, 1.20 equiv) was added to a suspension of *(S)*-1-cinnamoylpyrrolidine-2-carboxylic acid (981.0 mg, 4.0 mmol, 1.0 equiv), estrone (1081.0 mg, 4.0 mmol, 1.0 equiv), and 4-dimethylaminopyridine (147.0 mg, 1.2 mmol, 0.3 equiv) in dry CH_2Cl_2 (40.0 mL), and then the reaction mixture was stirred at 23 °C for 15 h . The reaction mixture was then concentrated in vacuum and the residue was purified by chromatography on silica gel, eluting with CH_2Cl_2:CH_3OH (400:1 to 100:1 (v/v)), to afford **4b'** as a white solid (1295.0 mg, 2.6 mmol, 65% yield). ^{1}H NMR (400 MHz, $CDCl_3$): δ 7.77 (d, *J* = 15.5 Hz, 1H), 7.50-7.61 (m, 2H), 7.34-7.43 (m, 3H), 7.24-7.30 (m, 1H), 6.85-6.99 (m, 2H), 6.78 (d, *J* = 15.5 Hz, 1H), 4.81 (dd, *J* = 3.9, 8.4 Hz, 1H), 3.87-3.96 (m, 1H), 3.74-3.82 (m, 1H), 2.85-3.03 (m, 2H), 2.46-2.56 (m, 1H), 2.34-2.49 (m, 2H), 2.18–2.33 (m, 3H), 1.93–2.61 (m, 5H), 1.38–1.66 (m, 6H), 0.91 (s, 3H); ^{13}C NMR (100 MHz, $CDCl_3$): δ 220.7, 171.1, 165.0, 148.6, 142.9, 137.9, 137.3, 135.1, 129.8, 128.8, 127.9, 126.2, 121.5, 118.6, 117.9, 59.3, 50.4, 47.9, 47.0, 44.1, 38.0, 35.8, 31.5, 29.3, 29.2, 26.3, 25.7, 25.0, 21.5, 13.8; HRMS (ESI+) exact mass calculated for $[M+H]^+$ ($C_{32}H_{36}NO_4$) requires m/z 498.2644, found m/z 498.2645.

The preparation of the **5a'** and **5b'**:

To a stirring solution of *(S)*-1-cinnamoylpyrrolidine-2-carboxylic acid (1230.0 mg, 5.0 mmol) and triethylamine

(690.0 uL, 5.0 mmol) in THF (7.5 mL) was added isobutylchloroformate (630.0 uL, 5.0 mmol) at −10 ℃. After vigorous stirring for 50-60s, a solution of *L*-valine methyl ester hydrochloride (920.0 mg, 5.5 mmol) in DMSO (0.25 mL) was added followed by a solution of triethylamine (1.55 mL, 11.0 mmol) in THF (15.0 mL). The reaction mixture was then warmed to room temperature and vigorously stirred for 4.0 h. After the completion of the reaction, triethylamine hydrochloride was filtered off. The filtrate was concentrated and the residue was dissolved in EtOAc (30.0 mL). The solution was washed with saturated aqueous $NaHCO_3$ (2×10.0 mL), and brine (1×10 .0 mL), then dried over Na_2SO_4, filtered, and evaporated under reduced pressure to afford the crude product, which was further purified by column chromatography CH_2Cl_2:CH_3OH (500:1 to 100:3 (v/v)) to yield **5a'** in 55% yield (1092.0 mg, 55%). ^{1}H NMR (400 MHz, $CDCl_3$): δ 7.80 (d, *J* = 8.0 Hz, 1H), 7.77 (d, *J* = 15.4 Hz, 1H), 7.51-7.58 (m, 2H), 7.35-7.41 (m, 3H), 6.77 (d, *J* = 15.4 Hz, 1H), 4.81 (d, *J* = 7.2 Hz, 1H), 4.44 (dd, *J* = 5.1, 8.3 Hz, 1H), 3.76-3.81 (m, 1H), 3.74 (s, 3H), 3.61-3.69 (m, 1H), 2.45-2.53 (m, 1H), 2.13–2.23 (m, 2H), 1.99–2.10 (m, 1H), 1.80–1.92 (m, 1H), 0.91 (d, *J* = 6.8 Hz, 6H); ^{13}C NMR (100 MHz, $CDCl_3$): δ 172.2, 171.1, 166.2, 143.3, 134.9, 130.0, 128.8, 128.0, 117.8, 59.8, 57.6, 52.0, 47.4, 30.9, 26.8, 25.1, 19.1, 17.7; HRMS (ESI+) exact mass calculated for $[M+H]^+$ ($C_{20}H_{27}N_2O_4$) requires m/z 359.1971, found m/z 359.1964.

To a stirring solution of (*S*)-1-cinnamoylpyrrolidine-2-carboxylic acid (615.3 mg, 2.5 mmol) and triethylamine (346.5 uL, 2.5 mmol) in THF (3.8 mL) was added isobutylchloroformate (315.5 uL, 2.5 mmol) at −10 °C. After vigorous stirring for 50-60s, a solution of *L*-Phenylalanine methyl ester (448.0 uL, 2.75mmol) in THF (0.5 mL) was added dropwise. The reaction mixture was then warmed to room temperature and vigorously stirred for 4.0 h. After the completion of the reaction, the mixture was concentrated and the residue was dissolved in EtOAc (30.0 mL). The solution was washed with water (2×10.0 mL), and brine (1×10.0 mL), then dried over Na_2SO_4, filtered, and evaporated under reduced pressure to afford the crude product, which was further purified by column chromatography CH_2Cl_2:CH_3OH (300:1 to 100:3 (v/v)) to yield **5b'** in 73% yield (741.8 mg, 73%).^{1}H NMR (400 MHz, $CDCl_3$): δ 7.83 (d, *J* = 7.9 Hz, 1H), 7.74 (d, *J* = 15.4 Hz, 1H), 7.53–7.61 (m, 2H), 7.37–7.45 (m, 3H), 7.09–7.19 (m, 5H), 6.67 (d, *J* = 15.4 Hz, 1H), 4.80-4.88 (m, 1H), 4.74 (d, *J* = 7.0 Hz, 1H), 3.73 (s, 3H), 3.46–3.59 (m, 2H), 3.15-3.24 (m, 1H), 2.92-3.00 (m, 1H), 2.39-2.47 (m, 1H), 1.90–2.01 (m, 2H), 1.71-1.80 (m, 1H); ^{13}C NMR (100 MHz, $CDCl_3$): δ 171.9, 170.7, 166.1, 143.3, 136.3, 135.0, 130.0, 129.3, 128.9 128.2, 127.9, 126.6, 117.8, 59.6, 53.3, 52.2, 47.2, 38.1, 26.5, 24.8; HRMS (ESI+) exact mass calculated for $[M+H]^+$ ($C_{24}H_{27}N_2O_4$) requires m/z 407.1965, found m/z 407.1970.

3. General procedure for acylazidation of olefinic compounds

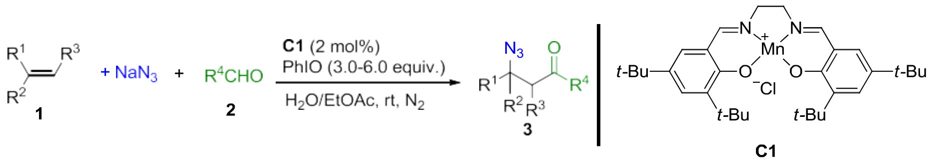

To a reaction mixture of catalyst **C1** (3.5 mg, 0.006 mmol, 2 mol%) and a fine powder PhIO (198.0 mg, 0.9 mmol, 3.0 equiv) in a 25 mL-Schlenk tube were added a aqueous solution of NaN_3 (4.0 mL, 0.3 M, 1.2 mmol, 4.0 equiv.), freshly distilled or purified aldehyde (1.5 mmol, 5.0 equiv.), and olefin **1** (0.3 mmol) under a nitrogen atmosphere. Then 2.4 mL EtOAc was added to the above reaction mixture and the reaction was stirred at room temperature. After the completion of the reaction as judged by TLC analysis (the reaction generally completed when the solid PhIO disappeared), the reaction mixture was extracted with EA (20.0 mL×1) and CH_2Cl_2 (15.0 mL×2). The combined organic layer was dried over anhydrous sodium sulfate and evaporated under reduced pressure to afford the crude product, which was purified by flash chromatography on silica gel to give the desired products. (Note: Grinding the solid PhIO into very fine powder and removing oxygen are

necessary for an efficient reaction).

4. Analytical data

1-azido-1-phenylhexan-3-one (3a):

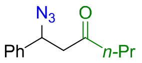

3a

Compound **3a** was synthesized according to the general procedure using 3.0 equiv PhIO (0.9 mmol, 198.1 mg) as a colorless oil in 75% yield; ^{1}H NMR (400 MHz, $CDCl_3$): δ 7.31–7.40 (m, 5H), 5.05 (dd, *J* = 4.8, 9.0 Hz, 1H), 2.95 (dd, *J* = 9.0, 16.9 Hz, 1H), 2.70 (dd, *J* = 4.8, 16.9 Hz, 1H), 2.31–2.46 (m, 2H), 1.55–1.64 (m, 2H), 0.89 (t, *J* = 7.4 Hz, 3H); ^{13}C NMR (100 MHz, $CDCl_3$): δ 207.4, 139.0, 128.9, 128.4, 126.7, 61.2, 48.8, 45.5, 16.9, 13.6; HRMS (ESI+) exact mass calculated for $[M+Na]^+$ ($C_{12}H_{15}N_3NaO$) requires m/z 240.1113, found m/z 240.1118.

1-azido-4-ethyl-1-phenylhexan-3-one (3b):

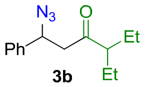

3b

Compound **3b** was synthesized according to the general procedure using 3.0 equiv PhIO (0.9 mmol, 198.3 mg) as a colorless oil in 55% yield; ^{1}H NMR (400 MHz, $CDCl_3$): δ 7.30–7.39 (m, 5H), 5.09 (dd, *J* = 4.8, 8.7 Hz, 1H), 2.98 (dd, *J* = 8.8, 17.4 Hz, 1H), 2.71 (dd, *J* = 4.8, 17.4 Hz, 1H), 2.26-2.33 (m, 1H), 1.54–1.67 (m, 2H), 1.37–1.51 (m, 2H), 0.84 (t, *J* = 7.4 Hz, 3H), 0.77 (t, *J* = 7.4 Hz, 3H); ^{13}C NMR (100 MHz, $CDCl_3$): δ 210.8, 139.2, 128.8, 128.4, 126.8, 61.1, 55.9, 48.4, 23.8, 23.7, 11.6, 11.6; HRMS (ESI+) exact mass calculated for $[M+Na]^+$ ($C_{14}H_{19}N_3NaO$) requires m/z 268.1426, found m/z 268.1423.

3-azido-1-cyclopropyl-3-phenylpropan-1-one (3c):

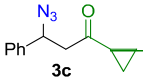

3c

Compound **3c** was synthesized according to the general procedure using 3.0 equiv PhIO (0.9 mmol, 198.6 mg) as a colorless oil in 57% yield; ^{1}H NMR (400 MHz, $CDCl_3$): δ 7.30–7.40 (m, 5H), 5.05 (dd, *J* = 4.7, 8.9 Hz, 1H), 3.11 (dd, *J* = 8.9, 17.0 Hz, 1H), 2.89 (dd, *J* = 4.7, 17.0 Hz, 1H), 1.87–1.93 (m, 1H), 1.01– 1.11 (m, 2H), 0.84–0.94 (m, 2H); ^{13}C NMR (100 MHz, $CDCl_3$): δ 207.10, 139.0, 128.8, 128.3, 126.7, 61.2, 49.4, 21.1, 11.2, 11.1; HRMS (ESI+) exact mass calculated for $[M+Na]^+$ ($C_{12}H_{13}N_3NaO$) requires m/z 238.0956, found m/z 238.0961.

3-azido-1,3-diphenylpropan-1-one (3d):

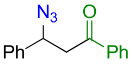

3d

Compound **3d** was synthesized according to the general procedure using 3.0 equiv PhIO (0.9 mmol, 198.2mg) as a colorless oil in 58% yield; However, if this compound was stored at -20 °C for 12 h, it became a white solid. ^{1}H NMR (400 MHz, $CDCl_3$): δ 7.92–7.95 (m, 2H), 7.55–7.58 (m, 1H), 7.42-7.49 (m 2H), 7.36-7.42 (m, 4H), 7.30–7.35 (m, 1H), 5.26 (dd, *J* = 4.6, 8.7 Hz, 1H), 3.55 (dd, *J* = 8.7, 17.3 Hz, 1H), 3.25 (dd, *J* = 4.7, 17.3 Hz, 1H); ^{13}C NMR (100 MHz, $CDCl_3$): δ 196.4, 139.2, 136.4, 133.5, 128.9, 128.7, 128.4, 128.1, 126.8, 61.5, 45.2; HRMS (ESI+) exact mass calculated for $[M+Na]^+$ ($C_{15}H_{13}N_3NaO$) requires m/z 274.0956, found m/z 274.0959.

1-azido-1-(p-tolyl)hexan-3-one (3e):

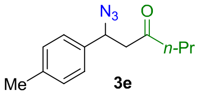

3e

Compound **3e** was synthesized according to the general procedure using 3.5 equiv PhIO (1.05 mmol, 231.6 mg) as a colorless oil in 82% yield; ^{1}H NMR (400 MHz, $CDCl_3$): δ 7.19 (q, *J* = 8.3 Hz, 4H), 5.01 (dd, *J* = 4.9, 8.9 Hz, 1H), 2.93 (dd, *J* = 8.9, 16.8 Hz, 1H), 2.68 (dd, *J* = 4.9, 16.8 Hz, 1H), 2.30–2.45 (m, 2H), 2.34 (s, 3H), 1.54–1.64 (m, 2H), 0.89 (t, *J* = 7.4 Hz, 3H); ^{13}C NMR (100 MHz, $CDCl_3$): δ 207.3, 138.2, 135.8, 129.5, 126.6, 61.0, 48.7, 45.4, 21.0, 16.9, 13.5; HRMS (ESI+) exact mass calculated for $[M+Na]^+$ ($C_{13}H_{17}N_3NaO$) requires m/z 254.1269, found m/z 254.1269.

1-azido-1-(o-tolyl)hexan-3-one (3f):

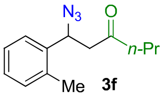

3f

Compound **3f** was synthesized according to the general procedure using 3.0 equiv PhIO (0.9 mmol, 198.0 mg) as a colorless oil in 62% yield; ^{1}H NMR (400 MHz, $CDCl_3$): δ 7.32–7.34 (m, 1H), 7.17–7.26 (m, 3H), 5.31 (dd, *J* = 4.2, 8.8 Hz, 1H), 2.95 (dd, *J* = 9.2, 17.1 Hz, 1H), 2.68 (dd, *J* = 4.3, 17.1 Hz, 1H), 2.34–2.48 (m, 2H), 2.40 (s, 3H), 1.57–1.66 (m, 2H), 0.91 (t, *J* = 7.4 Hz, 3H); ^{13}C NMR (100 MHz, $CDCl_3$): δ 207.5, 137.0, 135.3, 130.9, 128.1, 126.5, 126.1, 57.6, 47.9, 45.5, 19.2, 17.0, 13.6; HRMS (ESI+) exact mass calculated for $[M+Na]^+$ ($C_{13}H_{17}N_3NaO$) requires m/z 254.1269, found m/z 254.1274.

1-azido-1-(4-methoxyphenyl)hexan-3-one (3g):

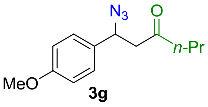

3g

Compound **3g** was synthesized according to the general procedure using 3.0 equiv PhIO (0.9 mmol, 198.1 mg) as a colorless oil in 71% yield; ^{1}H NMR (400 MHz, $CDCl_3$): δ 7.25 (d, *J* = 8.7 Hz, 2H), 6.90 (d, *J* = 8.7 Hz, 2H), 5.00 (dd, *J* = 5.1, 8.8 Hz, 1H), 3.80 (s, 3H), 2.94 (dd, *J* = 8.8, 16.8 Hz, 1H), 2.69 (dd, *J* = 5.1, 16.8 Hz, 1H), 2.30-2.45 (m, 2H), 1.54–1.64 (m, 2H), 0.89 (t, *J* = 7.4 Hz, 3H); ^{13}C NMR (100 MHz, $CDCl_3$): δ 207.4, 159.6, 130.9, 128.0, 114.2, 60.8, 55.2, 48.7, 45.5, 16.9, 13.6; HRMS (ESI+) exact mass calculated for $[M+Na]^+$ ($C_{13}H_{17}N_3NaO_2$) requires m/z 270.1218, found m/z 270.1219.

1-([1,1'-biphenyl]-4-yl)-1-azidohexan-3-one (3h):

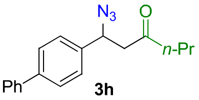

3h

Compound **3h** was synthesized according to the general procedure using 3.5 equiv PhIO (1.05 mmol, 231.0 mg) as a colorless oil in 60% yield; ^{1}H NMR (400 MHz, $CDCl_3$): δ 7.54-7.63 (m, 4H), 7.33-7.45 (m, 5H), 5.10 (dd, *J* = 4.9, 8.9 Hz, 1H), 2.98 (dd, *J* = 8.9, 17.0 Hz, 1H), 2.74 (dd, *J* = 4.9, 17.0 Hz, 1H), 2.32-2.49 (m, 2H), 1.56–1.65 (m, 2H), 0.90 (t, *J* = 7.4 Hz, 3H); ^{13}C NMR (100 MHz, $CDCl_3$): δ 207.3, 141.3, 140.3, 137.9, 128.8, 127.6, 127.5, 127.2, 127.0, 61.0, 48.8, 45.4, 16.9, 13.6; HRMS (ESI+) exact mass calculated for $[M+Na]^+$ ($C_{18}H_{19}N_3NaO$) requires m/z 316.1426, found m/z 316.1431.

1-azido-1-(naphthalen-2-yl)hexan-3-one (3i):

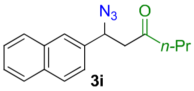

3i

Compound **3i** was synthesized according to the general procedure using 3.5 equiv PhIO (1.05 mmol, 231.6 mg) as a colorless oil in 53% yield; ^{1}H NMR (400 MHz, $CDCl_3$): δ 7.78–7.86 (m, 4H), 7.46-7.51 (m, 2H), 7.42 (dd, *J* = 1.5, 8.5 Hz, 1H), 5.22 (dd, *J* = 4.8, 8.8 Hz, 1H), 3.02 (dd, *J* = 8.9, 17.0 Hz, 1H), 2.77 (dd, *J* = 4.8, 17.0 Hz, 1H), 2.30–2.46 (m, 2H), 1.55–1.64 (m, 2H), 0.88 (t, *J* = 7.4 Hz, 3H); ^{13}C NMR (100 MHz, $CDCl_3$): δ 207.3, 136.3, 133.1, 128.9, 128.0, 127.7, 126.5, 126.4, 126.0, 124.1, 61.4, 48.8, 45.4, 16.9, 13.5; HRMS (ESI+) exact mass calculated for $[M+Na]^+$ ($C_{16}H_{17}N_3NaO$) requires m/z 290.1269, found m/z 290.1267.

1-azido-1-(2-chlorophenyl)hexan-3-one (3j):

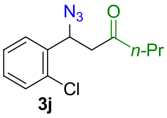

3j

Compound **3j** was synthesized according to the general procedure using 3.5 equiv PhIO (1.05 mmol, 232.0 mg) as a colorless oil in 49% yield; ^{1}H NMR (400 MHz, $CDCl_3$): δ 7.46 (dd, *J* = 1.7, 7.6 Hz, 1H), 7.39 (dd, *J* = 1.4, 7.8 Hz, 1H), 7.25–7.33 (m, 2H), 5.55 (dd, *J* = 3.8, 9.2 Hz, 1H), 2.74-2.87 (m, 2H), 2.43 (t, *J* = 7.3 Hz, 2H), 1.60–1.69 (m, 2H), 0.93 (t, *J* = 7.4 Hz, 3H); ^{13}C NMR (100 MHz, $CDCl_3$) δ 207.0, 137.0, 132.3, 130.0, 129.3, 127.7, 127.4, 57.9, 47.8, 45.1, 17.1, 13.6; HRMS (ESI+) exact mass calculated for $[M+Na]^+$ ($C_{12}H_{14}N_3ClNaO$) requires m/z 274.0723, found m/z 274.0719.

1-azido-1-(4-fluorophenyl)hexan-3-one (3k):

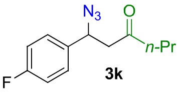

3k

Compound **3k** was synthesized according to the general procedure using 3.0 equiv PhIO (0.9

mmol, 198.9 mg) as a colorless oil in 70% yield; ^{1}H NMR (400 MHz, $CDCl_3$): δ 7.28–7.33 (m, 2H), 7.03–7.09 (m, 2H), 5.05 (dd, *J* = 5.1, 8.6 Hz, 1H), 2.93 (dd, *J* = 8.6, 17.0 Hz, 1H), 2.69 (dd, *J* = 5.2, 17.0 Hz, 1H), 2.30–2.46 (m, 2H), 1.55–1.64 (m, 2H), 0.89 (t, *J* = 7.4 Hz, 3H); ^{13}C NMR (100 MHz, $CDCl_3$): δ 207.1, 162.5 (d, *J* = 245.9 Hz), 134.8 (d, *J* = 3.3 Hz), 128.5 (d, *J* = 8.2 Hz), 115.8 (d, *J* = 21.5 Hz), 60.5, 48.9, 45.4, 16.9, 13.5; HRMS (ESI+) exact mass calculated for $[M+Na]^+$ ($C_{12}H_{14}N_3FNaO$) requires m/z 258.1019, found m/z 258.1017.

1-azido-1-(4-bromophenyl)hexan-3-one (3l):

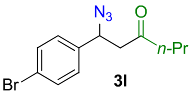

Compound **3l** was synthesized according to the general procedure using 3.5 equiv PhIO (1.05 mmol, 232.0 mg) as a colorless oil in 59% yield; ^{1}H NMR (400 MHz, $CDCl_3$): δ 7.55–7.47 (d, *J* = 8.4, 2H), 7.21 (d, *J* = 8.4 Hz, 2H), 5.03 (dd, *J* = 5.1, 8.6 Hz, 1H), 2.92 (dd, *J* = 8.6, 17.1 Hz, 1H), 2.68 (dd, *J* = 5.1, 17.1 Hz, 1H), 2.30-2.46 (m, 2H), 1.55–1.64 (m, 2H), 0.90 (t, *J* = 7.4 Hz, 3H); ^{13}C NMR (100 MHz, $CDCl_3$): δ 207.0, 138.1, 132.0, 128.4, 122.3, 60.6, 48.8, 45.4, 16.9, 13.6; HRMS (ESI+) exact mass calculated for $[M+Na]^+$ ($C_{12}H_{14}N_3BrNaO$) requires m/z 318.0218, found m/z 318.0213.

1-azido-1-(2,5-dimethylphenyl)hexan-3-one (3m):

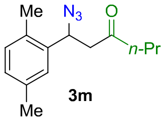

Compound **3m** was synthesized according to the general procedure using 3.5 equiv PhIO (1.05 mmol, 231.8 mg) as a colorless oil in 70% yield; ^{1}H NMR (400 MHz, $CDCl_3$): δ 7.13 (s, 1H), 7.02-7.11 (m, 2H), 5.28 (dd, *J* = 4.1, 9.3 Hz, 1H), 2.94 (dd, *J* = 9.3, 17.1 Hz, 1H), 2.65 (dd, *J* = 4.1, 17.1 Hz, 1H), 2.36–2.45 (m, 2H), 2.32 (s, 3H), 2.29 (s, 3H), 1.57–1.66 (m, 2H), 0.91 (t, *J* = 7.4 Hz, 3H); ^{13}C NMR (100 MHz, $CDCl_3$): δ 207.5, 136.8, 136.0, 132.0, 130.8, 128.8, 126.7, 57.6, 48.0, 45.4, 21.0, 18.7, 17.0, 13.6; HRMS (ESI+) exact mass calculated for $[M+Na]^+$ ($C_{14}H_{19}N_3NaO$) requires m/z 268.1426, found m/z 268.1423.

1-azido-1-(2-bromo-4-methylphenyl)hexan-3-one (3n):

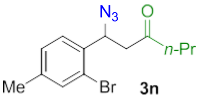

Compound **3n** was synthesized according to the general procedure using 3.5 equiv PhIO (1.05 mmol, 231.7 mg) as a colorless oil in 55% yield; ^{1}H NMR: (400 MHz, $CDCl_3$) δ 7.40 (s, 1H), 7.31 (d, *J* = 7.9 Hz, 1H), 7.15 (d, *J* = 7.7 Hz, 1H), 5.48 (dd, *J* = 4.2, 8.9 Hz, 1H), 2.72–2.84 (m, 2H), 2.42 (t, *J* = 7.3 Hz, 2H), 2.32 (s, 3H), 1.59–1.69 (m, 2H), 0.93 (t, *J* = 7.4 Hz, 3H); ^{13}C NMR (100 MHz, $CDCl_3$): δ 207.0, 139.9, 135.4, 133.6, 128.8, 127.6, 122.3 59.9, 48.0, 45.1, 20.7, 17.0, 13.6; HRMS (ESI+) exact mass calculated for $[M+Na]^+$ ($C_{13}H_{16}N_3BrNaO$) requires m/z 332.0374, found m/z 332.0373.

1-azido-1-mesitylhexan-3-one (3o):

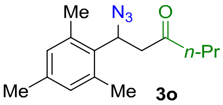

Compound **3o** was synthesized according to the general procedure using 6.0 equiv PhIO (1.8 mmol, 398.0 mg) as a colorless oil in 73% yield; ^{1}H NMR (400 MHz, $CDCl_3$): δ 6.85 (s, 2H), 5.67 (dd, *J* = 4.2, 9.0 Hz, 1H), 3.10 (dd, *J* = 9.1, 17.2 Hz, 1H), 2.60 (dd, *J* = 4.2, 17.2 Hz, 1H), 2.38–2.48 (m, 2H), 2.42 (s, 6H), 2.25 (s, 3H), 1.56-1.65 (m, 2H) 0.90 (t, *J* = 7.4 Hz, 3H); ^{13}C NMR (100 MHz, $CDCl_3$): δ 207.6, 137.6, 136.4, 131.9, 130.2, 56.8, 46.4, 45.4, 20.7, 20.7, 17.00, 13.6; HRMS (ESI+) exact mass calculated for $[M+Na]^+$ ($C_{15}H_{21}N_3NaO$) requires m/z 282.1582, found m/z 282.1578.

1-azido-1,1-diphenylhexan-3-one (3p):

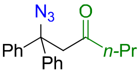

3p

Compound **3p** was synthesized according to the general procedure using 3.5 equiv PhIO (1.05 mmol, 231.9 mg) as a colorless oil in 77% yield; ^{1}H NMR (400 MHz, $CDCl_3$): δ 7.27-7.35 (m,

10H), 3.43 (s, 2H), 2.10 (t, J = 7.2 Hz, 2H), 1.37–1.43 (m, 2H), 0.74 (t, J = 7.4 Hz, 3H); ^{13}C NMR (100 MHz, $CDCl_3$): δ 206.6, 142.1, 128.4 127.8, 126.8, 70.4, 51.2, 46.1, 16.7, 13.4; HRMS (ESI+) exact mass calculated for $[M+Na]^+$ ($C_{18}H_{19}N_3NaO$) requires m/z 316.1426, found m/z 316.1430.

1-azido-1-cyclohexyl-1-phenylhexan-3-one (3q):

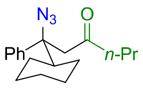

3q

. Compound **3q** was synthesized according to the general procedure using 6 equiv PhIO (1.8 mmol, 396.0 mg) as a colorless oil in 50% yield; ^{1}H NMR (400 MHz, $CDCl_3$): δ 7.22–7.35 (m, 5H), 3.22(d, J = 15.8 Hz, 1H), 3.13 (d, J = 15.8 Hz, 1H), 2.13– 2.28 (m, 2H), 1.62–1.91 (m, 5H), 1.40-1.56 (m, 3H), 0.93–1.25 (m, 5H), 0.75 (t, J = 7.4 Hz, 3H); ^{13}C NMR (100 MHz, $CDCl_3$): δ 207.2, 140.2, 128.1, 127.1, 126.4, 70.9, 48.5, 48.1, 46.0, 27.6, 26.4, 26.1, 16.7, 13.4; HRMS (ESI+) exact mass calculated for $[M+Na]^+$ ($C_{18}H_{25}N_3NaO$) requires m/z 322.1895, found m/z 322.1890.

2-azido-2-phenylheptan-4-one (3r):

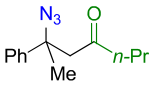

3r

Compound **3r** was synthesized according to the general procedure using 3.5 equiv PhIO (1.05 mmol, 231.2 mg) as a colorless oil in 76% yield; ^{1}H NMR (400 MHz, $CDCl_3$): δ 7.34–7.42 (m, 4H), 7.25-7.30 (m, 1H), 2.87 (s, 2H), 2.24-2.33 (m, 1H), 2.13-2.22 (m, 1H), 1.83 (s, 3H), 1.42–1.51 (m, 2H), 0.80 (t, J = 7.4 Hz, 3H); ^{13}C NMR (100 MHz, $CDCl_3$): δ 207.3, 142.7, 128.6, 127.7, 125.4, 64.6, 53.5, 46.2, 24.5, 16.7, 13.4; HRMS (ESI+) exact mass calculated for $[M+Na]^+$ ($C_{13}H_{17}N_3NaO$) requires m/z 254.1269, found m/z 254.1264.

2-azido-2-(4-(trifluoromethyl)phenyl)heptan-4-one (3s):

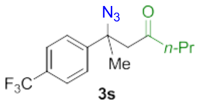

3s

Compound **3s** was synthesized according to the general procedure using 4.0 equiv PhIO (1.2 mmol, 264.1 mg) as a colorless oil in 55% yield; ^{1}H NMR: (400 MHz, $CDCl_3$) δ 7.63 (d, J = 8.3 Hz, 2H), 7.55 (d, J = 8.3 Hz, 2H), 2.92 (s, 2H), 2.19-2.35 (m, 2H), 1.86 (s, 3H), 1.45–1.54 (m, 2H), 0.82 (t, J = 7.4 Hz, 3H); ^{13}C NMR (100 MHz, $CDCl_3$): δ 206.7, 146.9, 130.0, 126.0, 125.6, 124.0 (q, J = 270 Hz), 64.2, 53.1, 46.2, 24.7, 16.8, 13.5; HRMS (ESI+) exact mass calculated for $[M+Na]^+$ ($C_{14}H_{16}F_3N_3NaO$) requires m/z 322.1143, found m/z 322.1149.

1-azido-1,2-diphenylhexan-3-one (3t):

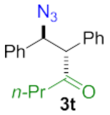

3t

Compound **3t** (from cis-Stilbene) was synthesized according to the general procedure using 6.0 equiv PhIO (1.8 mmol, 396.6 mg) and 1 mol% catalyst (0.003 mmol, 1.7mg) as a colorless oil in 54% yield; ^{1}H NMR (400 MHz, $CDCl_3$): δ 6.96-7.05 (m, 4H), 7.10-7.17 (m, 6H), 5.18 (d, J = 10.8 Hz, 1H), 3.95 (d, J = 10.9 Hz, 1H), 2.38–2.54 (m, 2H), 1.50–1.69 (m, 2H), 0.84 (t, J = 7.5 Hz, 3H); ^{13}C NMR (100 MHz, $CDCl_3$): δ 208.2, 137.0, 134.0, 128.9, 128.6, 128.3, 128.1, 127.7, 127.5, 67.7, 63.9, 45.1, 16.9, 13.4; HRMS (ESI+) exact mass calculated for $[M+Na]^+$ ($C_{18}H_{19}N_3NaO$) requires m/z 316.1426, found m/z 316.1422.

2-azido-2-(benzo[b]thiophen-2-yl)heptan-4-one (3u) :

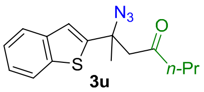

3u

Compound **3u** was synthesized according to the general procedure using 4.0 equiv PhIO (1.2 mmol, 231.6 mg) and 2 mol% catalyst (0.006 mmol, 1.7 mg) as a colorless oil in 72% yield; ^{1}H NMR (400 MHz, $CDCl_3$): δ 7.70–7.81 (m, 2H), 7.28–7.37 (m, 2H), 7.21 (d, J = 0.4 Hz, 1H), 2.98 (s, 2H), 2.24–2.43 (m, 2H), 1.95 (s, 3H), 1.47–1.58 (m, 2H), 0.83 (t, J = 7.4 Hz, 3H); ^{13}C NMR (100 MHz, $CDCl_3$): δ 206.6, 147.9, 139.2, 124.7, 124.5, 123.8, 122.2, 120.9, 63.1, 53.4, 46.2, 24.8, 16.8, 13.5; HRMS (ESI+) exact mass calculated for $[M+Na]^+$ ($C_{15}H_{17}N_3NaOS$) requires m/z 310.0990, found m/z 310.0986.

2-(azido(phenyl)methyl)-*N*,*N*-dibenzyl-3-oxohexanamide (3aa):

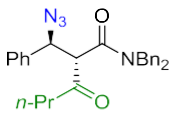

3aa

Compound **3aa** was synthesized according to the general procedure using 3.5 equiv PhIO (1.05 mmol, 231.0 mg) as a white solid in 55% yield; ^{1}H NMR (400 MHz, $CDCl_3$): δ 7.24-7.49 (m, 15H), 5.50 (d, *J* = 10.6 Hz, 1H), 4.93 (d, *J* = 14.7 Hz, 1H), 4.53–4.78 (m, 2H), 4.50 (d, *J* = 15.2 Hz, 1H), 4.20 (d, *J* = 10.6 Hz, 1H), 2.00–2.19 (m, 2H), 1.18–1.27 (m, 2H), 0.59 (t, *J* = 7.4 Hz, 3H); ^{13}C NMR (100 MHz, $CDCl_3$): δ 202.5, 166.9, 136.7, 136.2, 136.0, 129.1, 129.0, 128.7, 128.3, 127.9, 127.9, 127.6, 126.7, 65.5, 62.6, 50.2, 49.3, 42.8, 16.3, 13.1; HRMS (ESI+) exact mass calculated for $[M+H]^+$ ($C_{27}H_{29}N_4O_2$) requires m/z 441.2291, found m/z 441.2295.

2-(azido(phenyl)methyl)-*N*,*N*-diethyl-3-oxohexanamide (3ab):

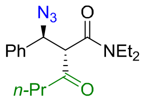

3ab

Compound **3ab** was synthesized according to the general procedure using 4.0 equiv PhIO (1.2 mmol, 264.5 mg) as a colorless oil in 55% yield; ^{1}H NMR (400 MHz, $CDCl_3$): δ 7.32–7.40 (m, 5H), 5.42 (d, *J* = 10.6 Hz, 1H), 4.09 (d, *J* = 10.6 Hz, 1H), 3.53–3.57 (m, 2H), 3.31–3.38 (m, 2H), 2.35-2.43 (m, 1H), 2.12–2.20 (m, 1H), 1.26–1.37 (m, 2H), 1.30 (t, *J* = 7.2 Hz, 3H), 1.16 (t, *J* = 7.2 Hz, 3H), 0.66 (t, *J* = 7.4 Hz, 3H); ^{13}C NMR (100 MHz, $CDCl_3$): δ 202.9, 165.3, 136.4, 129.0, 128.9, 127.9, 65.3, 62.8, 42.4, 42.2, 41.2, 16.4, 14.5, 13.1, 12.8; HRMS (ESI+) exact mass calculated for $[M+H]^+$ ($C_{17}H_{25}N_4O_2$) requires m/z 317.1972, found m/z 317.1969.

2-(azido(4-(trifluoromethyl)phenyl)methyl)-*N*,*N*-diethyl-3-oxohexanamide (3ab):

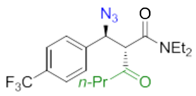

3ac

Compound **3ac** was synthesized according to the general procedure using 4.0 equiv PhIO (1.2 mmol, 264.2 mg) as a light yellow solid in 50% yield; ^{1}H NMR (400 MHz, $CDCl_3$): δ 7.64 (d, *J* = 8.2 Hz, 2H), 7.49 (d, *J* = 8.2 Hz, 2H), 5.47 (d, *J* = 10.5 Hz, 1H), 4.5 (d, *J* = 10.5 Hz, 1H), 3.52–3.61 (m, 2H), 3.35–3.47 (m, 2H), 2.31–2.39 (m, 1H), 2.16-2.24(m, 1H), 1.30-1.41 (m, 2H), 1.32 (t, *J* = 7.2 Hz, 3H), 1.18 (t, *J* = 7.2 Hz, 3H), 0.69 (t, *J* = 7.4 Hz, 3H); ^{13}C NMR (100 MHz, $CDCl_3$): δ 202.3, 165.0, 140.9, 131.1, 128.4, 126.0 (q, *J* = 4.0 Hz), 123.8 (q, *J* = 270.0 Hz), 64.7, 62.6 , 42.6, 41.4, 16.5, 14.6, 13.2, 12.8; HRMS (ESI+) exact mass calculated for $[M+H]^+$ ($C_{18}H_{24}F_3N_4O_2$) requires m/z 385.1846, found m/z 385.1843.

2-(azido(2-bromophenyl)methyl)-*N*,*N*-diethyl-3-oxohexanamide (3ad):

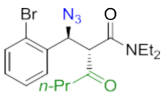

3ad

Compound **3ad** was synthesized according to the general procedure using 4.0 equiv PhIO (1.2 mmol, 265.0 mg) as a white solid in 62% yield; ^{1}H NMR (400 MHz, $CDCl_3$): δ 7.61 (d, *J* = 7.9 Hz, 1H), 7.32–7.36 (m, 2H), 7.18–7.22 (m, 1H), 6.01 (d, *J* = 10.1 Hz, 1H), 4.22 (d, *J* = 10.1 Hz, 1H), 3.46–3.58 (m, 2H), 3.26–3.41 (m, 2H), 2.44–2.52 (m, 1H), 2.21–2.29 (m, 1H), 1.29–1.44 (m, 2H), 1.25 (t, *J* = 7.2 Hz, 3H), 1.16 (t, *J* = 7.1 Hz, 3H), 0.67 (t, *J* = 7.4 Hz, 3H); ^{13}C NMR (100 MHz, $CDCl_3$): δ 203.4, 165.1, 135.6, 133.6, 130.4, 129.7, 128.1, 124.2, 63.3, 61.6, 42.3, 41.7 41.3, 16.4, 14.5, 13.1, 12.8; HRMS (ESI+) exact mass calculated for $[M+H]^+$ ($C_{17}H_{24}BrN_4O_2$) requires m/z 395.1077, found m/z 395.1074.

2-(azido(3-fluorophenyl)methyl)-*N*,*N*-diethyl-3-oxohexanamide (3ae):

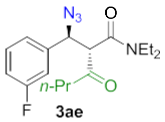

3ae

Compound **3ae** was synthesized according to the general procedure using 4.0 equiv PhIO (1.2 mmol, 264.0 mg) as a white solid in 50% yield; ^{1}H NMR (400 MHz, $CDCl_3$): δ 7.33-7.38 (m, 1H), 7.15 (d, *J* = 7.7 Hz, 1H), 7.02–7.08 (m, 2H), 5.41 (d, *J* = 10.6 Hz, 1H), 4.03 (d, *J* = 10.6 Hz, 1H), 3.52-3.61 (m, 2H), 3.33–3.44 (m, 2H), 2.33–2.41 (m, 1H), 2.16-2.24 (m, 1H), 1.26-1.42 (m, 2H), 1.30 (t, *J* = 7.2 Hz, 3H), 1.17 (t, *J* = 7.1 Hz, 3H), 0.69 (t, *J* = 7.4 Hz, 3H); ^{13}C NMR (100 MHz,

$CDCl_3$): δ 202.5, 165.0, 162.9 (d, *J* = 246.0 Hz), 139.1 (d, *J* = 6.0 Hz), 130.5 (d, *J* = 9.0 Hz), 123.7 (d, *J* = 3.0 Hz), 116.1 (d, *J* = 20.0 Hz),114.8 (d, *J* = 22 Hz), 64.7, 62.6, 42.5, 41.2, 16.4, 14.5, 13.2, 12.8; HRMS (ESI+) exact mass calculated for $[M+H]^+$ ($C_{17}H_{24}FN_4O_2$) requires m/z 335.1878, found m/z 335.1882.

2-(azido(4-fluorophenyl)methyl)-*N,N*-diethyl-3-oxohexanamide (3af):

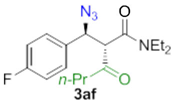

3af

Compound **3af** was synthesized according to the general procedure using 4.0 equiv PhIO (1.2 mmol, 264.0 mg) as a white solid in 59% yield; ^1H NMR (400 MHz, $CDCl_3$): δ 7.34-7.37 (m, 2H), 7.05-7.09 (t, *J* = 8.3 Hz, 2H), 5.42 (d, *J* = 10.6 Hz, 1H), 4.04 (d, *J* = 10.6 Hz, 1H), 3.53-3.62 (m, 2H), 3.32–3.44 (m, 2H), 2.34–2.42 (m, 1H), 2.13–2.21 (m, 1H), 1.28–1.40 (m, 2H), 1.30 (t, *J* = 7.0 Hz, 3H), 1.17 (t, *J* = 6.9 Hz, 3H), 0.68 (t, *J* = 7.3 Hz, 3H); ^{13}C NMR (100 MHz, $CDCl_3$): δ 202.7, 165.2, 162.8 (d, *J* = 247.0 Hz), 132.4 (d, *J* = 3.0 Hz), 129.7 (d, *J* = 9.0 Hz), 116.0 (d, *J* = 22.0 Hz), 64.5, 62.9, 42.4, 42.3, 41.2, 16.4, 14.5, 13.1, 12.7; HRMS (ESI+) exact mass calculated for $[M+H]^+$ ($C_{17}H_{24}FN_4O_2$) requires m/z 335.1878, found m/z 335.1883.

2-(azido(4-chlorophenyl)methyl)-*N,N*-diethyl-3-oxohexanamide (3ag):

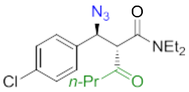

3ag

Compound **3ag** was synthesized according to the general procedure using 4.0 equiv PhIO (1.2 mmol, 264.2 mg) as a white solid in 53% yield; ^1H NMR (400 MHz, $CDCl_3$): δ 7.36 (d, *J* = 8.5 Hz, 2H), 7.30 (d, *J* = 8.5 Hz, 2H), 5.40 (d, *J* = 10.6 Hz, 1H), 4.02 (d, *J* = 10.6 Hz, 1H), 3.52–3.58 (m, 2H), 3.32–3.44 (m, 2H), 2.34–2.41 (m, 1H), 2.13–2.21 (m, 1H), 1.26-1.41 (m, 2H), 1.30 (t, *J* = 7.2 Hz, 3H), 1.17 (t, *J* = 7.1 Hz, 3H), 0.69 (t, *J* = 7.4 Hz, 3H); ^{13}C NMR (100 MHz, $CDCl_3$): δ 202.6, 165.1, 135.1, 134.8, 129.2, 129.1, 64.5, 62.7, 42.4, 42.3, 41.2, 16.4, 14.5, 13.2, 12.8; HRMS (ESI+) exact mass calculated for $[M+H]^+$ ($C_{17}H_{24}ClN_4O_2$) requires m/z 351.1582, found m/z 351.1577.

2-(azido(3,4-difluorophenyl)methyl)-*N,N*-diethyl-3-oxohexanamide (3ah):

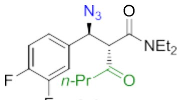

3ah

Compound **3ah** was synthesized according to the general procedure using 4.0 equiv PhIO (1.2 mmol, 264.9 mg) as a white solid in 67% yield; ^1H NMR (400 MHz, $CDCl_3$): δ 7.10-7.22 (m, 3H), 5.39 (d, *J* = 10.5 Hz, 1H), 3.97 (d, *J* = 10.5 Hz, 1H), 3.51–3.61 (m, 2H), 3.34–3.45 (m, 2H), 2.32-2.40 (m,1H), 2.17-2.25 (m, 1H), 1.35–1.43 (m, 2H), 1.31 (t, *J* = 7.2 Hz, 3H, 1.17 (t, *J* = 7.1 Hz, 3H), 0.71 (t, *J* = 7.4 Hz, 3H); ^{13}C NMR (100 MHz, $CDCl_3$): δ 202.3, 164.9, 150.5 (dd, *J* = 5.0, 254.0 Hz), 150.4 (dd, *J* = 5.0, 240.0 Hz), 134.0 (t, *J* = 5.0 Hz), 124.2 (dd, *J* = 3.0, 6.0 Hz), 117.8 (d, *J* = 17.0 Hz), 116.9 (d, *J* = 17.0 Hz), 64.3, 62.7, 42.5, 41.3, 16.5, 14.5, 13.2, 12.8; HRMS (ESI+) exact mass calculated for $[M+H]^+$ ($C_{17}H_{23}FN_4O_2$) requires m/z 353.1784, found m/z 353.1782.

2-(azido(2-methoxyphenyl)methyl)-*N,N*-diethyl-3-oxohexanamide (3ai):

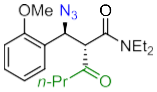

3ai

Compound **3ai** was synthesized according to the general procedure using 4.0 equiv PhIO (1.2 mmol, 264.2 mg) as a white solid in 54% yield; ^1H NMR (400 MHz, $CDCl_3$): δ 7.22-7.37 (m, 2H), 6.86-7.01 (m, 2H), 5.82 (d, *J* = 10.7 Hz, 1H), 4.33 (d, *J* = 10.7 Hz, 1H), 3.88 (s, 3H), 3.49–3.62 (m, 2H), 3.29–3.46 (m, 2H), 2.41-2.52 (m, 1H), 2.17-2.27 (m, 1H), 1.22–1.42 (m, 2H), 1.28 (t, *J* = 7.2 Hz, 3H), 1.16 (t, *J* = 7.1 Hz, 3H), 0.66 (t, *J* = 7.4 Hz, 3H); ^{13}C NMR (100 MHz, $CDCl_3$): δ 203.7, 165.8, 157.0, 130.2, 129.3, 124.0, 120.9, 111.1, 61.1, 60.0, 55.4, 42.6, 41.5, 41.1, 16.3, 14.4, 13.1, 12.8; HRMS (ESI+) exact mass calculated for $[M+H]^+$ ($C_{18}H_{27}N_4O_3$) requires m/z 347.2078, found m/z 347.2081.

2-(azido(o-tolyl)methyl)-*N,N*-diethyl-3-oxohexanamide (3aj):

Compound **3aj** was synthesized according to the general procedure using 4.0 equiv PhIO (1.2 mmol, 264.4 mg) as

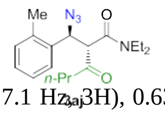

3aj

a colorless oil in 57% yield; ^{1}H NMR (400 MHz, $CDCl_3$): δ 7.21-7.26 (m, 4H), 5.70 (d, *J* = 10.6 Hz, 1H), 4.27 (d, *J* = 10.6 Hz, 1H), 3.52-3.67 (m, 2H), 3.33–3.46 (m, 2H), 2.53 (s, 3H), 2.33-2.41 (m, 1H), 2.03-2.11 (m, 1H), 1.25– 1.37 (m, 2H), 1.32 (t, *J* = 7.2 Hz, 3H), 1.17 (t, *J* = 7.1 Hz, 3H), 0.63 (t, *J* = 7.4 Hz, 3H); ^{13}C NMR (100 MHz, $CDCl_3$): δ 202.9, 165.4, 136.9, 134.3, 131.3, 128.8, 127.3, 126.5, 61.6 , 61.1, 42.4, 42.1, 41.3, 19.7, 16.4, 14.5, 13.1, 12.8; HRMS (ESI+) exact mass calculated for $[M+H]^+$ ($C_{18}H_{27}N_4O_2$) requires m/z 331.2129, found m/z 331.2124.

2-(azido(m-tolyl)methyl)-N,N-diethyl-3-oxohexanamide (3ak):

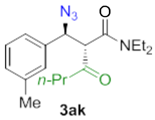

3ak

Compound **3ak** was synthesized according to the general procedure using 4.0 equiv PhIO (1.2 mmol, 264.9 mg) as a colorless oil in 67% yield; ^{1}H NMR (400 MHz, $CDCl_3$): δ 7.20–7.29 (m, 1H), 7.10-7.17 (m, 3H), 5.37 (d, *J* = 10.6 Hz, 1H), 4.07 (d, *J* = 10.6 Hz, 1H), 3.5–3.64 (m, 2H), 3.29-3.43 (m, 2H), 2.33–2.45 (m, 1H), 2.36 (s, 3H), 2.12– 2.22 (m, 1H), 1.26–1.40 (m, 2H), 1.29 (t, *J* = 7.2 Hz, 3H), 1.16 (t, *J* = 7.1 Hz, 3H), 0.66 (t, *J* = 7.4 Hz, 3H); ^{13}C NMR (100 MHz, $CDCl_3$): δ 203.0, 165.3, 138.7, 136.12, 129.8, 128.8, 128.5, 124.9, 65.3, 62.8, 42.3, 42.1, 41.2, 21.3, 16.4, 14.5, 13.1, 12.8; HRMS (ESI+) exact mass calculated for $[M+H]^+$ ($C_{18}H_{27}N_4O_2$) requires m/z 331.2129, found m/z 331.2134.

2-(azido(naphthalen-2-yl)methyl)-N,N-diethyl-3-oxohexanamide (3al):

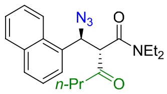

3al

Compound **3al** was synthesized according to the general procedure using 4.0 equiv PhIO (1.2 mmol, 264.1 mg) as a white solid in 70% yield; ^{1}H NMR (400 MHz, $CDCl_3$): δ 8.33 (d, *J* = 8.5 Hz, 1H), 7.81-7.93 (m, 2H), 7.60–7.66 (m, 1H), 7.42–7.58 (m, 3H), 6.24 (d, *J* = 10.3 Hz, 1H), 4.47 (d, *J* = 10.4 Hz, 1H), 3.55–3.72 (m, 2H), 3.30–3.46 (m, 2H), 2.29-2.44 (m, 1H), 1.94-2.10 (m, 1H), 1.31 (t, *J* = 7.2 Hz, 3H), 1.09–1.24 (m, 2H), 1.17 (t, *J* = 7.2 Hz, 3H), 0.48 (t, *J* = 7.4 Hz, 3H); ^{13}C NMR (100 MHz, $CDCl_3$): δ 203.3, 165.4, 134.1, 131.7, 131.2, 129.8, 129.1, 127.0, 126.2, 125.1, 122.9, 62.0, 42.4, 41.8, 41.3, 16.3, 14.5, 13.0, 12.9; HRMS (ESI+) exact mass calculated for $[M+H]^+$ ($C_{21}H_{27}N_4O_2$) requires m/z 367.2129, found m/z 367.2128.

2-(azido(2,3-dimethoxyphenyl)methyl)-N,N-diethyl-3-oxohexanamide (3am):

Compound **3am** was synthesized according to the general procedure using 5.0 equiv PhIO (1.5 mmol, 330.9 mg)

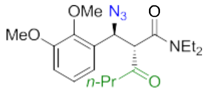

3am

as a white solid in 74% yield; ^{1}H NMR (400 MHz, $CDCl_3$): δ 7.05 (t, *J* = 8.0 Hz, 1H), 6.86–6.94 (m, 2H), 5.71 (d, *J* = 10.8 Hz, 1H), 4.37 (d, *J* = 10.8 Hz, 1H), 3.95 (s, 3H), 3.87 (s, 3H), 3.50–3.64 (m, 2H), 3.32–3.49 (m, 2H), 2.39-2.51 (m, 1H), 2.17-2.28 (m, 1H), 1.26-1.41 (m, 2H), 1.29 (t, *J* = 7.2 Hz, 3H), 1.17 (t, *J* = 7.1 Hz, 3H), 0.67 (t, *J* = 7.4 Hz, 3H; ^{13}C NMR (100 MHz, $CDCl_3$): δ 203.4, 165.8, 152.9, 147.3, 129.7, 124.3, 121.0, 113.0, 61.3, 61.2, 60.8, 55.7, 42.4, 41.8, 41.2, 16.5, 14.5, 13.2, 12.9; HRMS (ESI+) exact mass calculated for $[M+H]^+$ ($C_{19}H_{29}N_4O_4$) requires m/z 377.2183, found m/z 377.2184.

2-(azido(3,4,5-trimethoxyphenyl)methyl)-N,N-diethyl-3-oxohexanamide (3an):

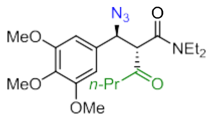

3an

Compound **3an** was synthesized according to the general procedure using 5.0 equiv PhIO (1.5 mmol, 330.2 mg) as a white solid in 78% yield; ^{1}H NMR (400 MHz, $CDCl_3$): δ 6.54 (s, 2H), 5.37 (d, *J* = 10.4 Hz, 1H), 4.02 (d, *J* = 10.4 Hz, 1H), 3.88 (s, 6H), 3.86 (s, 3H), 3.48–3.65 (m, 2H), 3.31–3.46 (m, 2H), 2.31–2.43 (m, 1H), 2.14-2.24 (m, 1H), 1.32–1.43 (m, 2H), 1.29 (t, *J* = 7.2 Hz, 3H), 1.16 (t, *J* = 7.1 Hz, 3H), 0.69 (t, *J* = 7.4 Hz, 3H); ^{13}C NMR (100 MHz, $CDCl_3$): δ 202.9, 165.1, 153.4, 138.3, 131.9, 105.0, 65.4, 62.9, 60.7, 56.2, 42.3, 41.1, 16.4, 14.4, 13.1, 12.7; HRMS (ESI+) exact mass calculated for $[M+H]^+$ ($C_{20}H_{30}N_4NaO_5$) requires m/z 429.2108, found m/z 429.2105.

(8R,9S,13S,14S)-3-(1-azido-3-oxo-3-phenylpropyl)-13-methyl-6,7,8,9,11,12,13,14,15,16-decahydro-17H-cyclopenta[a]phenanthren-17-one (4a):

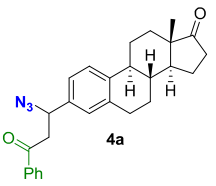

Compound **4a** was synthesized according to the general procedure using 4.0 equiv PhIO (1.2 mmol, 264.4 mg) as a colorless oil in 45% yield; This compound is nearly a 1:1 mixture of diastereomers, which can be separated *by* HPLC on a chiral stationary phase ((IA column, hexane: *i*-PrOH = 95:5, 0.5 mL/min, peak 1: 57.5 min (47%); Peak 2: 67.5 min (53%)). ^{1}H NMR (400 MHz, $CDCl_3$): δ 7.95 (d, *J* = 7.7 Hz, 2H), 7.58 (t, *J* = 7.3 Hz, 1H), 7.47 (t, *J* = 7.6 Hz, 2H), 7.32 (d, *J* = 8.1 Hz, 1H), 7.18 (d, *J* = 8.1 Hz, 1H), 7.13 (s, 1H), 5.20 (dd, *J* = 4.5, 8.7 Hz, 1H), 3.55 (dd, *J* = 8.8, 17.3 Hz, 1H), 3.25 (dd, *J* = 4.5, 17.3 Hz, 1H), 2.90–3.00 (m, 2H), 2.51 (dd, *J* = 8.9, 19.0 Hz, 1H), 2.38–2.46 (m, 1H), 2.26-2.36 (m, 1H), 2.01–2.21 (m, 3H), 1.97 (d, *J* = 11.5 Hz, 1H), 1.42–1.68 (m, 6H), 0.91 (s, 3H); ^{13}C NMR (100 MHz, $CDCl_3$): δ 220.8, 196.5, 140.0, 137.1, 136.7, 136.5, 133.4, 128.7, 128.1, 127.4, 125.9, 124.1, 61.3, 50.5, 47.9, 45.1, 44.3, 37.9, 35.8, 31.5, 29.4, 26.3, 25.6, 21.5, 13.8; HRMS (ESI+) exact mass calculated for [M+H]+ ($C_{27}H_{29}NaN_3O_2$) requires m/z 450.2157, found m/z 450.2156.

(8R,9S,13S,14S)-13-methyl-17-oxo-7,8,9,11,12,13,14,15,16,17-decahydro-6H-cyclopenta[a]phenanthren-3-yl (2-(azido(phenyl)methyl)-3-oxohexanoyl)-L-prolinate (4b):

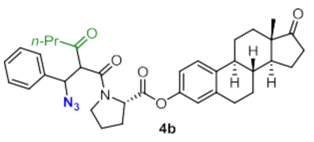

Compound **4b** was synthesized according to the general procedure using 4.0 equiv PhIO (1.2 mmol, 265.7 mg) as a colorless oil in 47% yield; ^{1}H NMR (400 MHz, $CDCl_3$) : δ 7.34–7.41 (m, 5H), 7.25–7.29 (m, 1H), 6.81–6.89 (m, 2H), 5.40 (d, *J* = 10.6 Hz, 1H), 4.82 (dd, *J* = 4.5, 8.5 Hz, 1H), 4.04 (d, *J* = 10.6 Hz, 1H), 3.81 (t, *J* = 6.3 Hz, 2H), 2.84-2.95 (m, 2H), 2.50 (dd, *J* = 8.9, 19.0 Hz), 2.36–2.43 (m, 2H), 2.35–2.26 (m, 3H), 2.20–2.11 (m, 3H), 2.07–1.93 (m, 4H), 1.60 (dd, *J* = 7.7, 10.7 Hz, 2H), 1.56–1.45 (m, 4H), 1.30–1.25 (m, 2H), 0.90 (s, 3H), 0.56 (t, *J* = 7.4 Hz, 3H); ^{13}C NMR (100 MHz, $CDCl_3$) : δ 220.6, 201.5, 170.5, 165.1, 148.4, 138.0, 137.5, 136.4 , 129.0, 128.9, 127.9, 126.3, 121.3, 118.4, 64.7, 64.2, 59.4, 50.4, 47.9, 47.7, 44.1, 42.8, 37.9, 35.8, 31.5, 29.3, 29.2, 26.3, 25.7, 24.9, 21.5, 16.2, 13.8, 13.0; HRMS (ESI+) exact mass calculated for $[M+H]^+$ ($C_{36}H_{43}N_4O_5$) requires m/z 611.3233, found m/z 611.3229.

methyl (2-(azido(phenyl)methyl)-3-oxohexanoyl)-D-prolyl-D-valinate (5a)

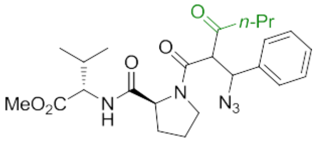

Compound **5a** was synthesized according to the general procedure using 4.0 equiv PhIO (1.2 mmol, 264.5 mg) as a colorless oil in 64% yield; ^{1}H NMR (400 MHz, $CDCl_3$): δ 7.35–7.39 (m, 5H), 7.06 (d, *J* = 8.5 Hz, 1H), 5.40 (d, *J* = 10.6 Hz, 1H), 4.66–4.73 (m, 1H), 4.46 (dd, *J* = 5.0, 8.5 Hz, 1H), 4.07 (d, *J* = 10.6 Hz, 1H), 3.72–3.77 (m, 5H), 2.22-2.29 (m, 2H), 2.10-2.21 (m, 2H), 2.00-2.09 (m, 2H), 1.24–1.37 (m, 2H), 0.91 (d, *J* = 3.3 Hz, 3H), 0.89 (d, *J* = 3.3 Hz, 3H), 0.65 (t, *J* = 7.4 Hz, 3H); ^{13}C NMR (100 MHz, $CDCl_3$): δ 201.4, 172.1, 170.7, 166.1, 136.3, 129.0, 128.9, 127.8, 64.7, 63.7, 60.3, 57.4, 52.0, 48.3, 43.6, 31.0, 27.8, 25.0 , 18.9, 17.6, 16.2, 13.1; HRMS (ESI+) exact mass calculated for $[M+H]^+$ ($C_{24}H_{34}N_5O_5$) requires m/z 472.2560, found m/z 472.2565.

methyl (2-(azido(phenyl)methyl)-3-oxohexanoyl)-L-prolyl-D-phenylalaninate (5b):

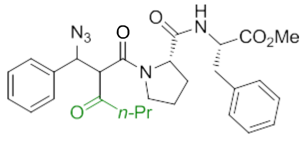

Compound **5b** was synthesized according to the general procedure using 4.0 equiv PhIO (1.2 mmol, 264.0 mg) as a colorless oil in 44% yield; ^{1}H NMR (400 MHz, $CDCl_3$): δ 7.34–7.40 (m, 5H), 7.22–7.29 (m, 3H), 7.12–7.17 (m, 2H), 6.84 (d, *J* = 7.6 Hz, 1H), 5.38 (d, *J* = 10.5 Hz, 1H), 4.74-4.81 (m, 1H), 4.60–4.65 (m, 1H), 4.03 (d, *J* = 10.5 Hz, 1H), 3.64–3.73 (m, 5H), 3.03-3.15 (m, 2H), 2.18–2.28 (m, 3H),

1.96-2.06 (m, 3H), 1.25–1.35 (m, 2H), 0.63 (t, *J* = 7.4 Hz, 3H); ^{13}C NMR (100 MHz, $CDCl_3$): δ 201.8, 171.6, 170.5, 166.0, 136.3, 135.8, 129.2, 129.0, 128.9, 128.4, 127.8, 126.7, 64.7, 63.9, 60.4, 53.5, 52.2, 48.1, 43.3, 37.9, 28.2, 24.7, 16.3, 13.1; HRMS (ESI+) exact mass calculated for $[M+H]^+$ ($C_{28}H_{34}N_5O_5$) requires m/z 520.2560, found m/z 520.2557.

5. Mechanistic Study

(1) the generation of acyl radical：

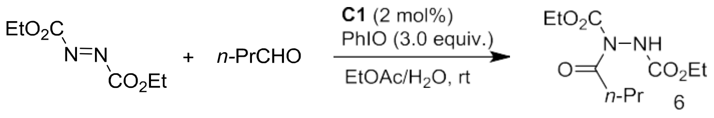

To a reaction mixture of catalyst **C1** (1.2 mg, 0.002 mmol, 2 mol%) and PhIO (66.8mg, 0.3 mmol, 3.0 equiv.) in a 10 mL-Schlenk tube were added 1.0 mL H_2O, *n*-butylaldehyde (44.0 uL, 0.5 mmol, 5.0 equiv.), and diethyl (E)-diazene-1,2-dicarboxylate (16.0 uL, 0.1 mmol, 1.0 equiv.) under a nitrogen atmosphere. Then 1.0 mL EtOAc was added to the above reaction mixture and the reaction was stirred at room temperature. After the completion of the reaction as judged by TLC analysis, the reaction mixture was extracted with EA (10.0 mL×1) and CH_2Cl_2 (8.0 mL×2). The combined organic la yer was dried over anhydrous sodium sulfate and evaporated under reduced pressure to afford the crude product, which was purified by flash chromatography on silica gel to give the desired products **6** (23.5 mg, 0.095mmol, 95%). Its spectral data obtained were identical with those reported in literature.[11]

(2) Radical clock experiment：

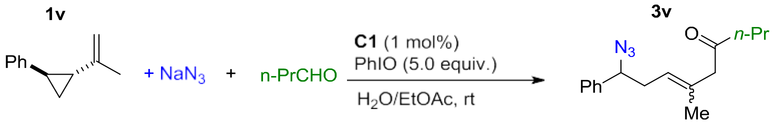

To a reaction mixture of catalyst **C1** (1.7 mg, 0.003 mmol, 1 mol%) and PhIO (339.9 mg, 1.5 mmol, 5.0 equiv.) were added a aqueous solution of NaN_3 (4.0 mL, 0.3 M, 1.2 mmol, 4.0 equiv.), *n*-butylaldehyde (132.0 uL, 1.5 mmol, 5.0 equiv.), and olefin **1u** (47.5mg, 0.3 mmol, 1.0 equiv.) under a nitrogen atmosphere. Then 2.4 mL EtOAc was added to the above reaction mixture and the reaction was stirred at room temperature. After the completion of the reaction as judged by TLC analysis, the reaction mixture was extracted with EA (20.0 mL×1) and CH_2Cl_2 (15.0 mL×2). The combined organic layer was dried over anhydrous sodium sulfate and evaporated under reduced pressure to afford the crude product, which was purified by flash chromatography on silica gel to obtain a colourless oil **3v** (8.8 mg, 0.03 mmol, 11%). ^{1}H NMR (400 MHz, $CDCl_3$): δ 7.28-7.40 (m, 5H), 5.23 (t, *J* = 6.3 Hz, 1H), 4.48 (t, *J* = 7.2 Hz, 1H), 3.03 (s, 2H), 2.45–2.63 (m, 2H), 2.32 (t, *J* = 7.4 Hz, 2H), 1.50-1.63 (m, 2H), 1.58 (s, 3H), 0.88 (t, *J* = 7.4 Hz); ^{13}C NMR (100 MHz, $CDCl_3$): δ 209.2, 139.3, 132.6, 128.7, 128.2, 126.8, 124.2, 66.0, 53.8, 43.6, 35.0, 17.1, 16.6, 13.6; HRMS (ESI+) exact mass calculated for $[M+Na]^+$ ($C_{16}H_{21}N_3NaO$) requires m/z 294.1582, found m/z 294.1581.

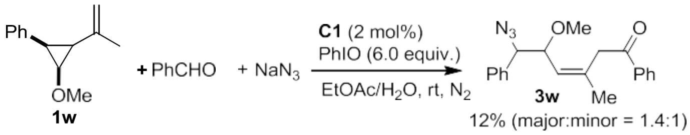

To a reaction mixture of catalyst **C1** (3.4 mg, 0.006 mmol, 2 mol%) and PhIO (399.7 mg, 1.8 mmol, 6.0 equiv.) were added a aqueous solution of NaN_3 (4.0 mL, 0.3 M, 1.2 mmol, 4.0 equiv.), benzaldehyde (150.0 uL, 1.5 mmol, 5.0 equiv.), and olefin **1w** (56.4mg, 0.3 mmol, 1.0 equiv.) under a nitrogen atmosphere. Then 2.4 mL EtOAc was added to the above reaction mixture and the reaction was stirred at room temperature. After the completion of the reaction as judged by TLC analysis, the reaction mixture was extracted with EA (20.0 mL×1) and CH_2Cl_2 (15.0 mL×2). The combined organic layer was dried over anhydrous sodium sulfate and evaporated under reduced pressure to afford the crude product, which was purified by flash chromatography on silica gel to obtain a light yellow oil **3w** (12.2 mg, 0.03 mmol, 12%). Major isomer: ^{1}H NMR (400 MHz, $CDCl_3$): δ 7.83-7.90 (m, 2H), 7.53-7.59 (m, 1H), 7.40-7.50 (m, 2H), 7.21-7.29 (m, 3H), 7.15-7.20 (m, 2H), 5.11 (dq, *J* = 1.2, 9.3 Hz, 1H), 4.40 (d, *J* = 7.9 Hz, 1H), 4.12 (dd, 1H, *J* = 8.1, 9.3 Hz), 3.57 (d, *J* = 15.2 Hz, 1H), 3.51 (d, *J* = 15.2 Hz, 1H), 3.32 (s, 3H), 1.41 (d, *J* = 1.2 Hz, 3H); ^{13}C NMR (100 MHz, $CDCl_3$): δ 197.3, 136.7, 136.4, 136.3, 133.1, 128.5, 128.4, 128.3, 128.2, 127.8, 126.1, 80.2, 69.1 56.3, 49.1, 17.1; HRMS (ESI+) exact mass calculated for $[M+Na]^+$ ($C_{20}H_{21}N_3NaO_2$) requires m/z 358.1531, found m/z 358.1528.

Minor: ^{1}H NMR (400 MHz, $CDCl_3$): δ 7.94-7.99 (m, 2H), 7.53-7.59 (m, 1H), 7.41-7.49 (m, 2H), 7.21-7.29 (m, 5H,), 5.29 (dq, *J* = 1.2, 9.1 Hz, 1H), 4.67 (d, *J* = 4.4 Hz, 1H), 4.13 (dd, 1H, 1H, *J* = 4.3, 9.2 Hz,), 3.73 (d, *J* = 15.2 Hz, 1H), 3.65 (d, *J* = 15.2 Hz, 1H), 3.26 (s, 3H), 1.42 (d, 3H, *J* = 1.3 Hz); ^{13}C NMR (100 MHz, $CDCl_3$): δ 197.5, 137.0, 136.5, 136.2, 133.2, 128.6, 128.4, 128.3, 128.1, 127.7, 125.5, 80.2, 68.8, 56.3, 49.5, 17.0. HRMS (ESI+) exact mass calculated for $[M+Na]^+$ ($C_{20}H_{21}N_3NaO_2$) requires m/z 358.1531, found m/z 358.1528.

(3) competitive experiment:

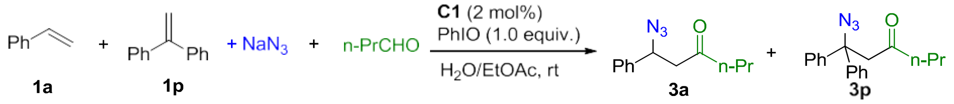

To a reaction mixture of catalyst **C1** (3.5 mg, 0.006 mmol, 2 mol%) and PhIO (137.2 mg, 0.6 mmol, 2.0 equiv.) were added a aqueous solution of NaN_3 (2.0 mL, 0.3 M, 0.6 mmol, 2.0 equiv.), *n*-butylaldehyde (53.0 uL, 0.6 mmol, 2.0 equiv.), olefin **1a** (35.0 uL, 0.3 mmol) and **1p** (53.0 uL, 0.3 mmol) under a nitrogen atmosphere. Then 2.4 mL EtOAc was added to the above reaction mixture and the reaction was stirred at room temperature. When the solid PhIO disappeared, the reaction mixture was extracted with EA (20.0 mL×1) and CH_2Cl_2 (15.0 mL×2). The combined organic layer was dried over anhydrous sodium sulfate and evaporated under reduced pressure to afford the crude product, which was purified by flash chromatography on silica gel to obtain **3a** (5.5 mg, 8%), **3p** (38.5 mg, 46%). ^{1}H NMR ratio of crude mixture for **3p**:**3a** is 10.0:1 (20 min); 4.3:1 (1 h); 3.7:1 (2 h, full cosumption of PhIO).

(4) in absence of NaN_3:

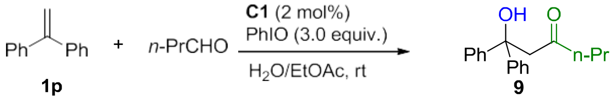

To a reaction mixture of catalyst C1 (2.6 mg, 0.004 mmol, 2 mol%) and PhIO (136.0 mg, 0.6 mmol, 3.0 equiv.) in a 10 mL-Schlenk tube were added H_2O (2.6 mL), n-butylaldehyde (88 uL, 1.0 mmol, 5.0 equiv.), and olefin 1p (35 uL, 0.2 mmol, 1.0 equiv) under a nitrogen atmosphere. Then 1.6 mL EtOAc was added to the above reaction mixture and the reaction was stirred for about 48 h at room temperature. After the completion of the reaction as judged by TLC analysis (the reaction generally completed when the solid PhIO disappeared), the reaction mixture

was extracted with EA (20.0 mL×1) and CH_2Cl_2 (15.0 mL×2). The combined organic layer was dried over anhydrous sodium sulfate and evaporated under reduced pressure to afford the crude product, which was purified by flash chromatography on silica gel to give the desired product **9** (22.8 mg, 41%). ^{1}H NMR (400 MHz, $CDCl_3$): δ 7.35-7.40 (m, 4H), 7.26-7.32 (m, 4H), 7.17-7.23 (m, 2H), 3.38 (s, 2H), 2.39 (t, J = 7.2 Hz, 2H), 1.47–1.58 (m, 2H), 0.83 (t, J = 7.4 Hz, 3H); ^{13}C NMR (100 MHz, $CDCl_3$): δ 212.7, 146.2, 128.2, 126.9, 125.6, 77.04, 52.1, 46.5, 16.7, 13.5; HRMS (ESI+) exact mass calculated for $[M+Na]^+$ ($C_{18}H_{20}NaO_2$) requires m/z 291.1361, found m/z 291.1357.

(5) in the presence of a large excess of competitive nucleophiles:

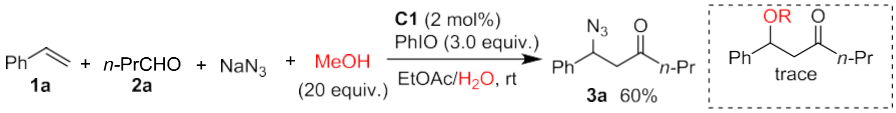

To a reaction mixture of catalyst **C1** (3.5 mg, 0.006 mmol, 2 mol%) and PhIO (198.0 mg, 0.9 mmol, 3.0 equiv.) in a 25 mL-Schlenk tube were added a aqueous solution of NaN_3 (4.0 mL, 0.3 M, 1.2 mmol, 4.0 equiv.), *n*-butylaldehyde (132 uL, 1.5 mmol, 5.0 equiv.), methanol (240.0 uL, 6.0 mmol, 20.0 equiv.) and olefin **1a** (35.0 uL, 0.3 mmol, 1.0 equiv.) under a nitrogen atmosphere. Then 2.4 mL EtOAc was added to the above reaction mixture and the reaction was stirred at room temperature. After the completion of the reaction as judged by TLC analysis, the reaction mixture was extracted with EA (20.0 mL×1) and CH_2Cl_2 (15.0 mL×2). The combined organic layer was dried over anhydrous sodium sulfate and evaporated under reduced pressure to afford the crude product, which was purified by flash chromatography on silica gel to give the desired products **3a** (39.1 mg, 0.18 mmol, 60%).

(6) the observation of the competition between two radical rebound approaches:

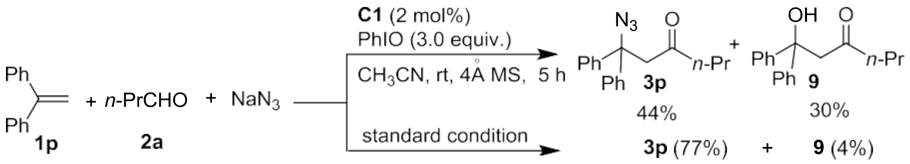

To a reaction mixture of catalyst **C1** (3.5 mg, 0.006 mmol, 2 mol%), NaN_3 (78.1mg, 1.2 mmol, 4.0 equiv.), 4Å MS (61.1 mg) and PhIO (198.0 mg, 0.9 mmol, 3.0 equiv.) in a 25 mL-Schlenk tube were added 4.0 mL CH_3CN under a nitrogen atmosphere. Then *n*-butylaldehyde (132.0 uL, 1.5 mmol, 5.0 equiv.) and olefin **1p** (53.0 uL, 0.3mmol, 1.0 equiv.) was added to the above reaction mixture and the reaction was stirred at room temperature. After the completion of the reaction as judged by TLC analysis, the reaction mixture was evaporated under reduced pressure to afford the crude product, which was purified by flash chromatography on silica gel to give the desired products **3p** (39.5 mg, 0.13 mmol, 44%) and **9** (24.2 mg, 0.09 mmol, 30%).

(7) Evaluation of factors affecting diastereoselectivity:

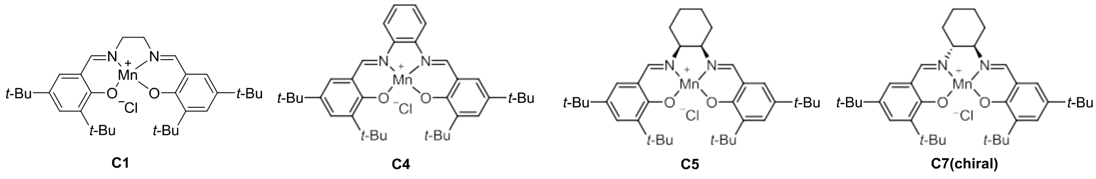

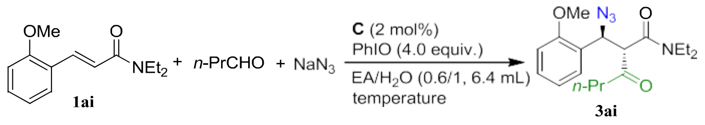

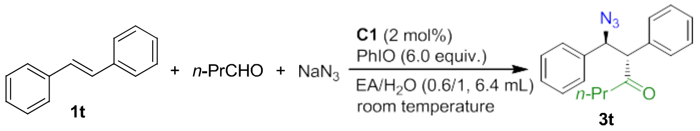

General experimental procedure:

To a reaction mixture of catalyst **C** (2 mol%) and a fine powder PhIO in a 25 mL-Schlenk tube were added a aqueous solution of NaN_3 (4.0 mL, 0.3 M, 1.2 mmol, 4.0 equiv.), freshly distilled aldehyde (1.5 mmol, 5.0 equiv.), and olefin (0.3 mmol) under a nitrogen atmosphere. Then 2.4 mL EtOAc was added to the above reaction mixture and the reaction was stirred at room temperature. After the completion of the reaction as judged by TLC analysis (the reaction generally completed when the solid PhIO disappeared), the reaction mixture was extracted with EA (20.0 mL×1) and CH_2Cl_2 (15.0 mL×2). The combined organic layer was dried over anhydrous sodium sulfate and evaporated under reduced pressure to afford the mixture for ^{1}HNMR analysis to determine diastereoselectivity.

Standard reaction condition: catalyst **C1**, room temperature, product **3ai**, dr 5:1.

Experiment 1: catalyst **C1**, heating at 60 ^{o}C: product **3ai**, dr 3:1.

Experiment 2: After standard reaction was complete, the reaction mixture was extracted with EA (20.0 mL×1) and CH_2Cl_2 (15.0 mL×2). The combined organic layer was dried over anhydrous sodium sulfate and evaporated under reduced pressure to afford the mixture, which was filtrated through a short silica gel column to give the crude products. Then the crude products were dissolved in EA/H_2O (0.6/1, 6.4 mL) and 20 mol% DIPEA (diisopropyl ethyl amine) was added to this mixture. The reaction was stirred at room temperature for 12 h, product **3ai**, dr 4:1.

Experiment 3: After standard reaction was complete, the reaction mixture was extracted with EA (20.0 mL×1) and CH_2Cl_2 (15.0 mL×2). The combined organic layer was dried over anhydrous sodium sulfate and evaporated under reduced pressure to afford the crude products. The crude products were dissolved in EA/H_2O (0.6/1, 6.4 mL) and 20 mol% K_2CO_3 was added to this mixture. The reaction was stirred at room temperature for 12 h, product **3ai**, dr 3:1.

Experiment 4: using 2 mol% **C4** instead of **C1:** product **3ai**, dr 2.5:1.

Experiment 5: using 2 mol% **C5** instead of **C1:** product **3ai**, dr 4:1.

Experiment 6: using 2 mol% **C7** (epimer of **C5, chiral**) instead of **C1:** product **3ai**, dr 3:1.

Experiment 7: using 2 mol% **C1**, room temperature, product **3t**, dr 5:1

Experiment 8: using 2 mol% **C4** instead of **C1,** room temperature, product **3t**, dr 3:1

X-ray crystallographic data

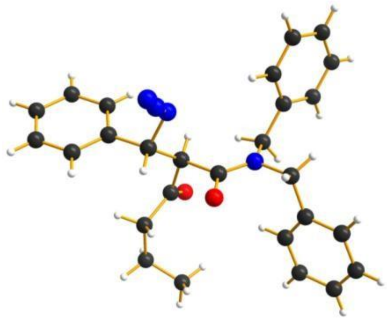

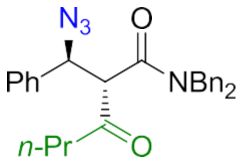

3aa

Table 1: Crystal data and structure refinement for 3aa

Empirical formula	$C_{27}H_{28}N_4O_2$
Formula weight	440.53
Temperature/K	298 (2)
Crystal system	monoclinic
Space group	C2/c
a/Å	22.679 (7)
b/Å	10.176 (3)
c/Å	21.562 (6)
α/°	90
β/°	94.879 (4)
γ/°	90
Volume/Å3	4958 (2)
Z	8
ρ_{calc}g/cm^3	1.180
μ/mm^{-1}	0.076
F(000)	1872.0
Crystal size/mm^3	0.100 × 0.040 × 0.020
Radiation	MoKα (λ = 0.71073)
2Θ range for data collection/°	3.604 to 49.99
Index ranges	-15 ≤ h ≤ 26, -12 ≤ k ≤ 12, -25 ≤ l ≤ 25
Reflections collected	14327
Independent reflections	4364 [R_{int} = 0.0386, R_{sigma} = 0.0419]
Data/restraints/parameters	4364/48/300
Goodness-of-fit on F^2	1.009
Final R indexes [I>=2σ (I)]	R_1 = 0.0444, wR_2 = 0.0939
Final R indexes [all data]	R_1 = 0.0967, wR_2 = 0.1222
Largest diff. peak/hole / e Å^{-3}	0.14/-0.15

Copies of NMR spectra

^{1}H NMR spectrum of compound **4b'** ($CDCl_3$, 400MHz)

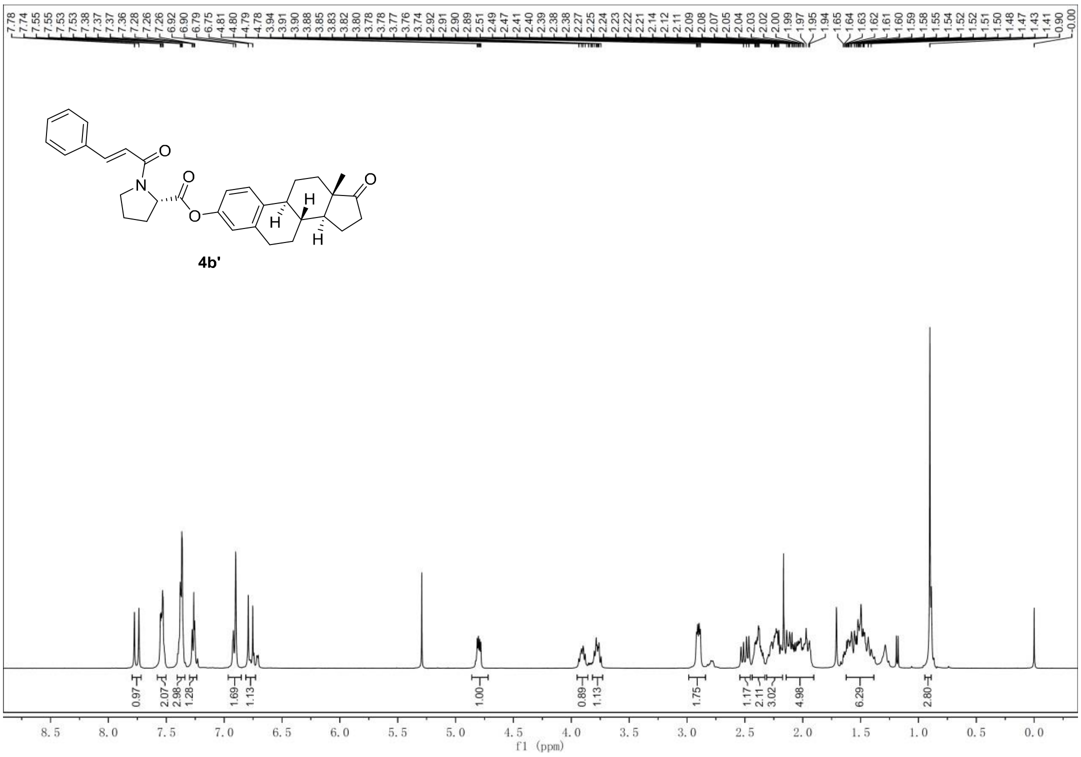

^{13}C NMR spectrum of compound **4b'** ($CDCl_3$, 100MHz)

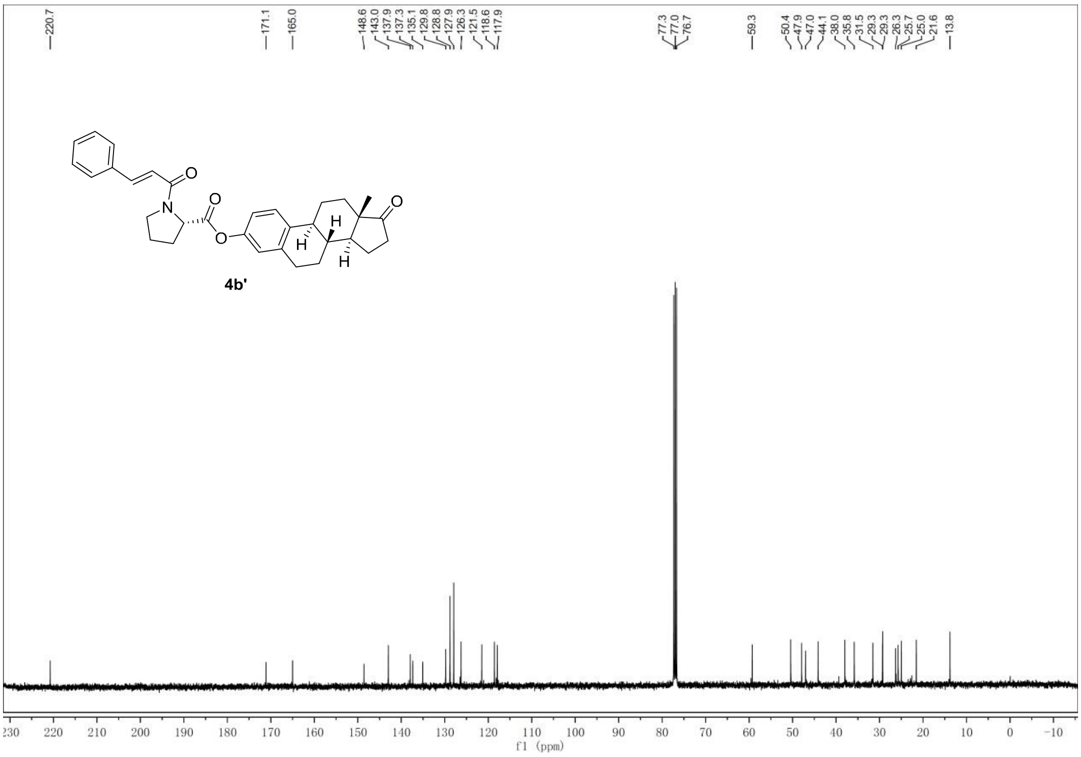

^{1}H NMR spectrum of compound **5a'** ($CDCl_3$, 400MHz)

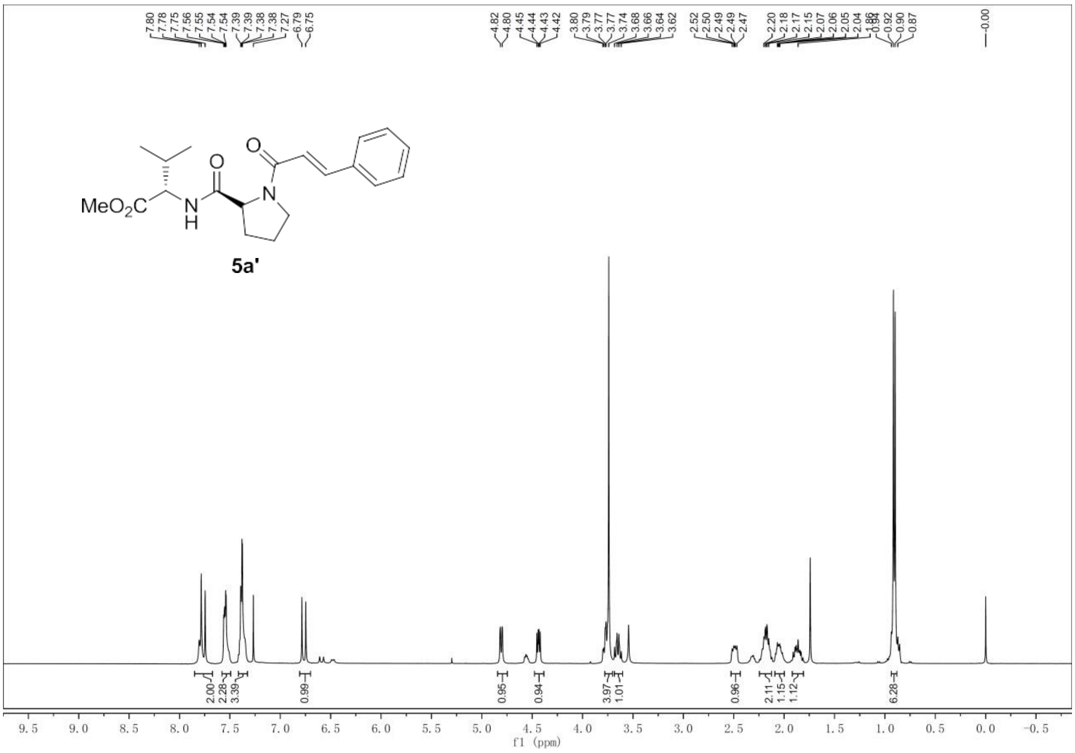

^{13}C NMR spectrum of compound **5a'** ($CDCl_3$, 100MHz)

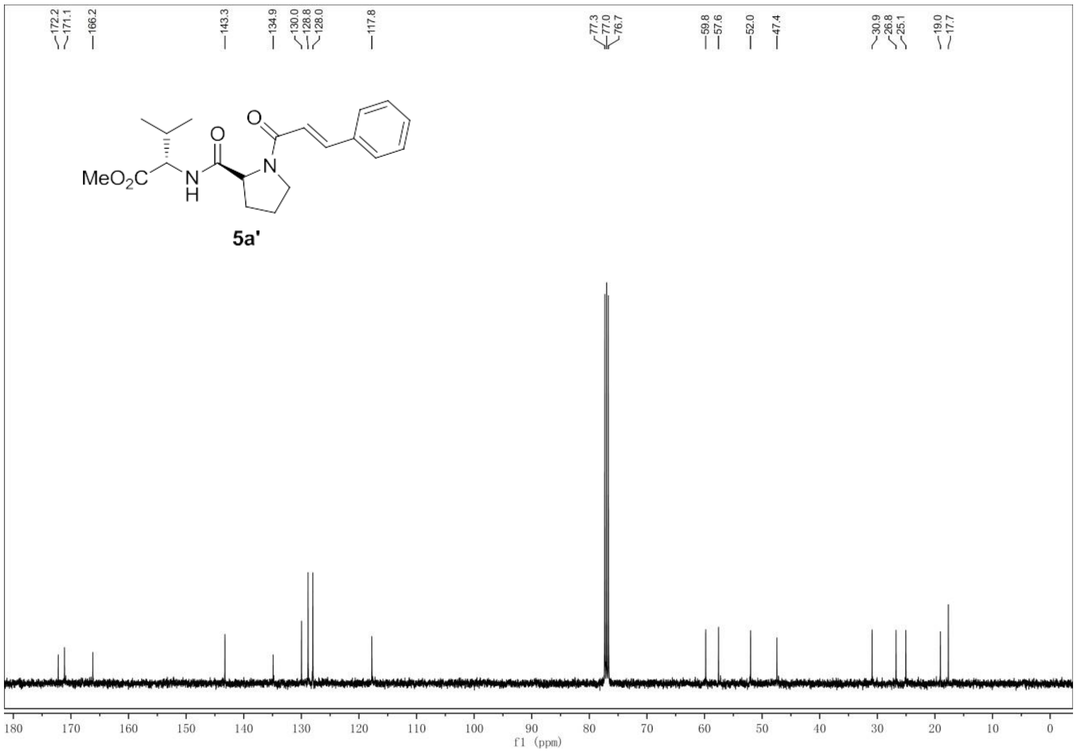

^{1}H NMR spectrum of compound **5b'** ($CDCl_3$, 400MHz)

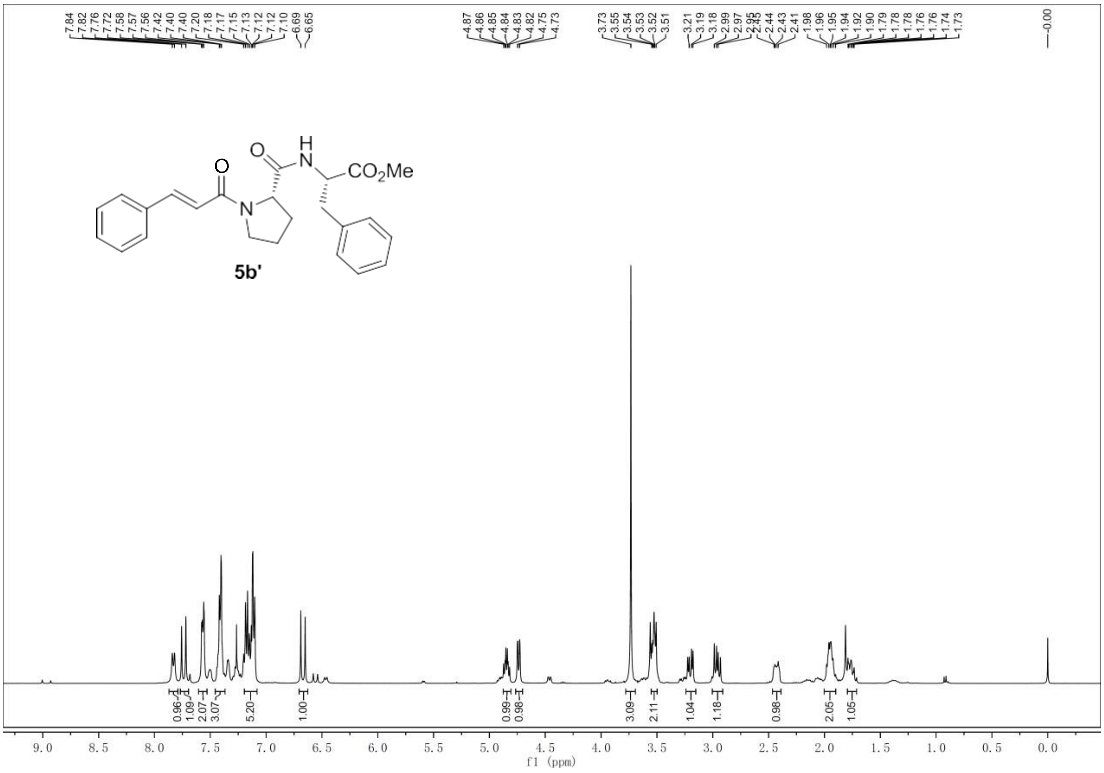

^{13}C NMR spectrum of compound **5b'** ($CDCl_3$, 100MHz)

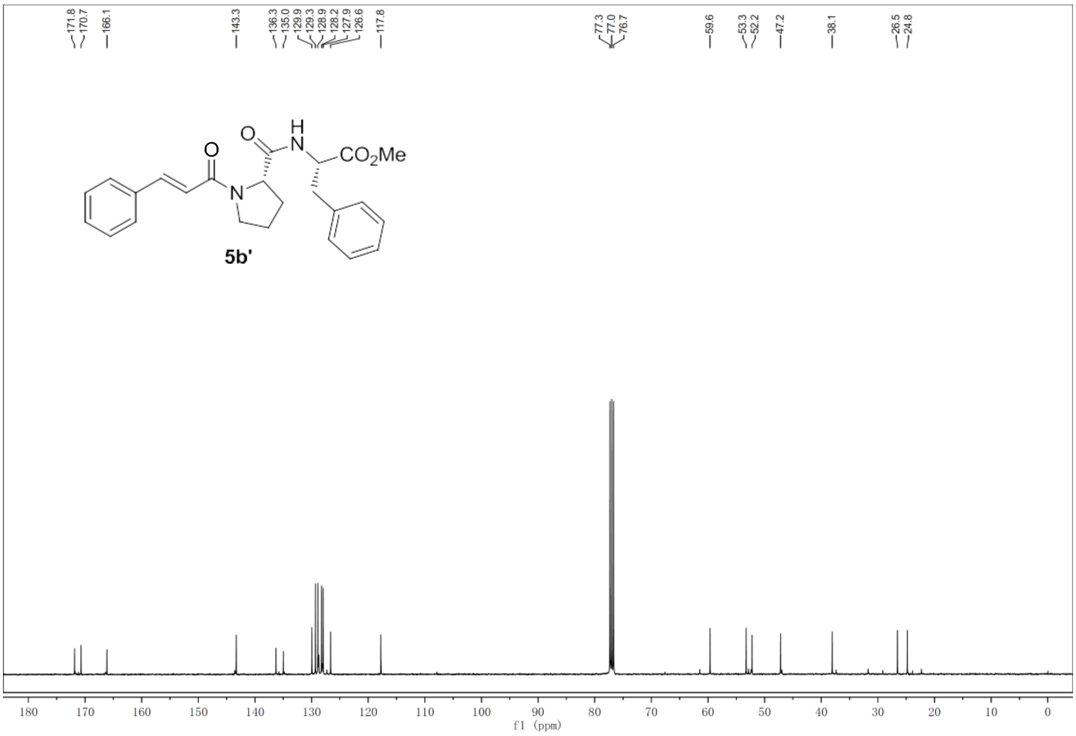

^{1}H NMR spectrum of compound **3a** ($CDCl_3$, 400MHz)

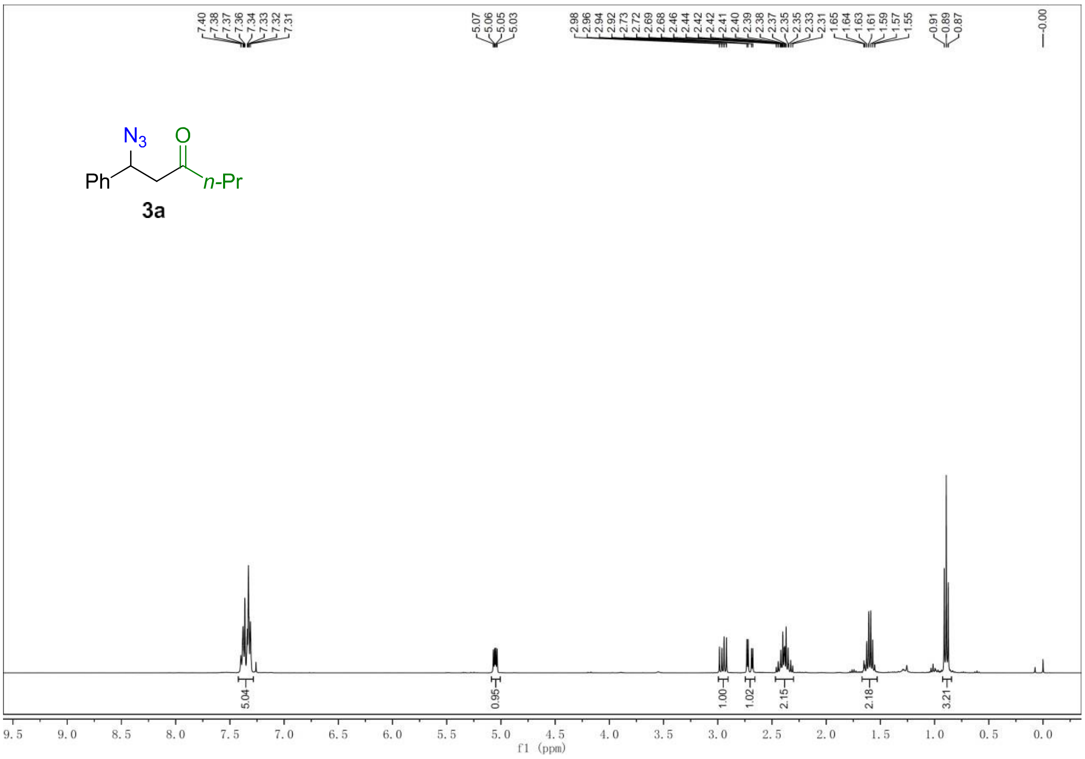

^{13}C NMR spectrum of compound **3a** ($CDCl_3$, 100MHz)

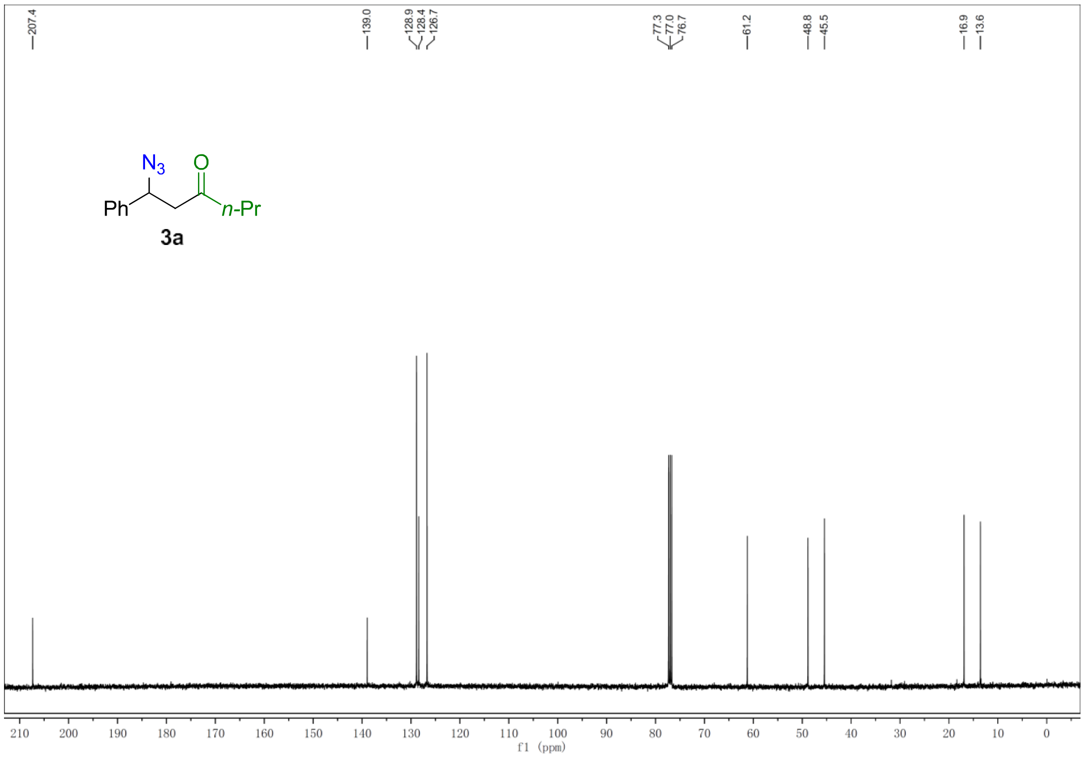

^{1}H NMR spectrum of compound **3b** ($CDCl_3$, 400MHz)

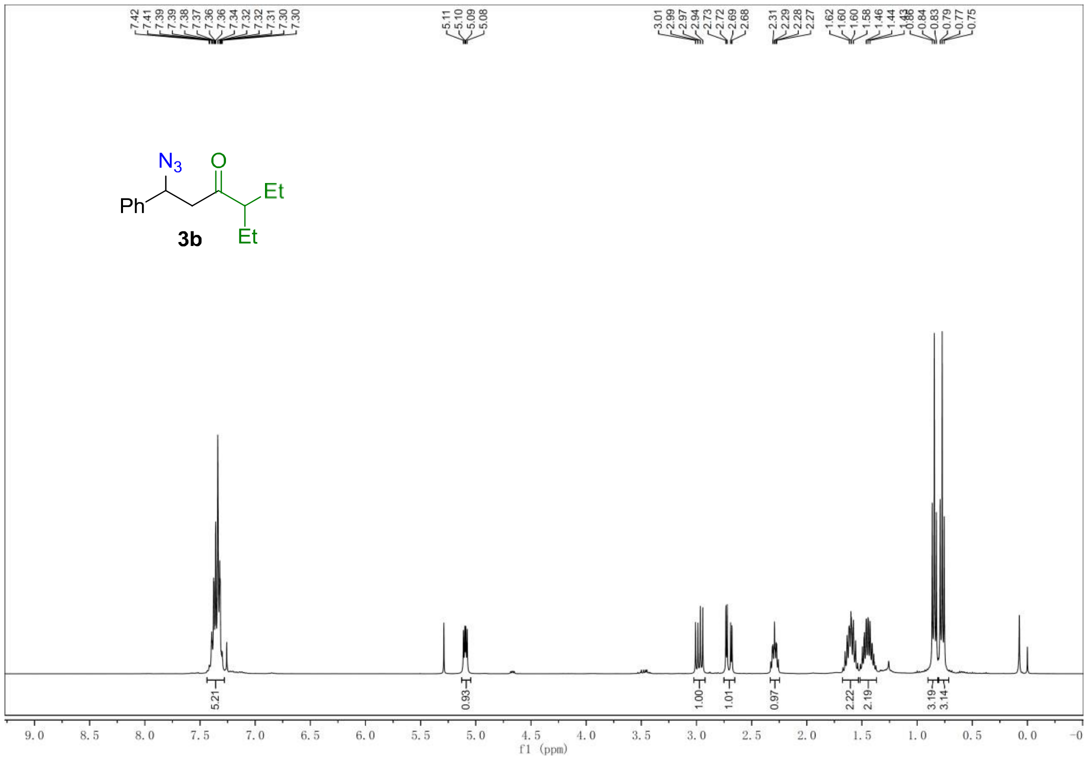

^{13}C NMR spectrum of compound **3b** ($CDCl_3$, 100MHz)

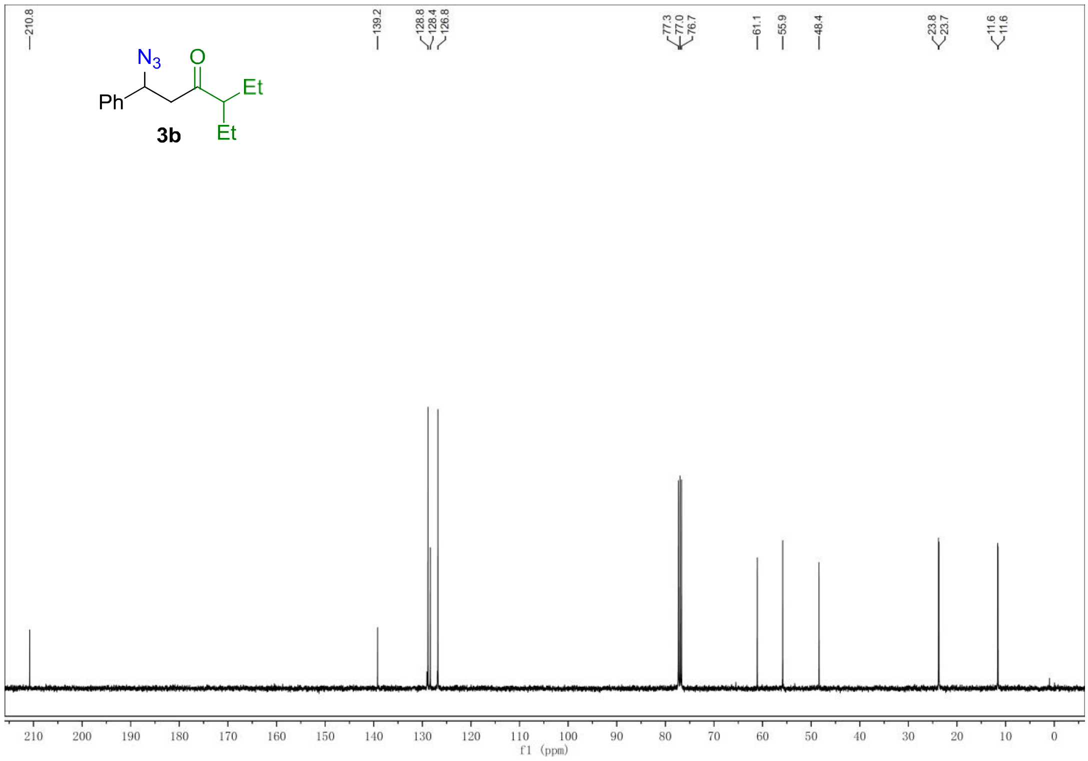

^{1}H NMR spectrum of compound **3c** ($CDCl_3$, 400MHz)

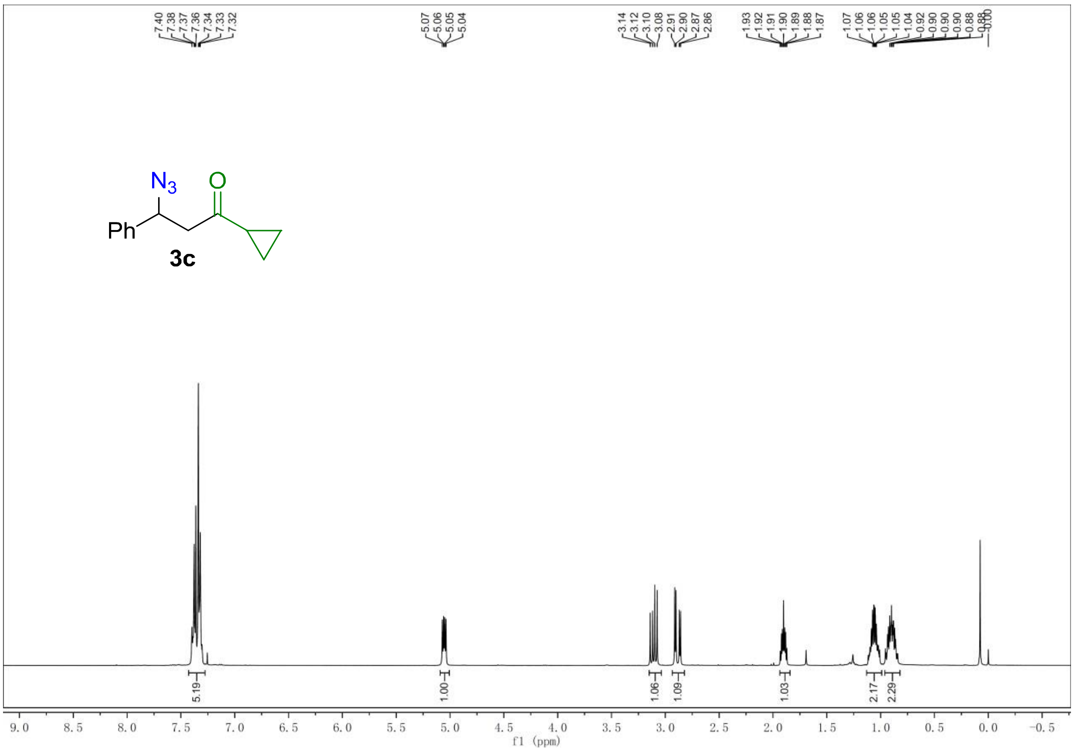

^{13}C NMR spectrum of compound **3c** ($CDCl_3$, 100MHz)

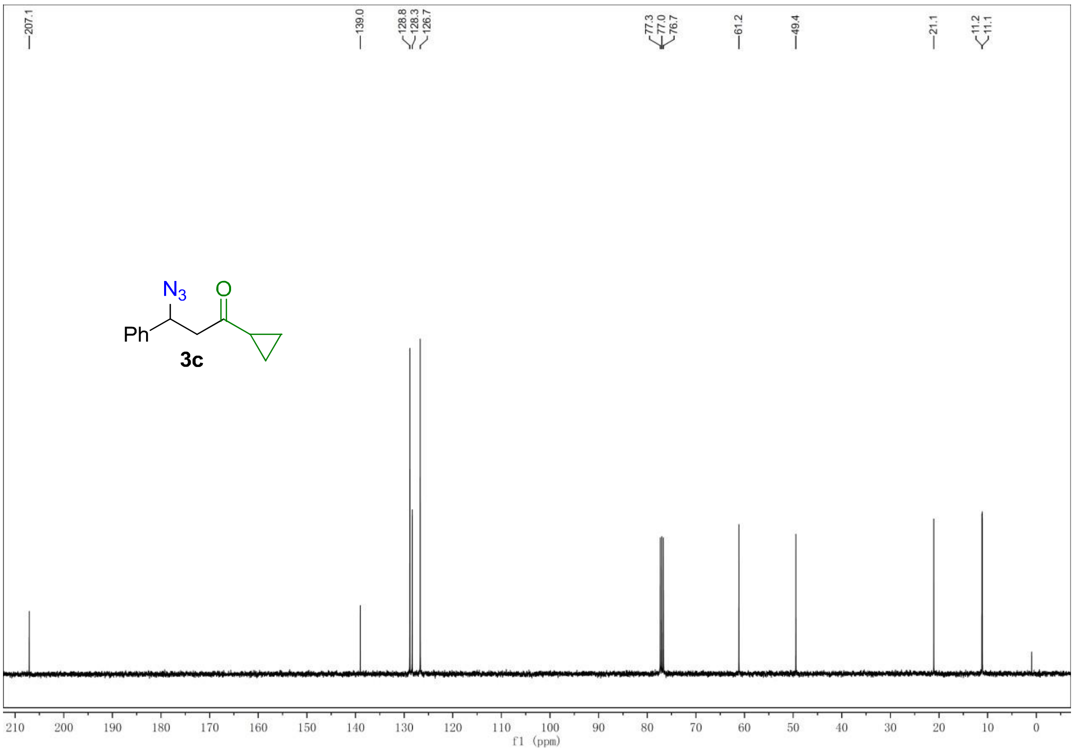

^{1}H NMR spectrum of compound **3d** ($CDCl_3$, 400MHz)

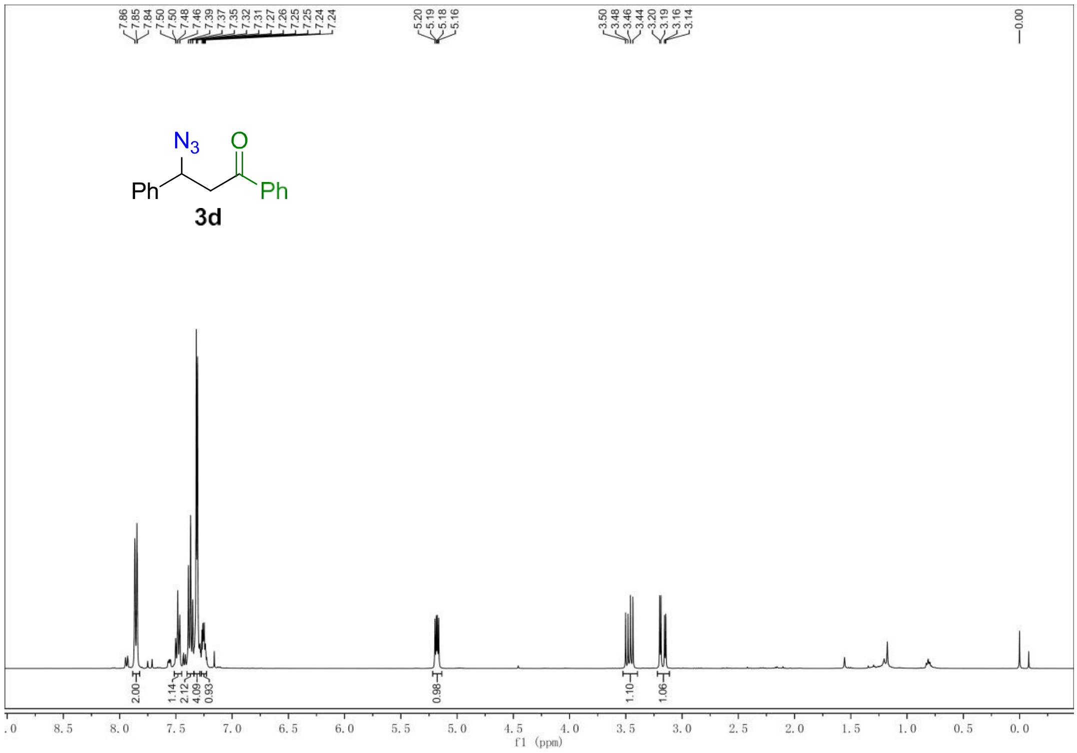

^{13}C NMR spectrum of compound **3d** ($CDCl_3$, 100MHz)

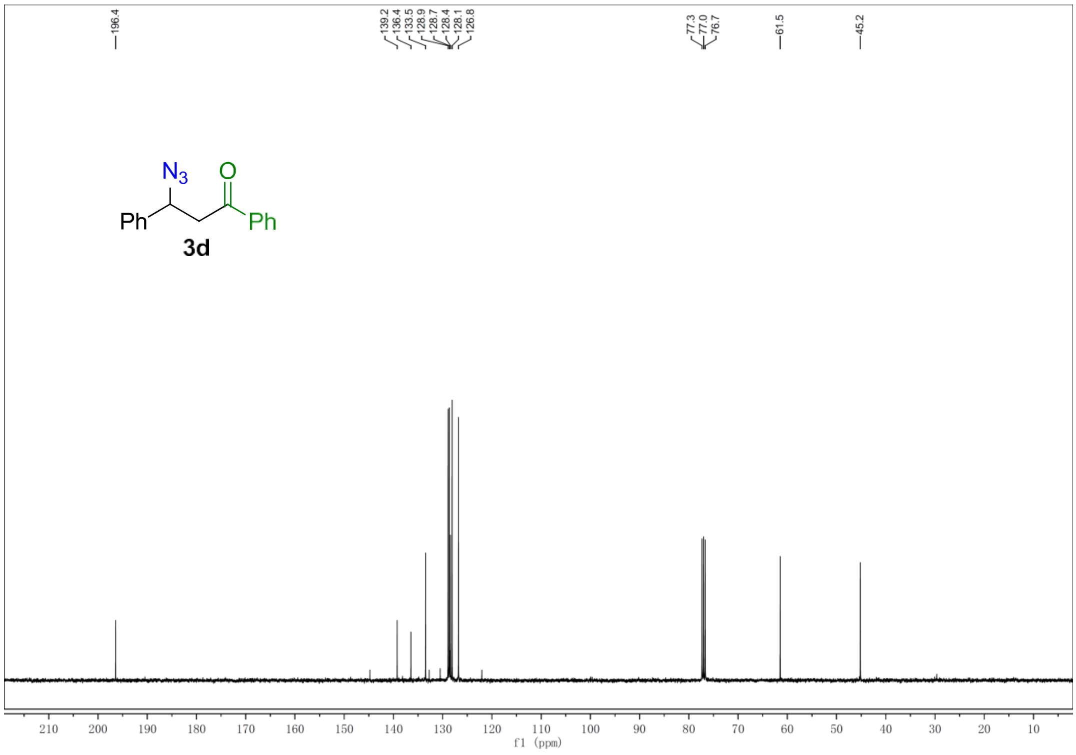

^{1}H NMR spectrum of compound **3e** ($CDCl_3$, 400MHz)

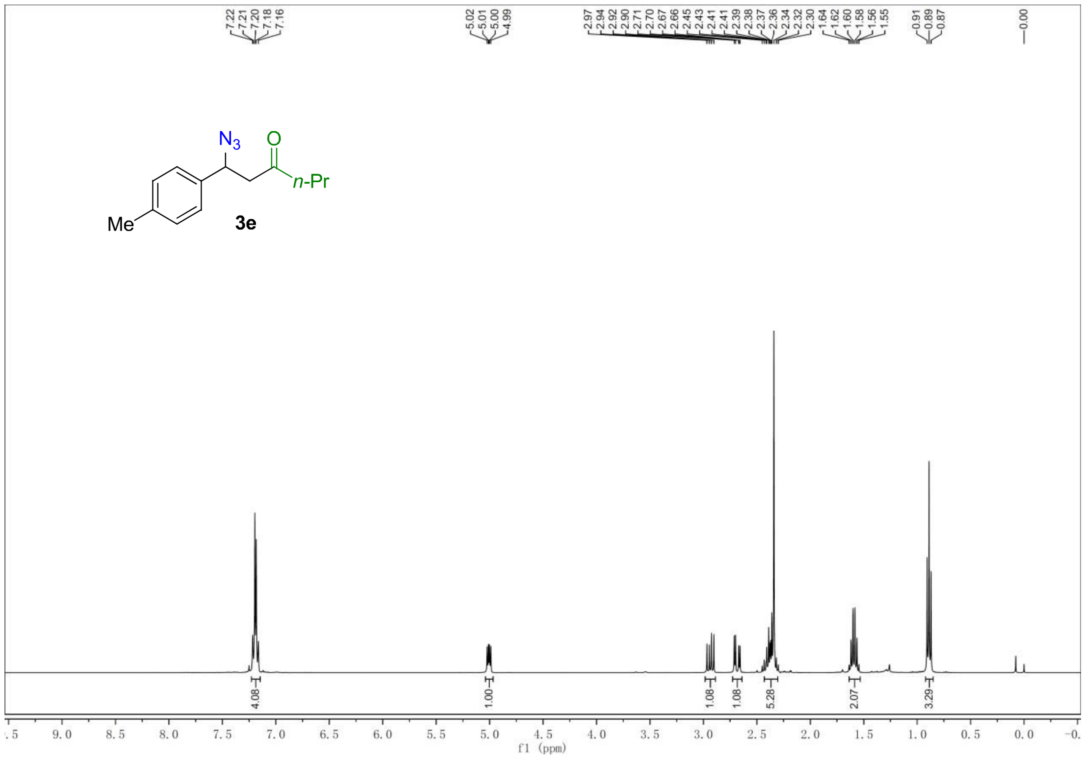

^{13}C NMR spectrum of compound **3e** ($CDCl_3$, 100MHz)

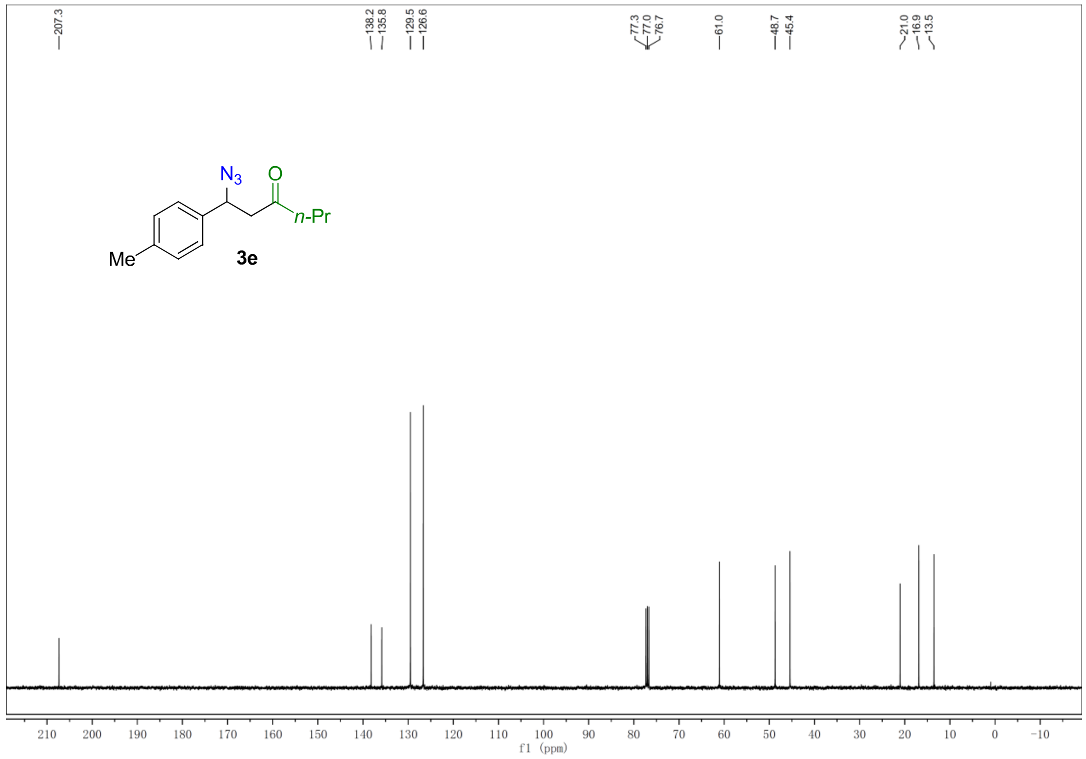

^{1}H NMR spectrum of compound **3f** ($CDCl_3$, 400MHz)

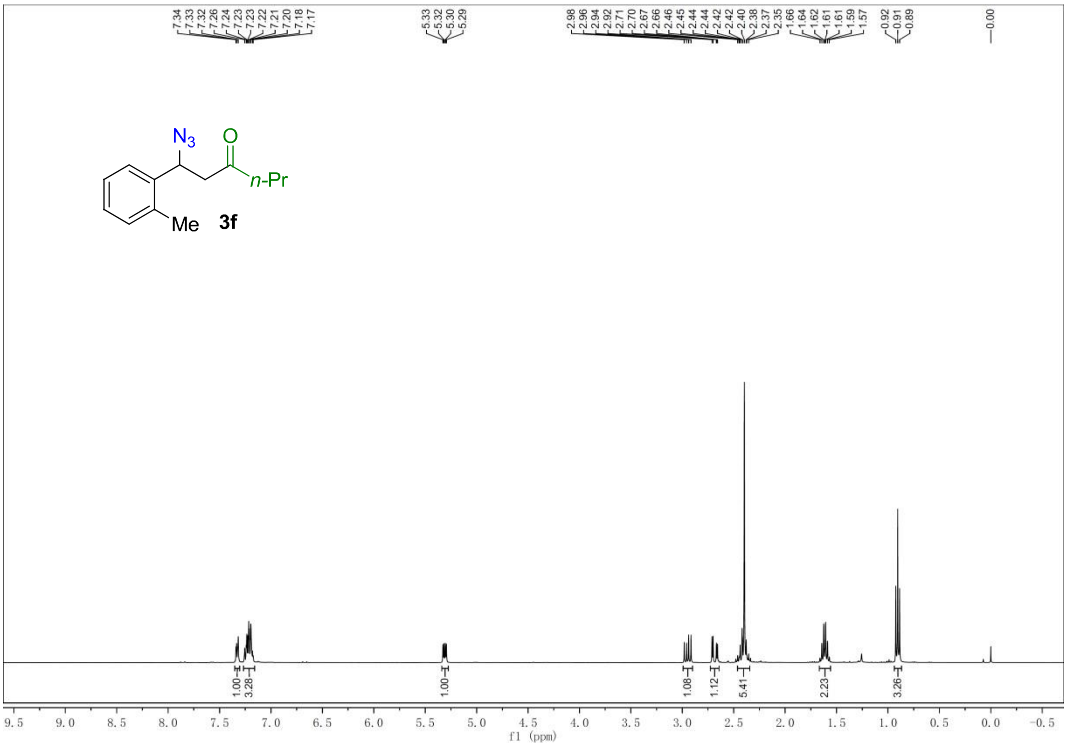

^{13}C NMR spectrum of compound **3f** ($CDCl_3$, 100MHz)

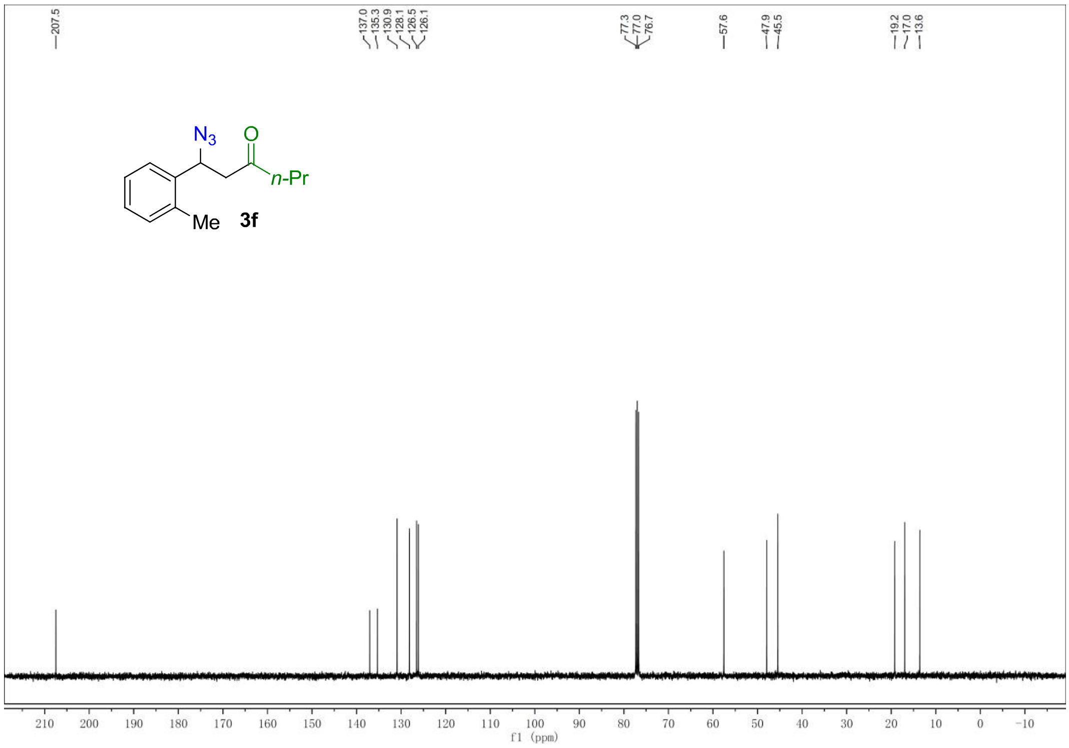

^{1}H NMR spectrum of compound **3g** ($CDCl_3$, 400MHz)

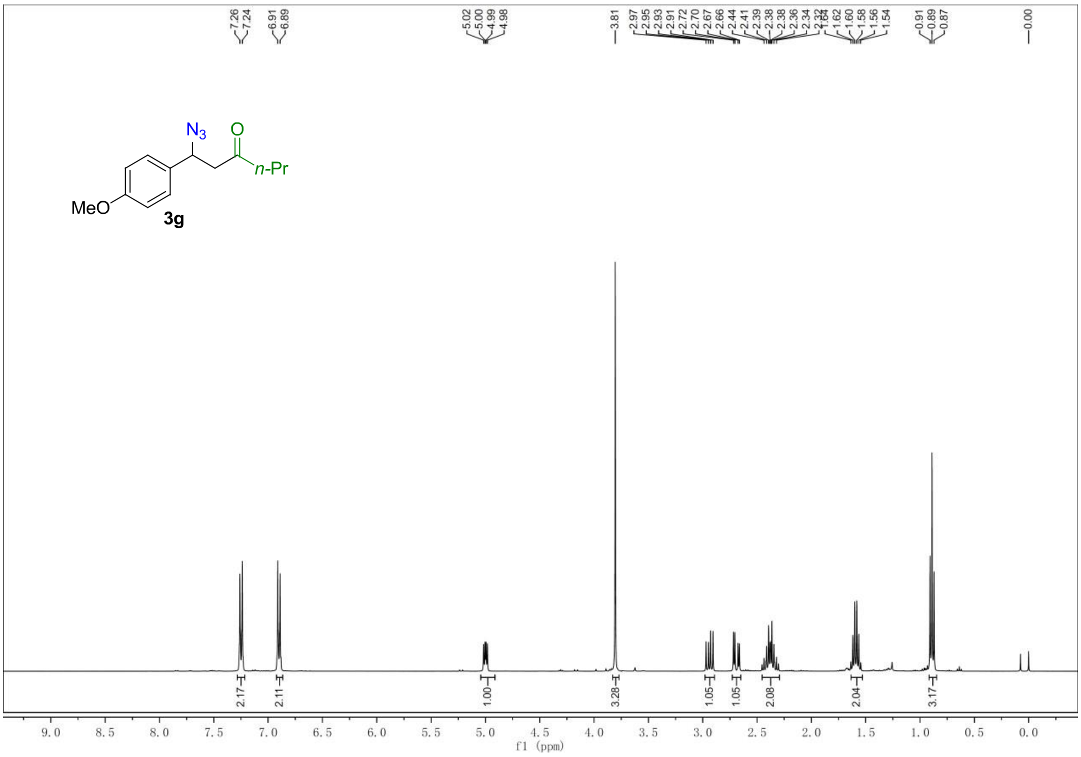

^{13}C NMR spectrum of compound **3g** ($CDCl_3$, 100MHz)

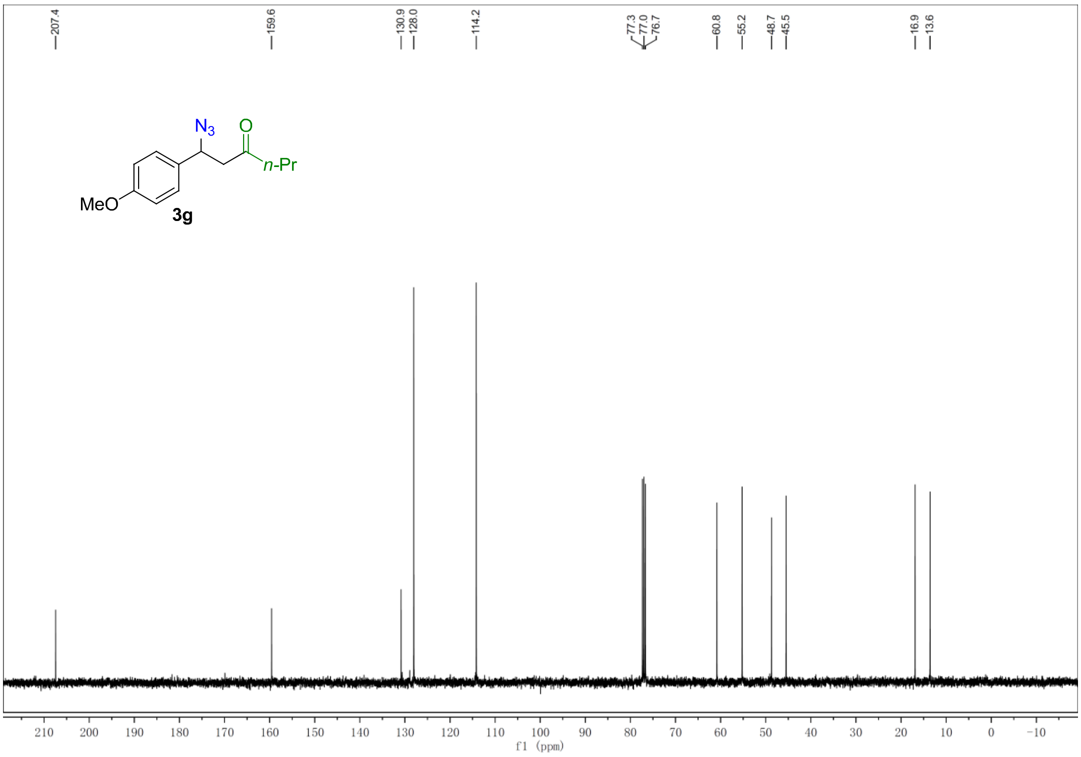

^{1}H NMR spectrum of compound **3h** ($CDCl_3$, 400MHz)

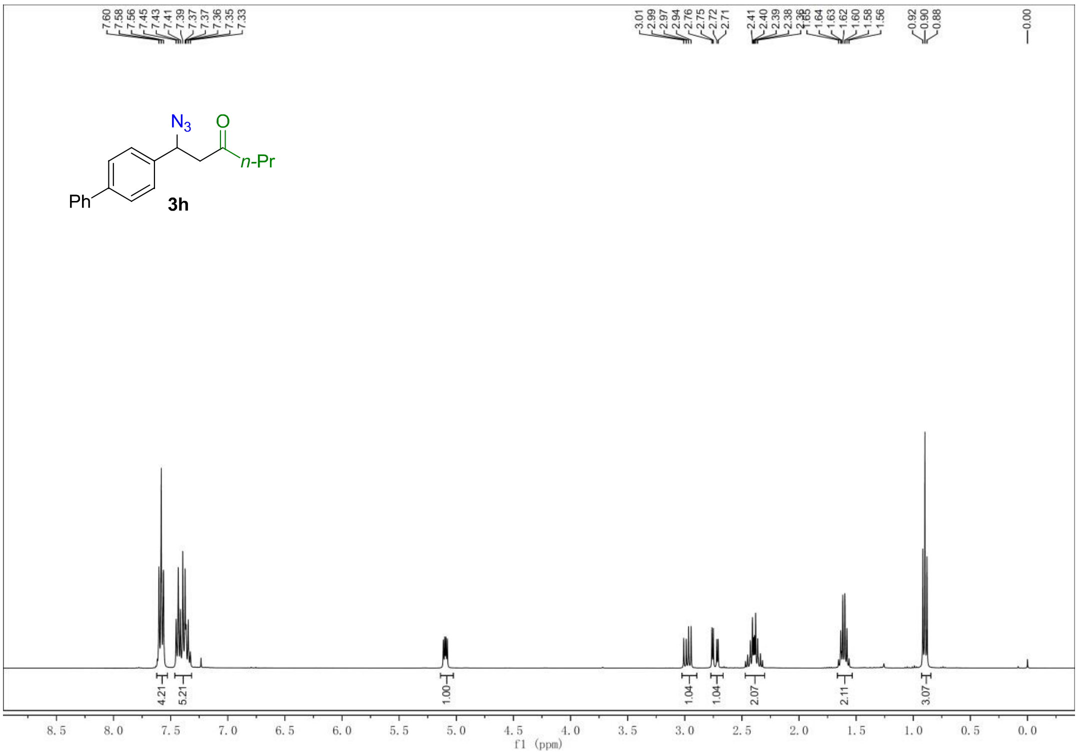

^{13}C NMR spectrum of compound **3h** ($CDCl_3$, 100MHz)

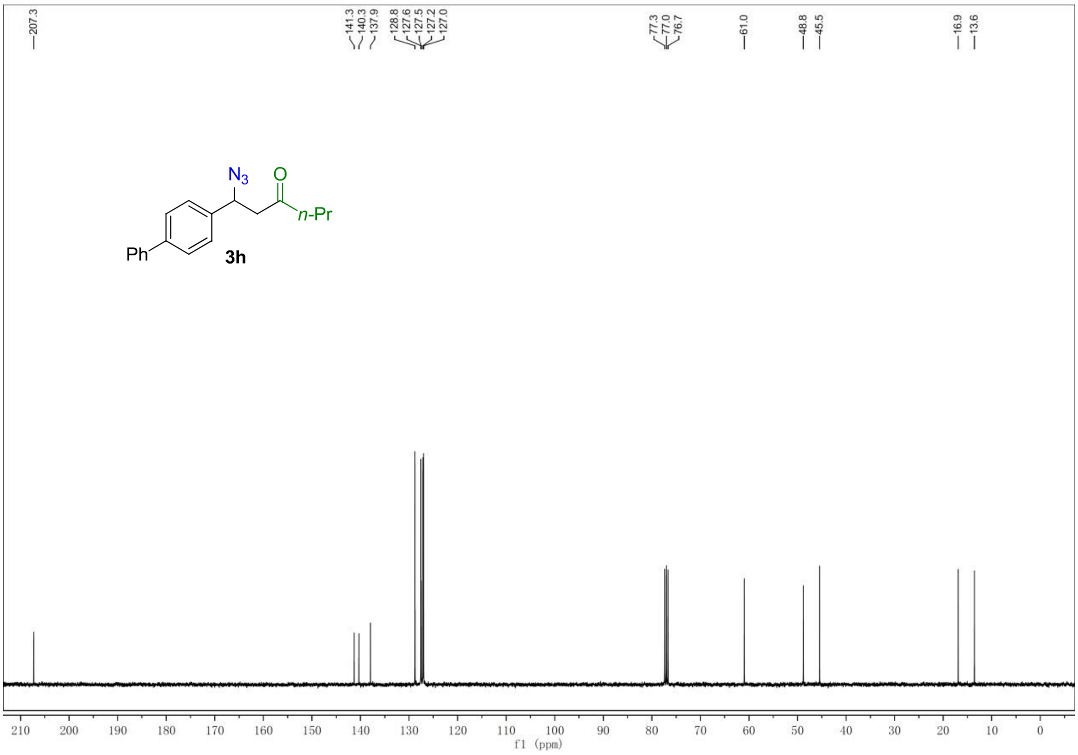

^{1}H NMR spectrum of compound **3i**($CDCl_3$, 400MHz)

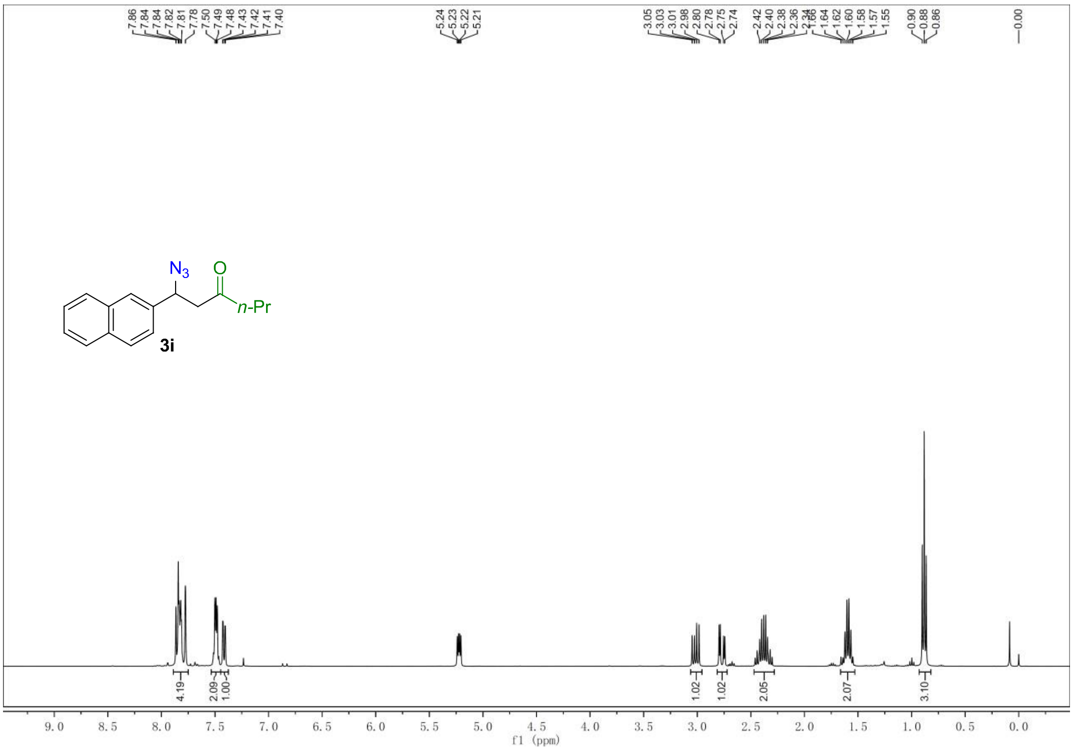

^{13}C NMR spectrum of compound **3i** ($CDCl_3$, 100MHz)

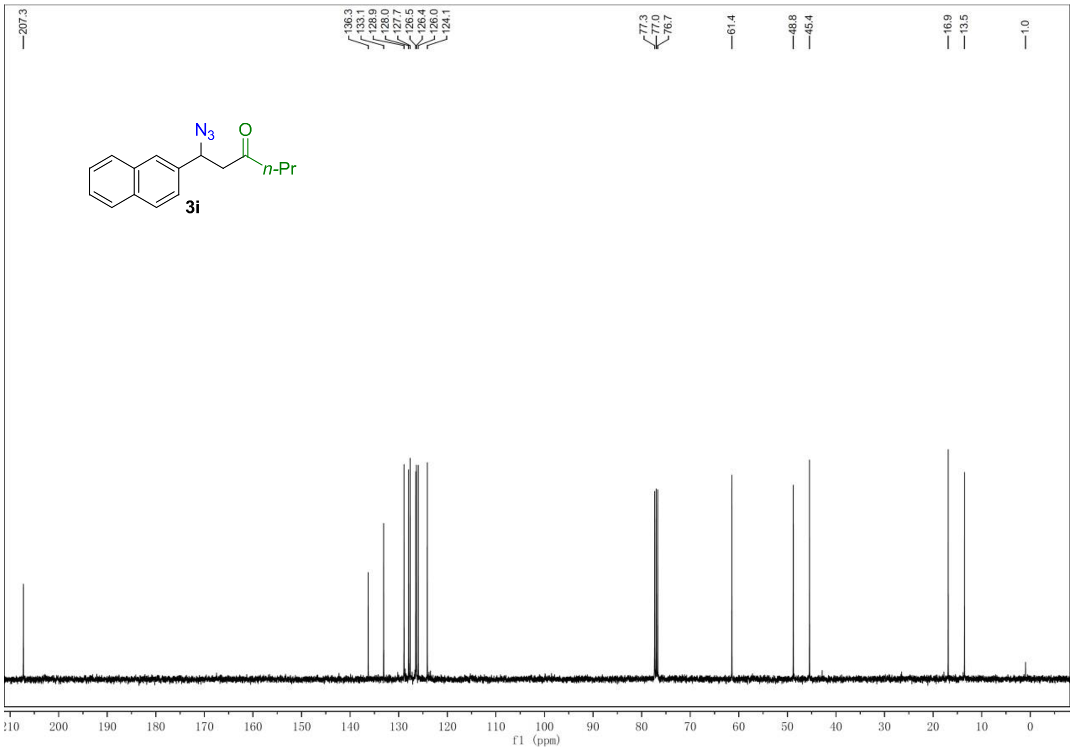

^{1}H NMR spectrum of compound **3j** ($CDCl_3$, 400MHz)

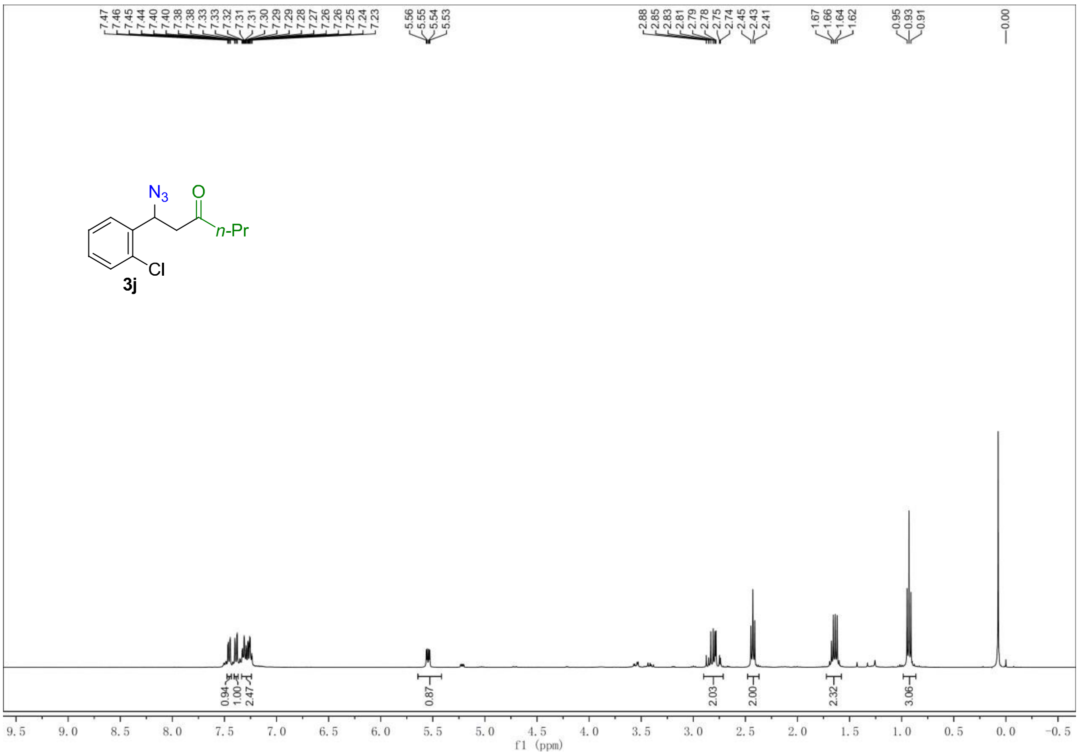

^{13}C NMR spectrum of compound **3j** ($CDCl_3$, 100MHz)

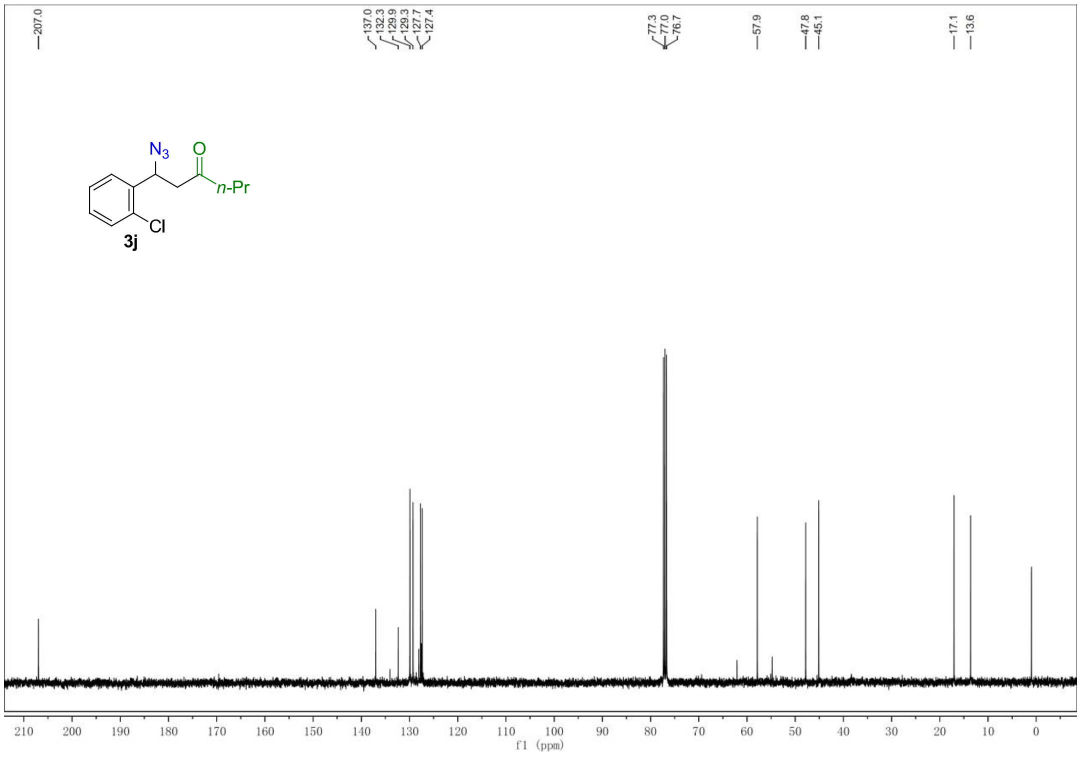

^{1}H NMR spectrum of compound **3k** ($CDCl_3$, 400MHz)

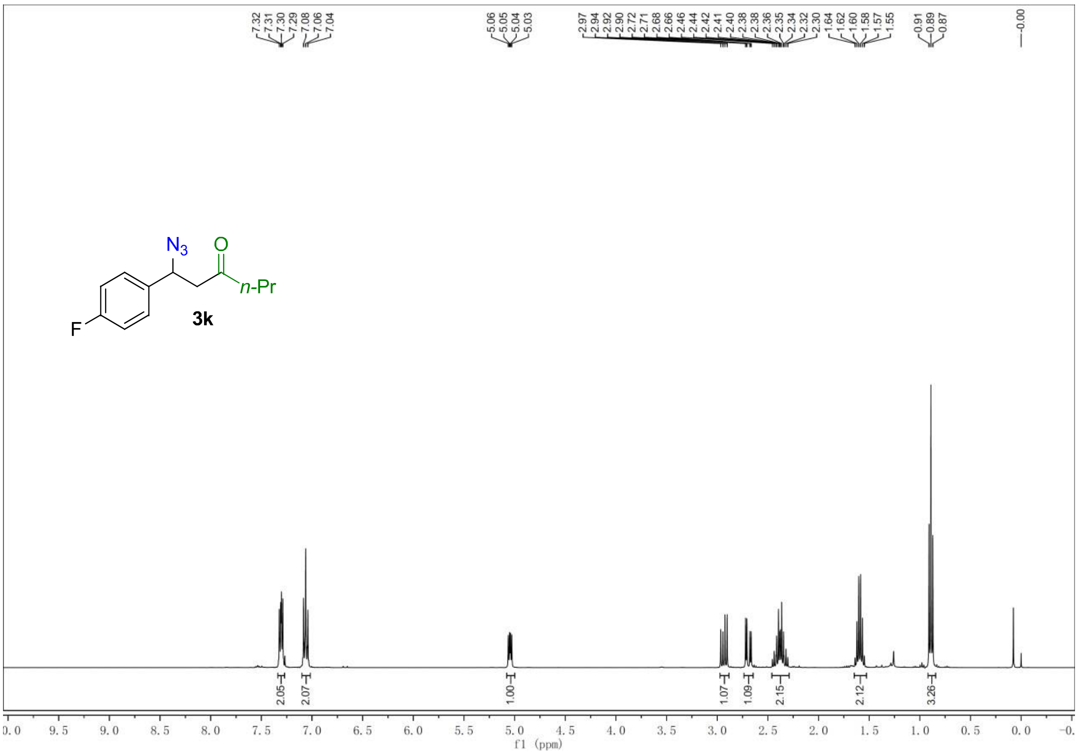

^{13}C NMR spectrum of compound **3k** ($CDCl_3$, 100MHz)

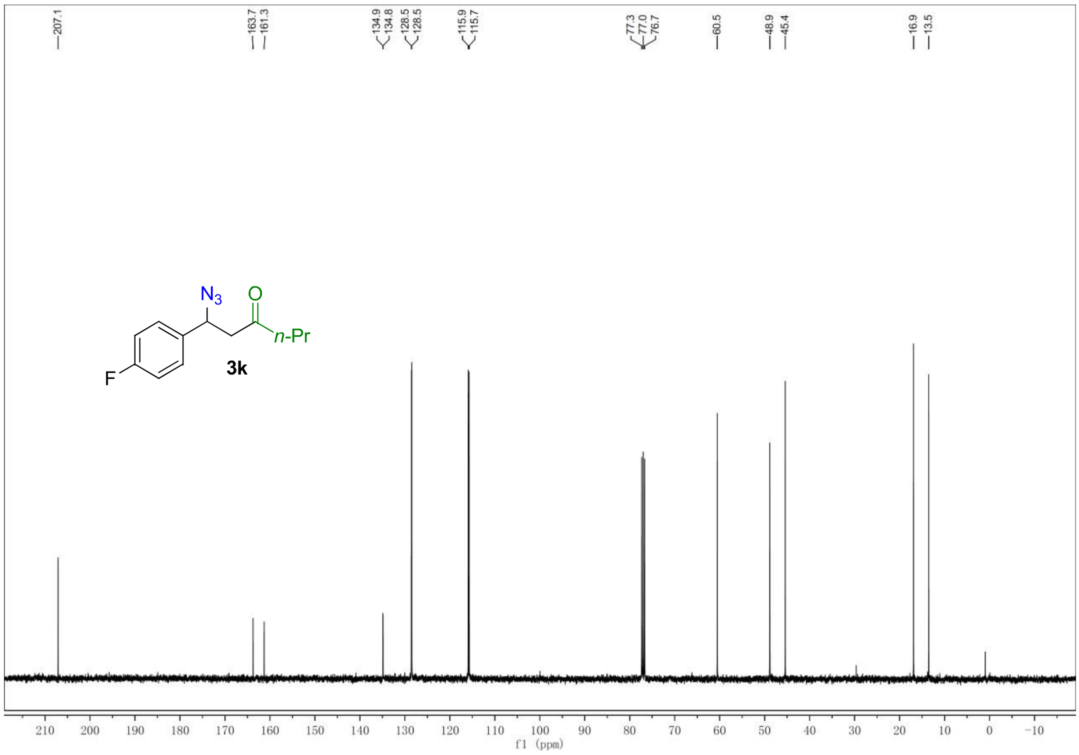

^{1}H NMR spectrum of compound **3l** ($CDCl_3$, 400MHz)

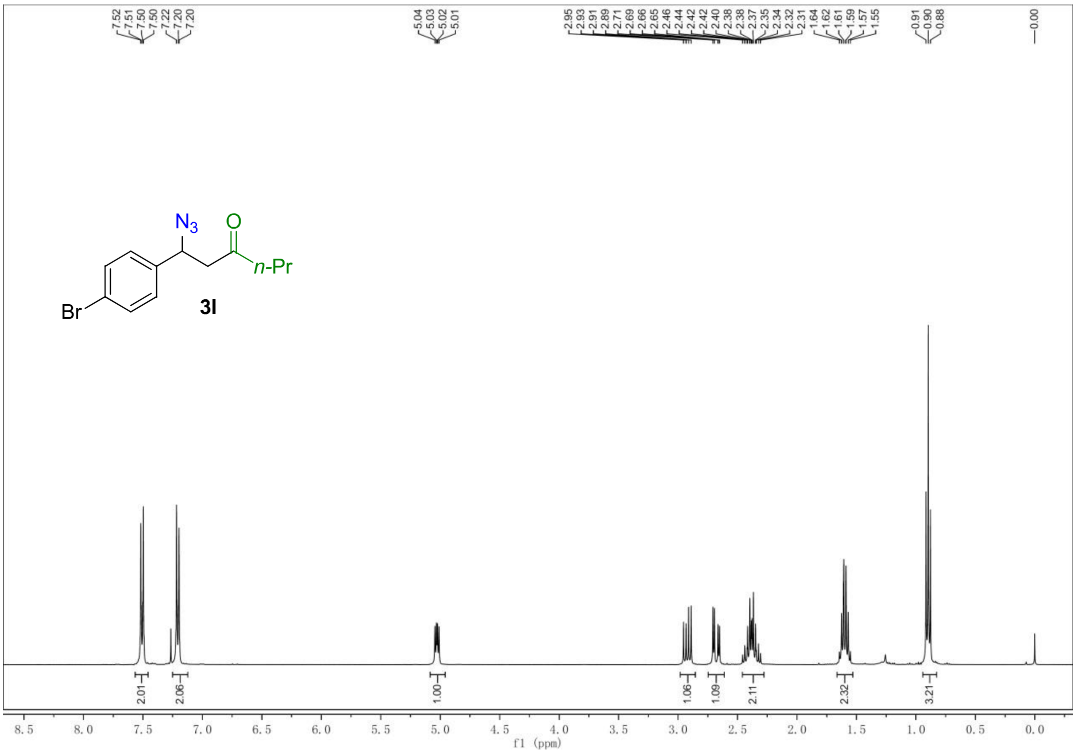

^{13}C NMR spectrum of compound **3l** ($CDCl_3$, 100MHz)

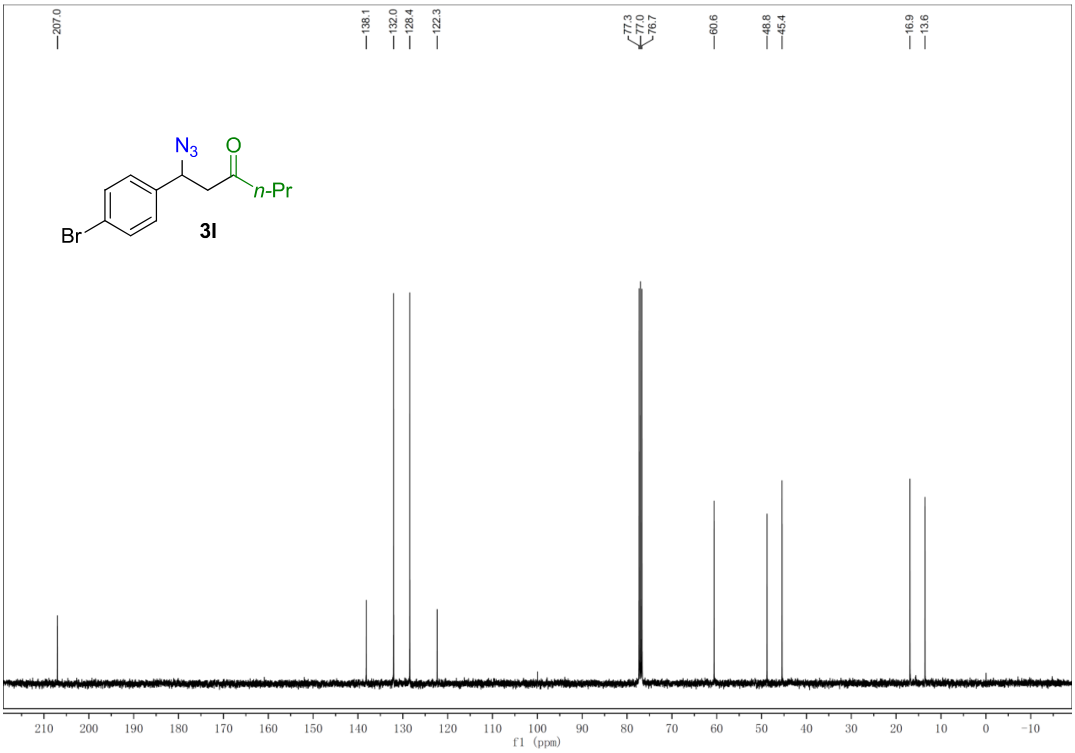

^{1}H NMR spectrum of compound **3m** ($CDCl_3$, 400MHz)

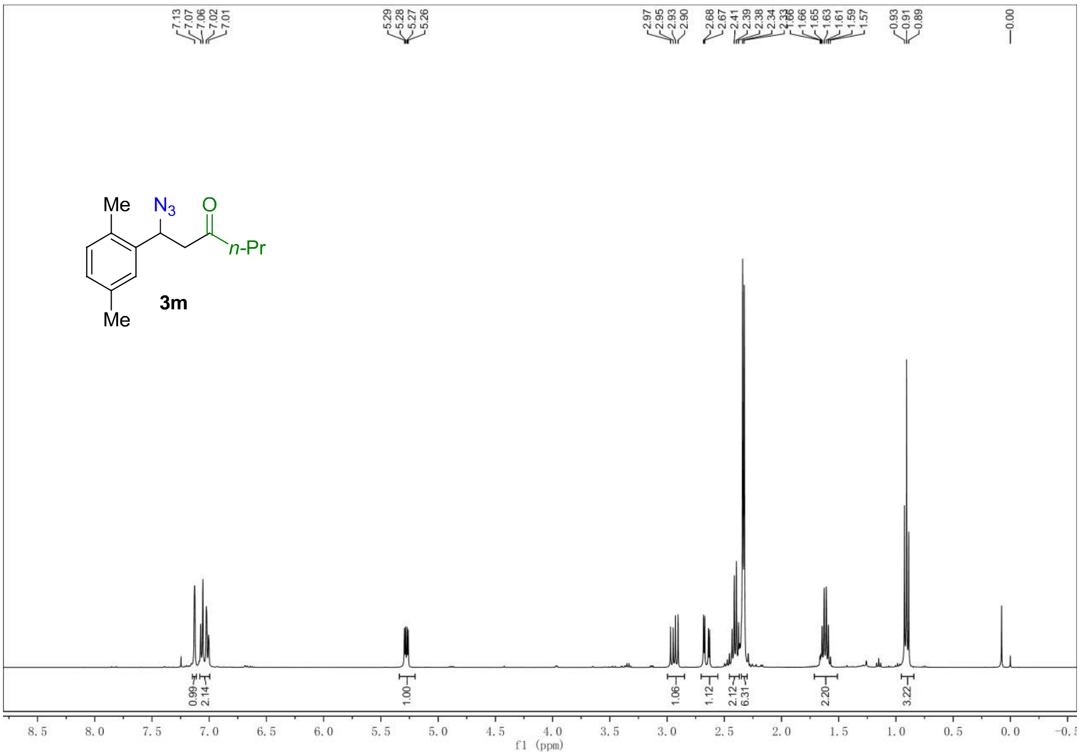

^{13}C NMR spectrum of compound **3m** ($CDCl_3$, 100MHz)

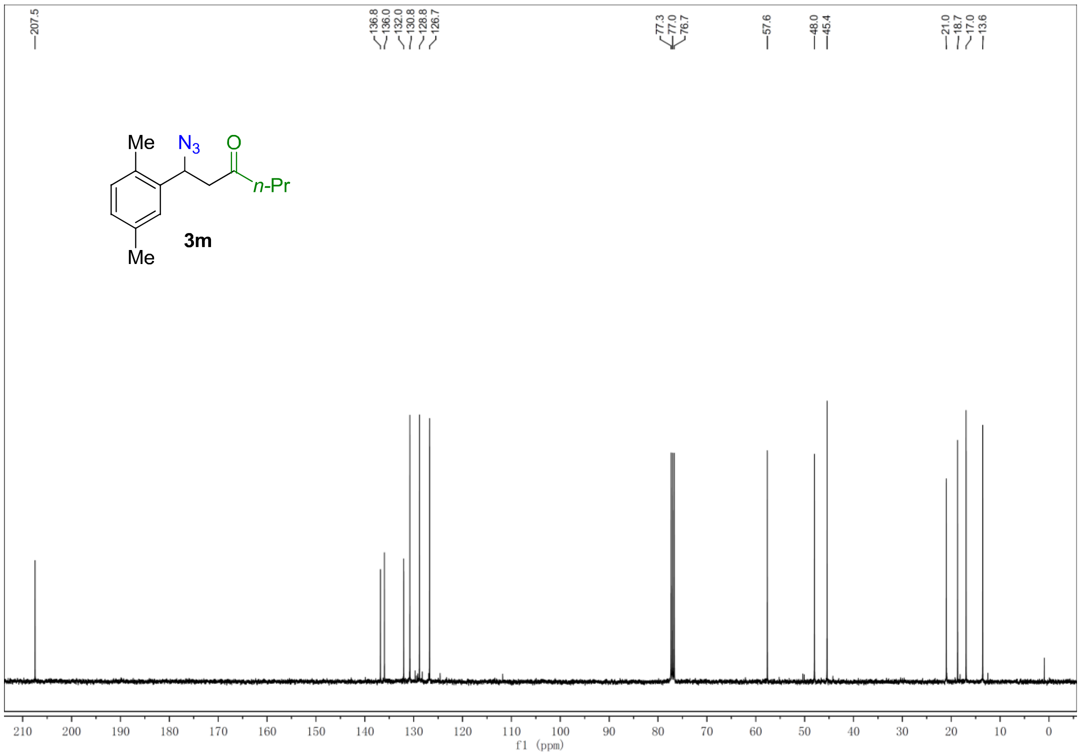

^1H NMR spectrum of compound **3n** ($CDCl_3$, 400MHz)

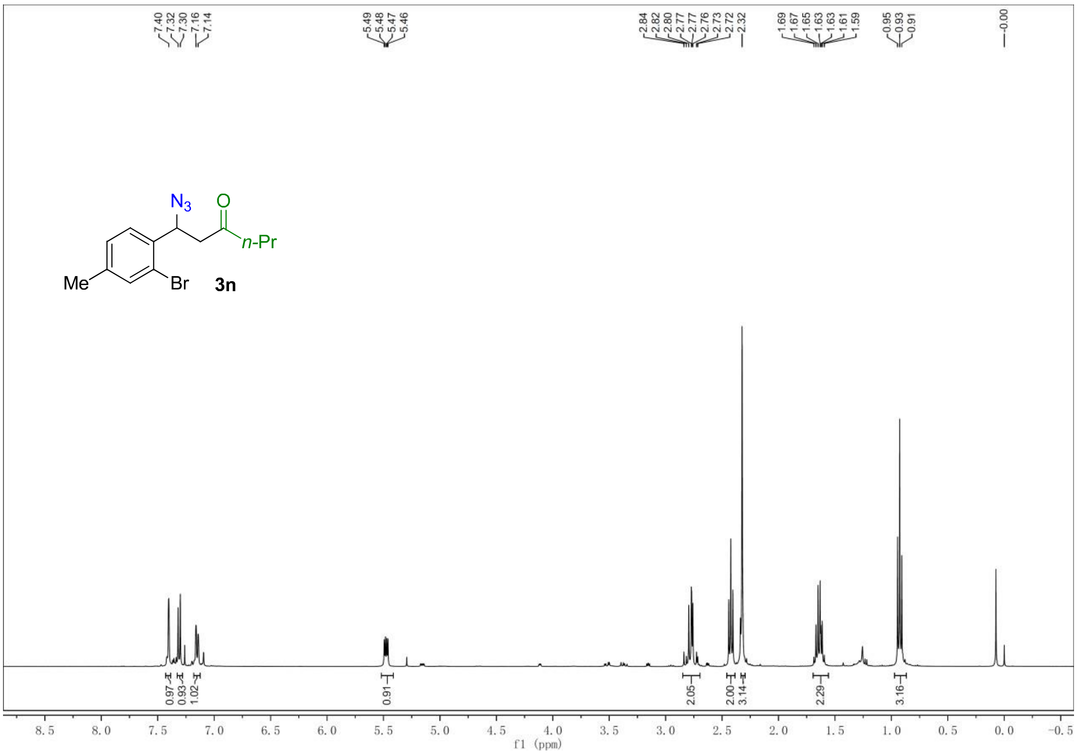

^{13}C NMR spectrum of compound **3n** ($CDCl_3$, 100MHz)

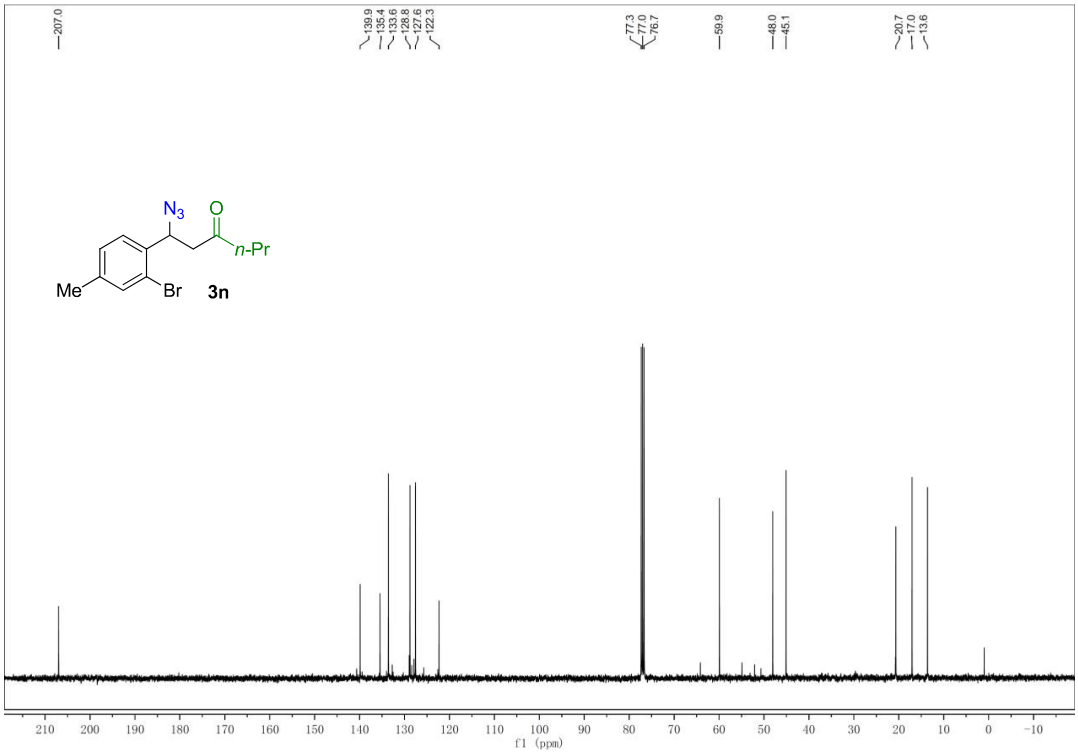

^{1}H NMR spectrum of compound **3o** ($CDCl_3$, 400MHz)

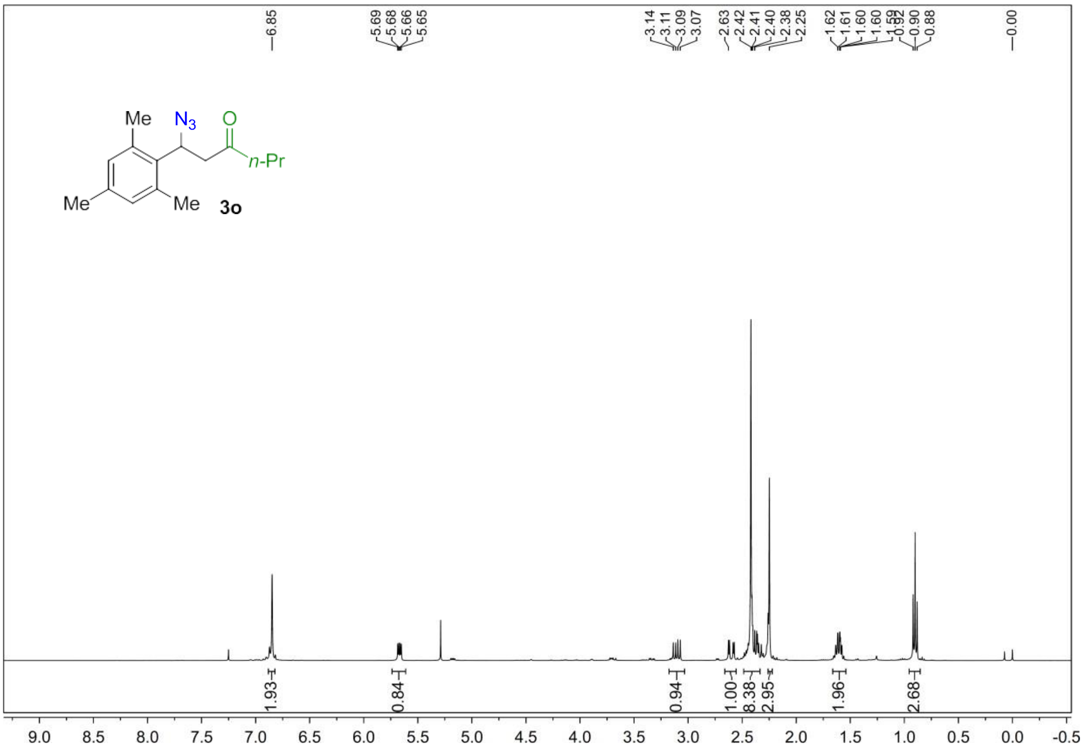

^{13}C NMR spectrum of compound **3o** ($CDCl_3$, 100MHz)

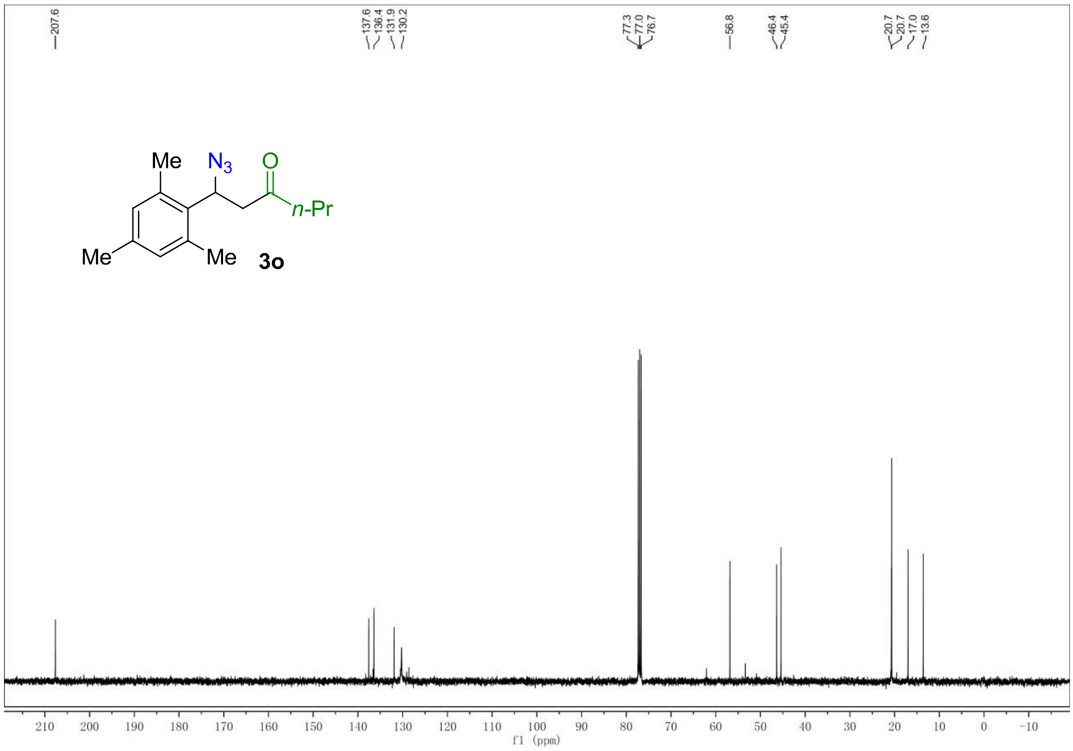

^{1}H NMR spectrum of compound **3p** ($CDCl_3$, 400MHz)

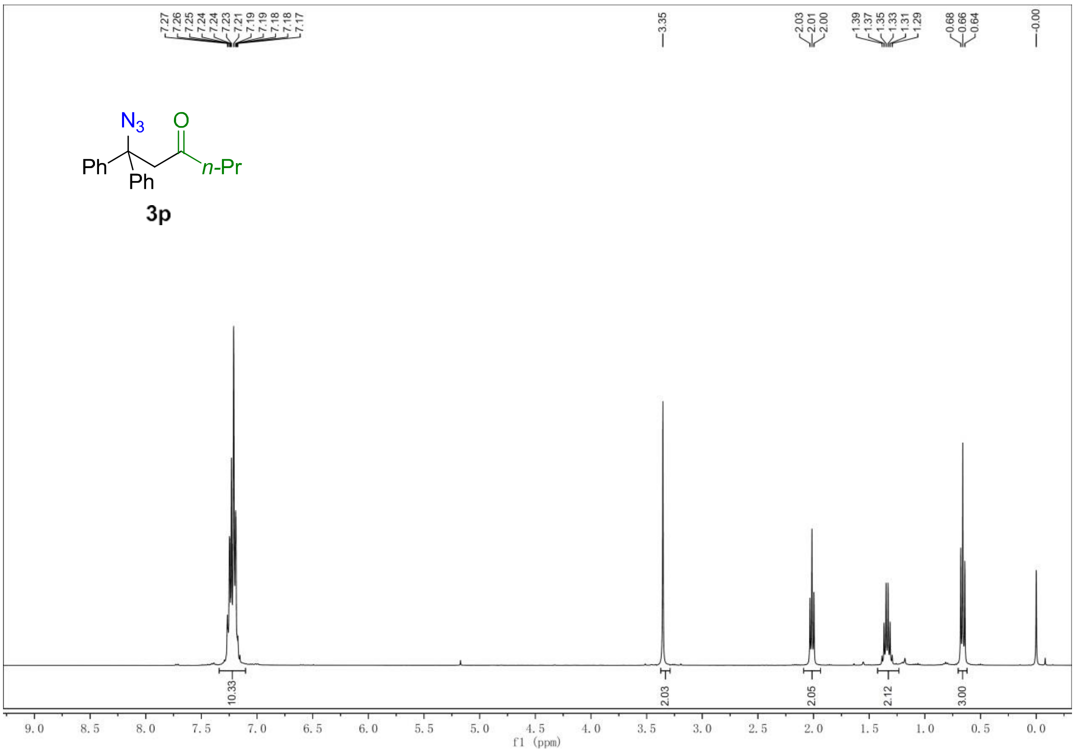

^{13}C NMR spectrum of compound **3p** ($CDCl_3$, 100MHz)

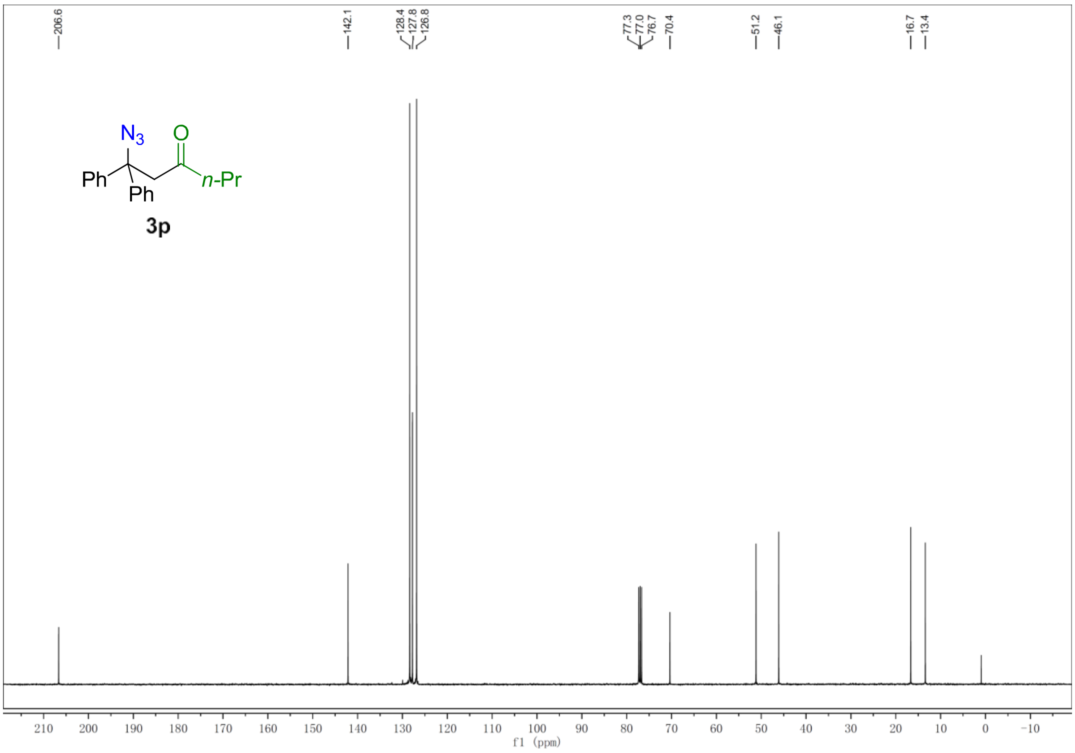

^{1}H NMR spectrum of compound **3q** ($CDCl_3$, 400MHz)

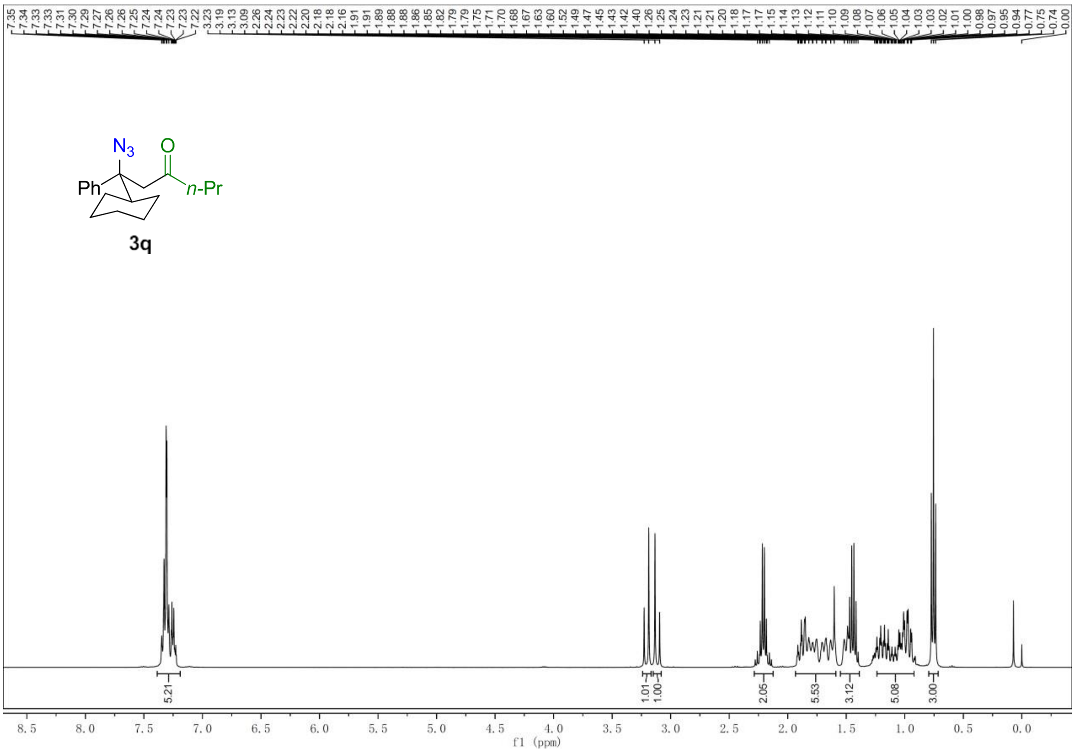

^{13}C NMR spectrum of compound **3q** ($CDCl_3$, 100MHz)

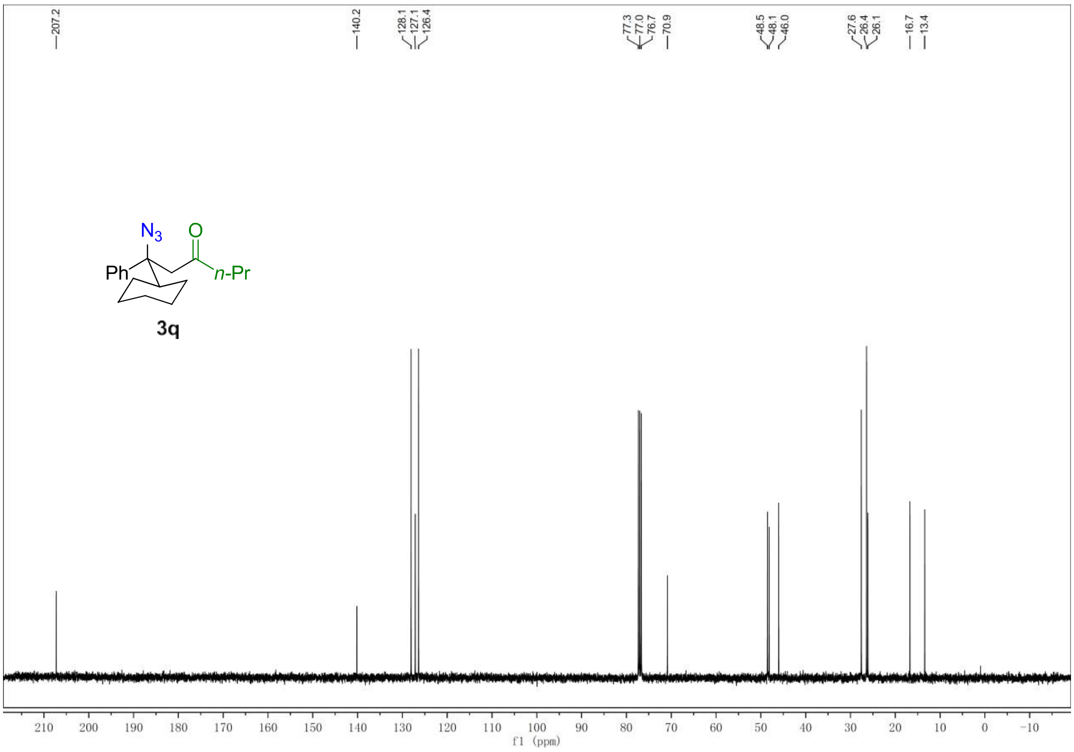

^{1}H NMR spectrum of compound **3r** ($CDCl_3$, 400MHz)

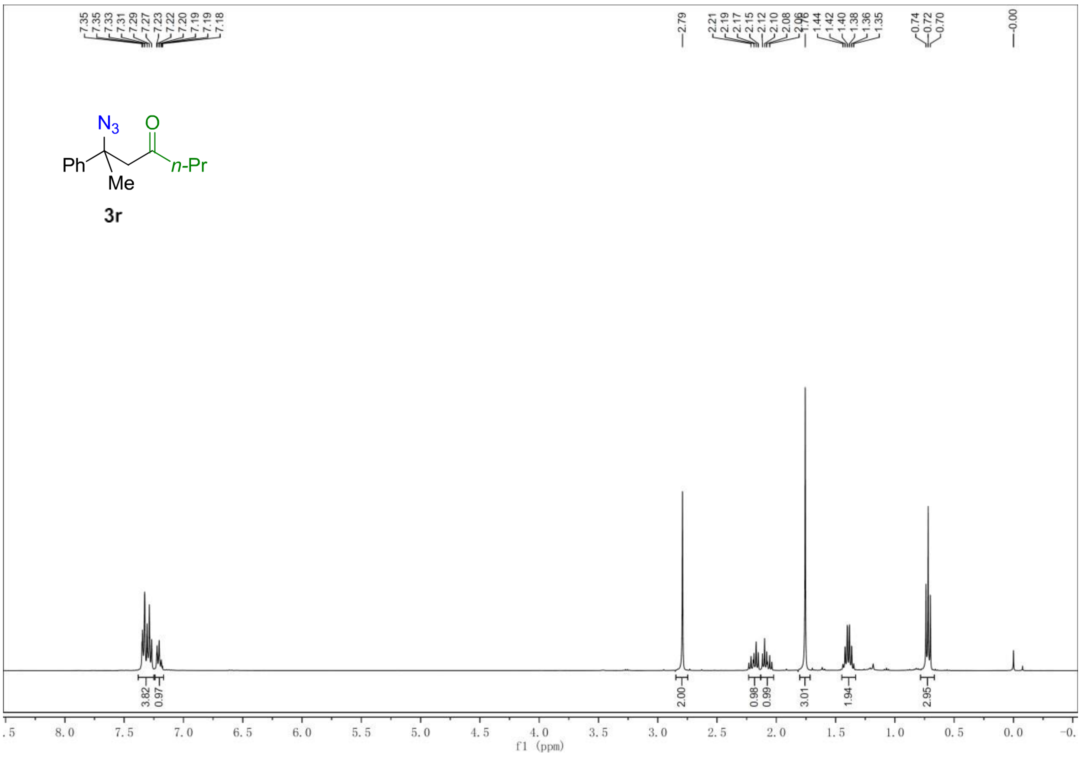

^{13}C NMR spectrum of compound **3r** ($CDCl_3$, 100MHz)

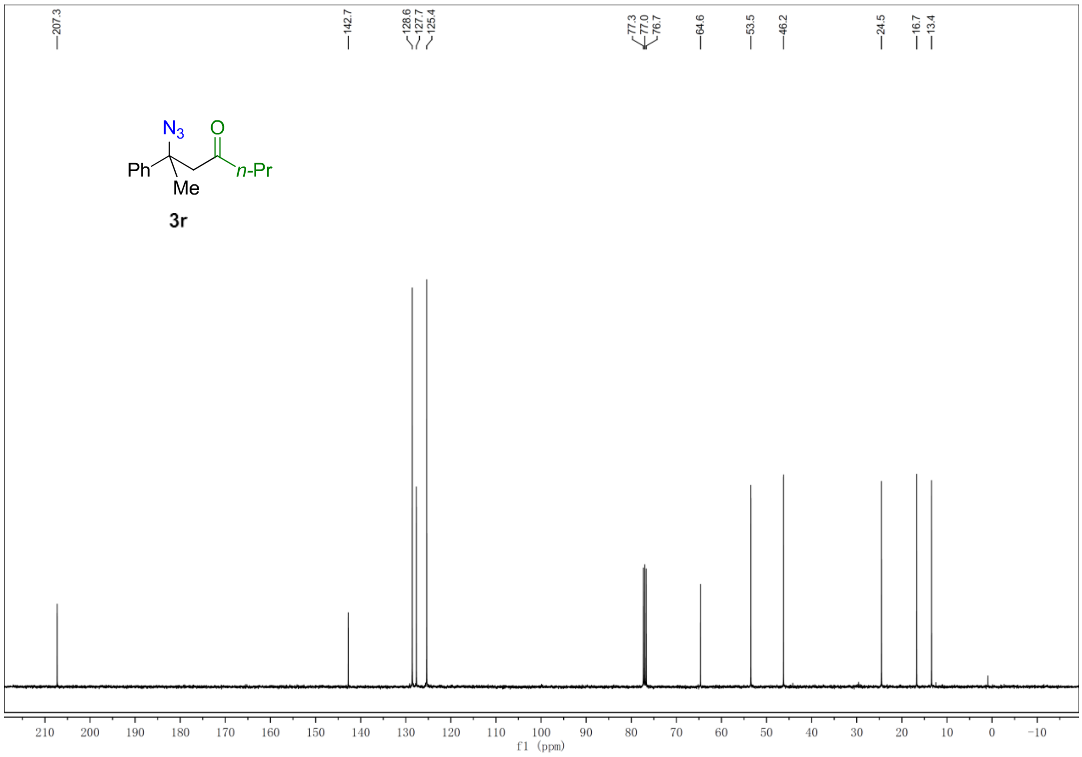

^{1}H NMR spectrum of compound **3s** ($CDCl_3$, 400MHz)

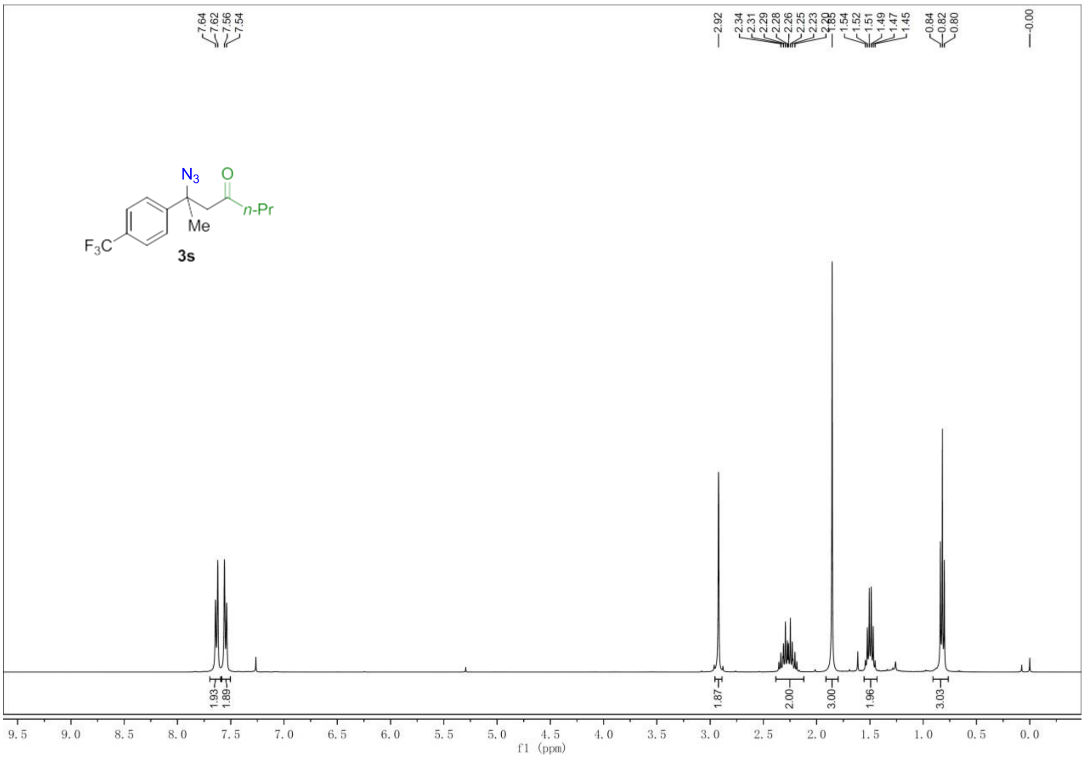

^{13}C NMR spectrum of compound **3s** ($CDCl_3$, 100MHz)

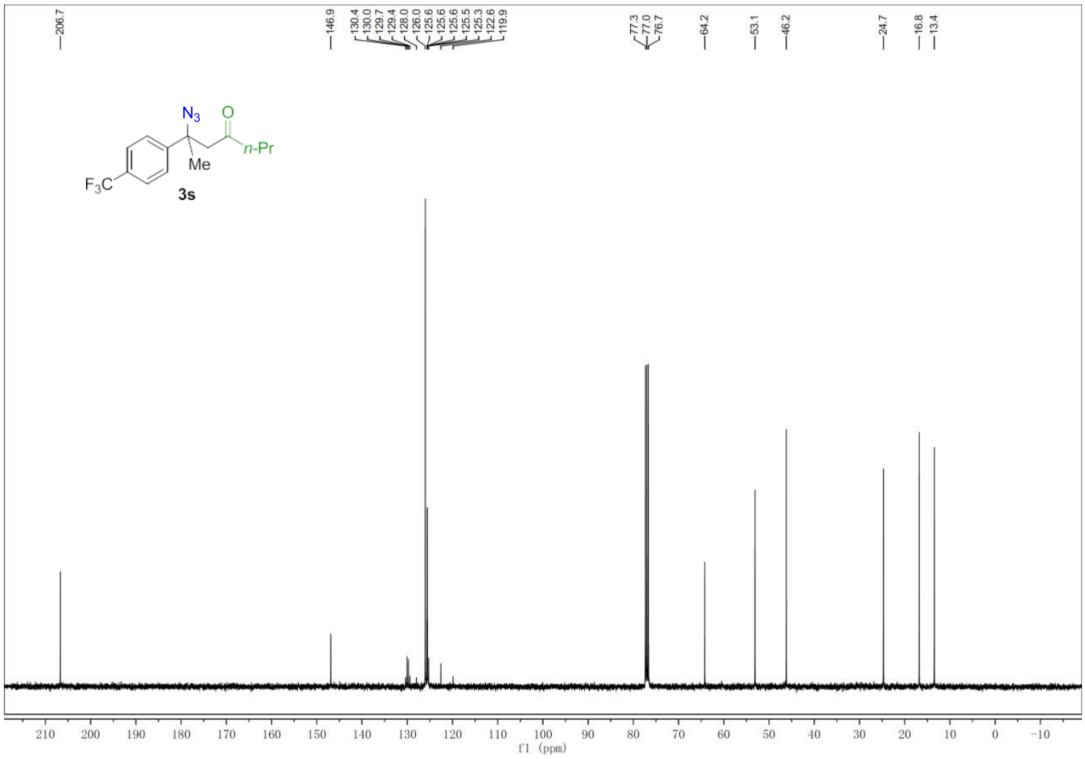

^{1}H NMR spectrum of compound **3t** ($CDCl_3$, 400MHz)

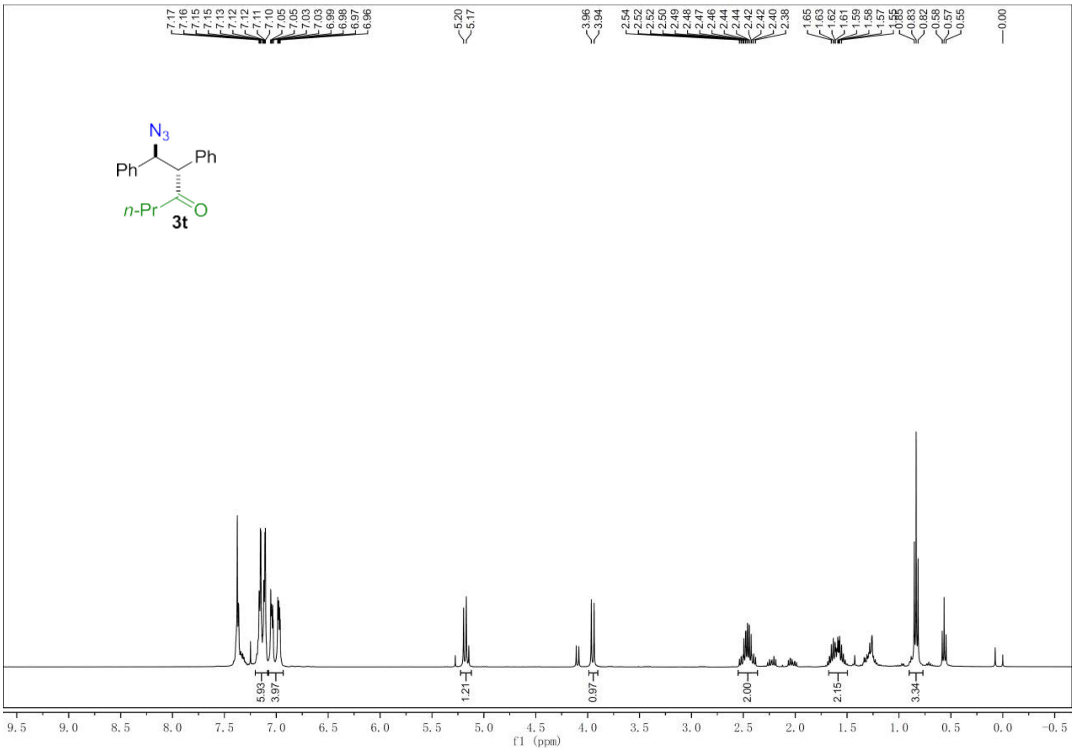

^{13}C NMR spectrum of compound **3t** ($CDCl_3$, 100MHz)

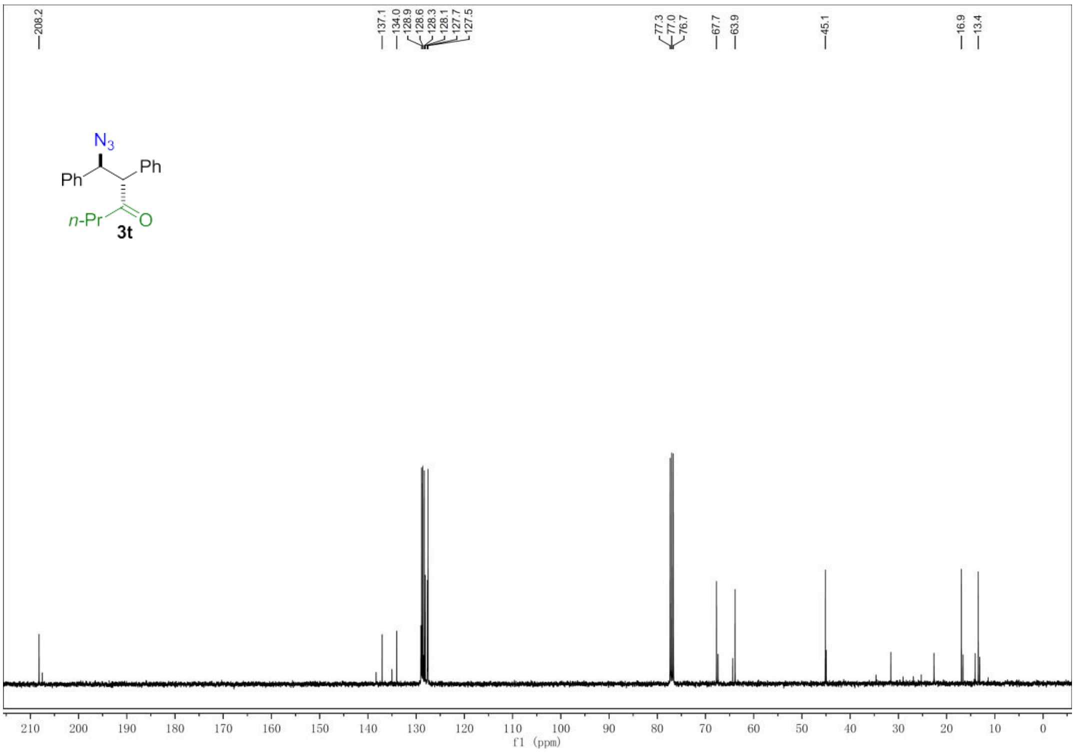

^{1}H NMR spectrum of compound **3u** ($CDCl_3$, 400MHz)

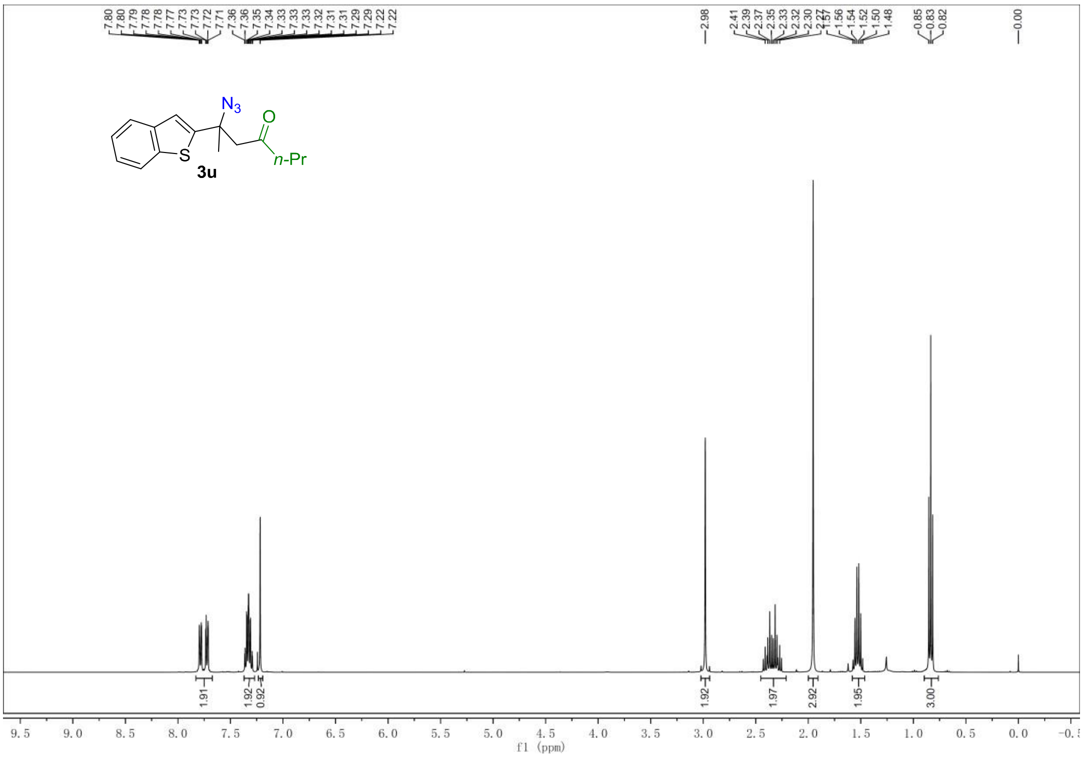

^{13}C NMR spectrum of compound **3u**($CDCl_3$, 100MHz)

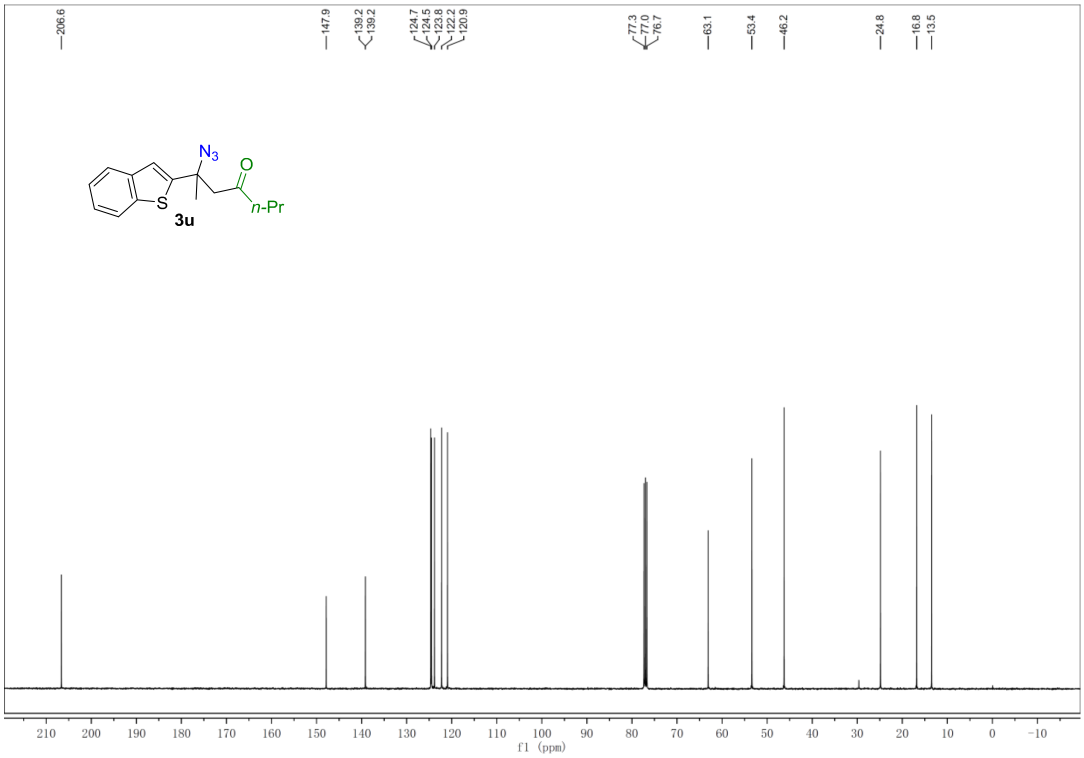

^{1}H NMR spectrum of compound **3v** ($CDCl_3$, 400MHz)

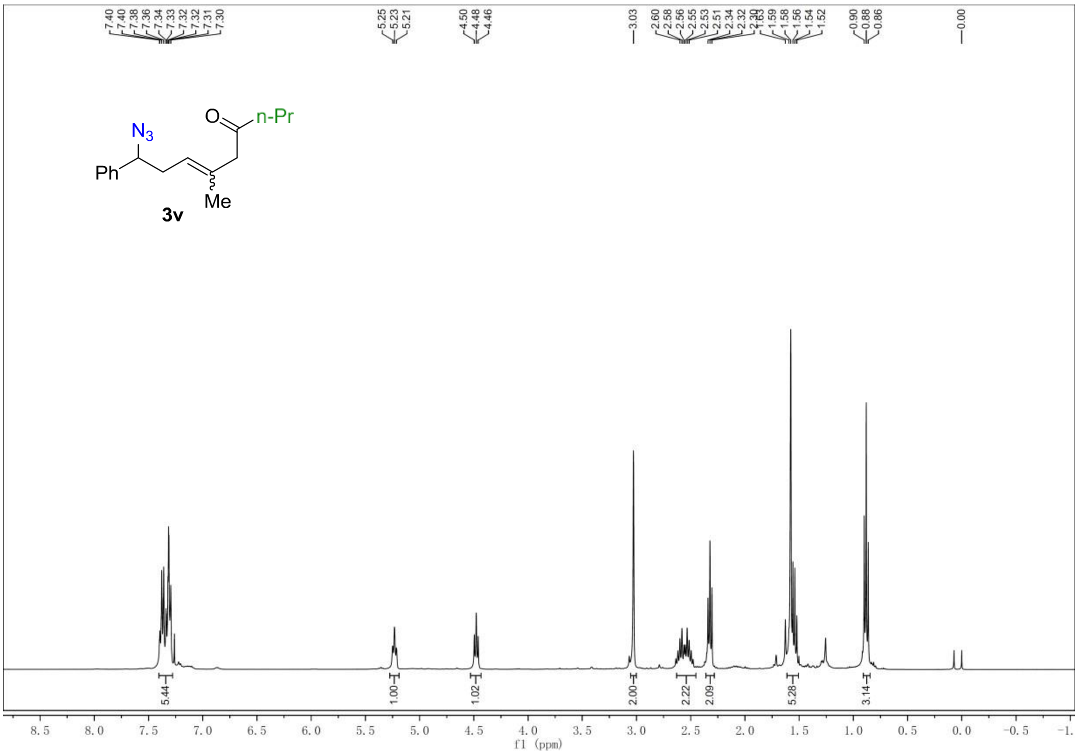

^{13}C NMR spectrum of compound **3v**($CDCl_3$, 100MHz)

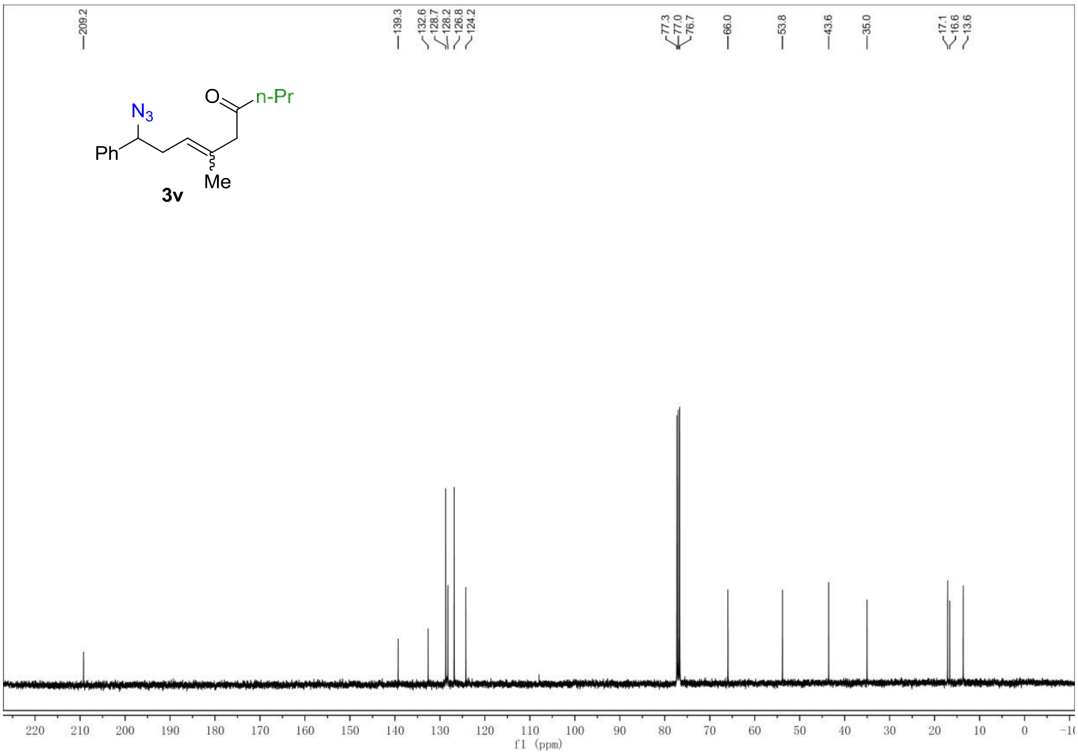

^{1}H NMR spectrum of compound **3w** ($CDCl_3$, 400MHz)

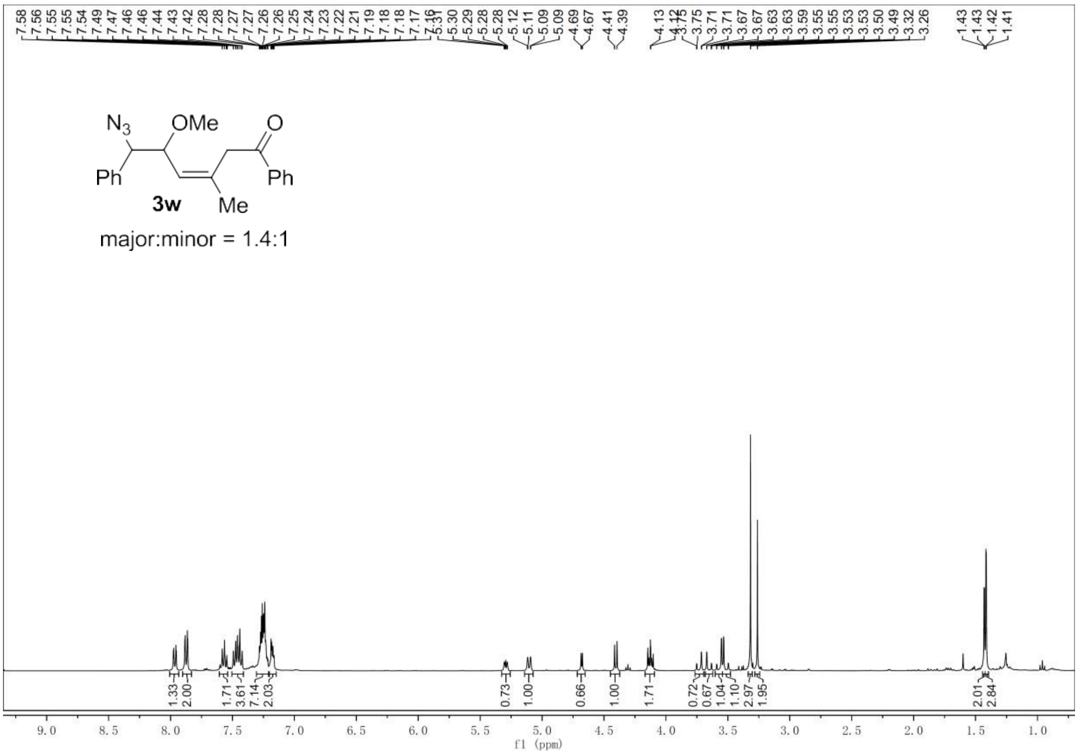

^{13}C NMR spectrum of compound **3w** ($CDCl_3$, 100MHz)

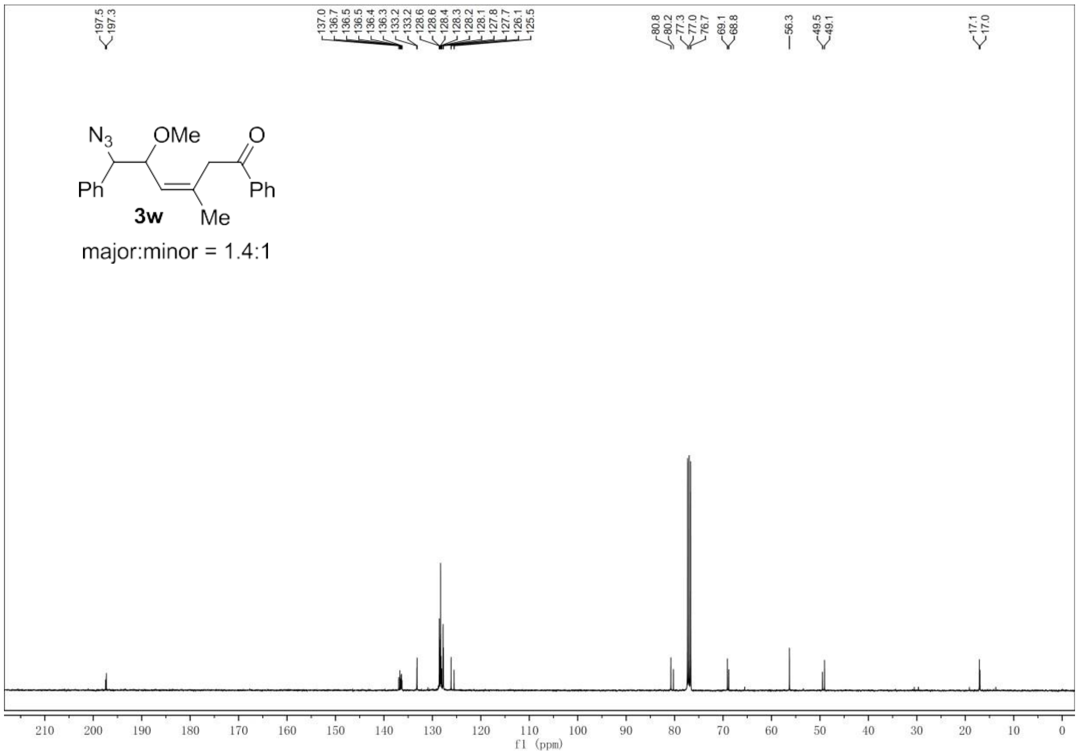

HMBC spectrum of compound **3w**

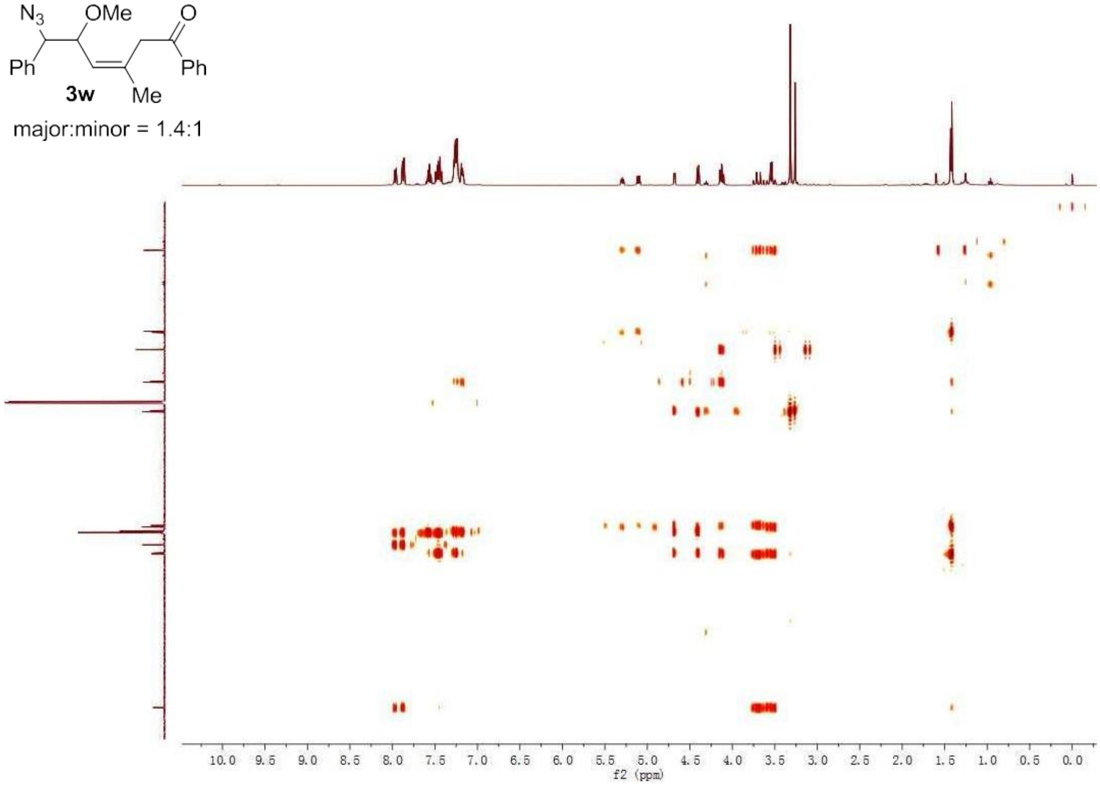

HMQC spectrum of compound **3w**

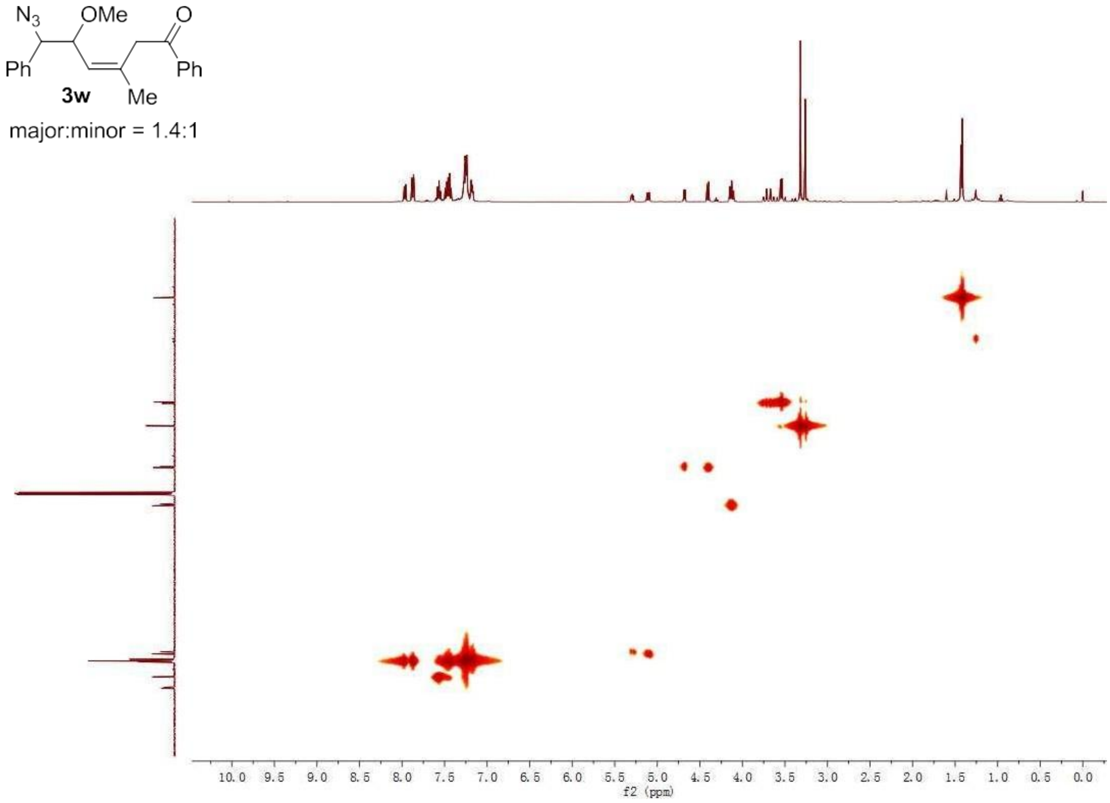

^{1}H NMR spectrum of compound **3aa** ($CDCl_3$, 400MHz)

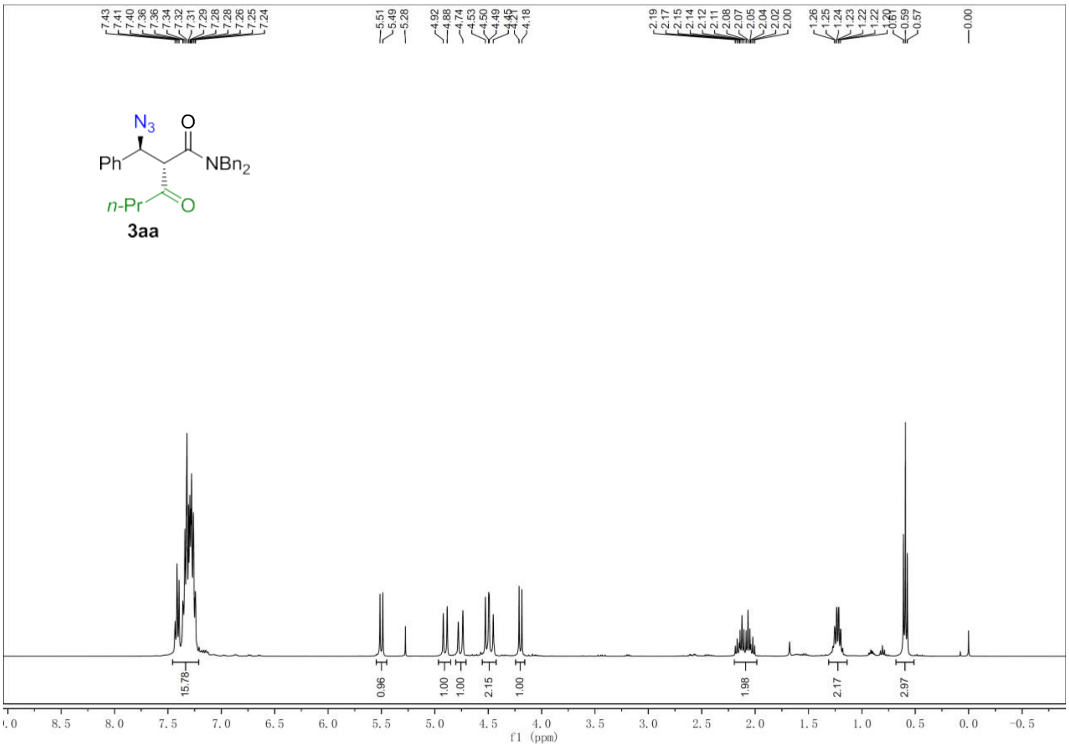

^{13}C NMR spectrum of compound **3aa** ($CDCl_3$, 100MHz)

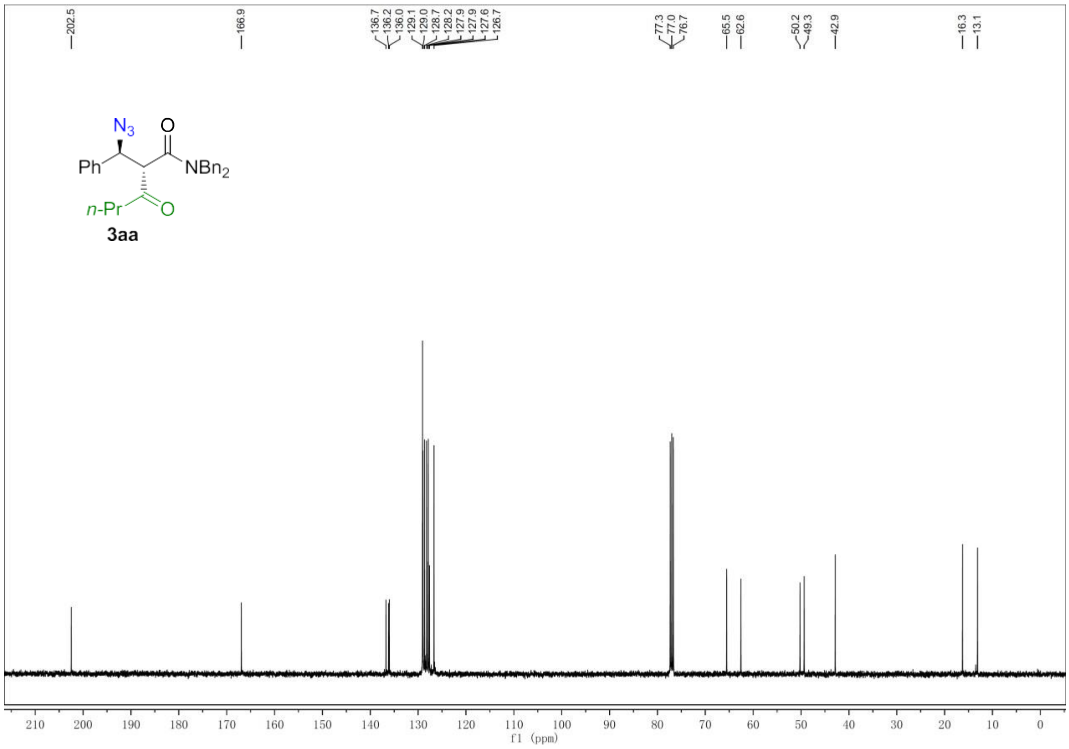

^{1}H NMR spectrum of compound **3ab** ($CDCl_3$, 400MHz)

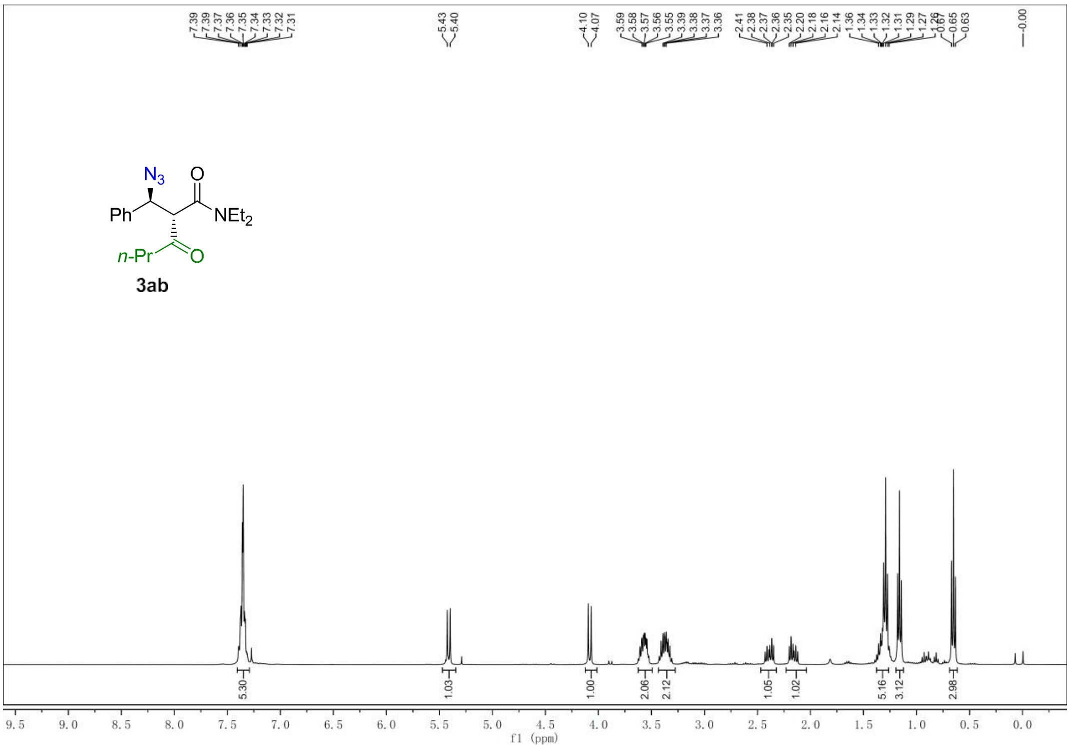

^{13}C NMR spectrum of compound **3ab** ($CDCl_3$, 100MHz)

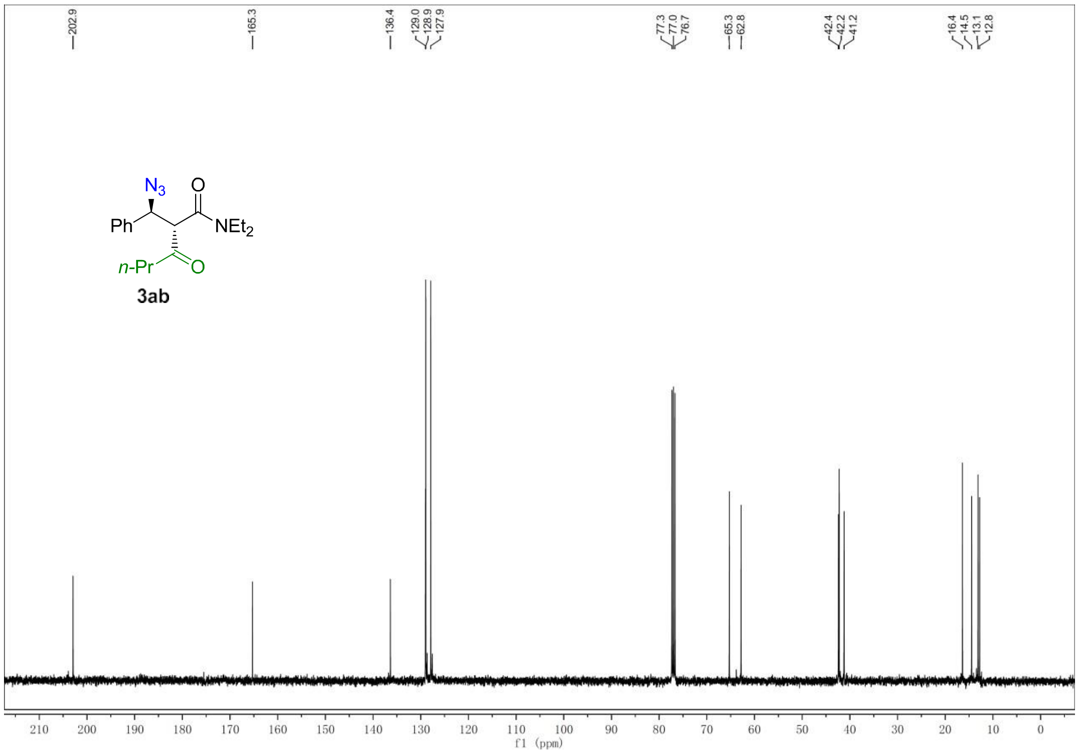

^{1}H NMR spectrum of compound **3ac** ($CDCl_3$, 400MHz)

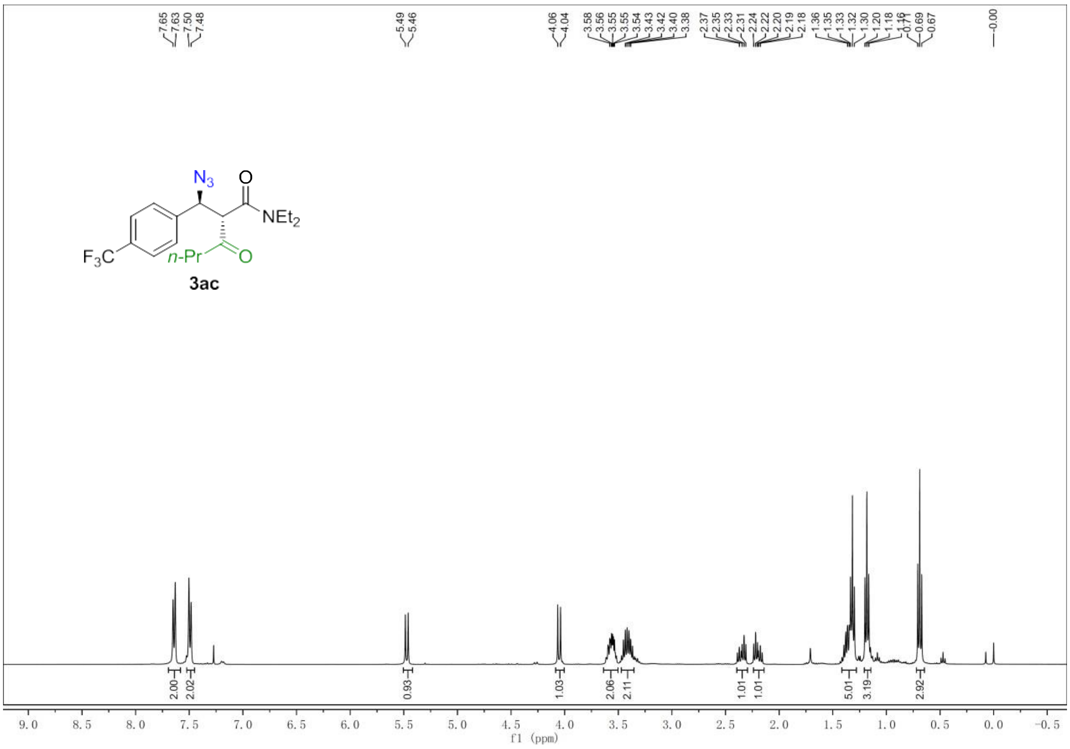

^{13}C NMR spectrum of compound **3ac** ($CDCl_3$, 100MHz)

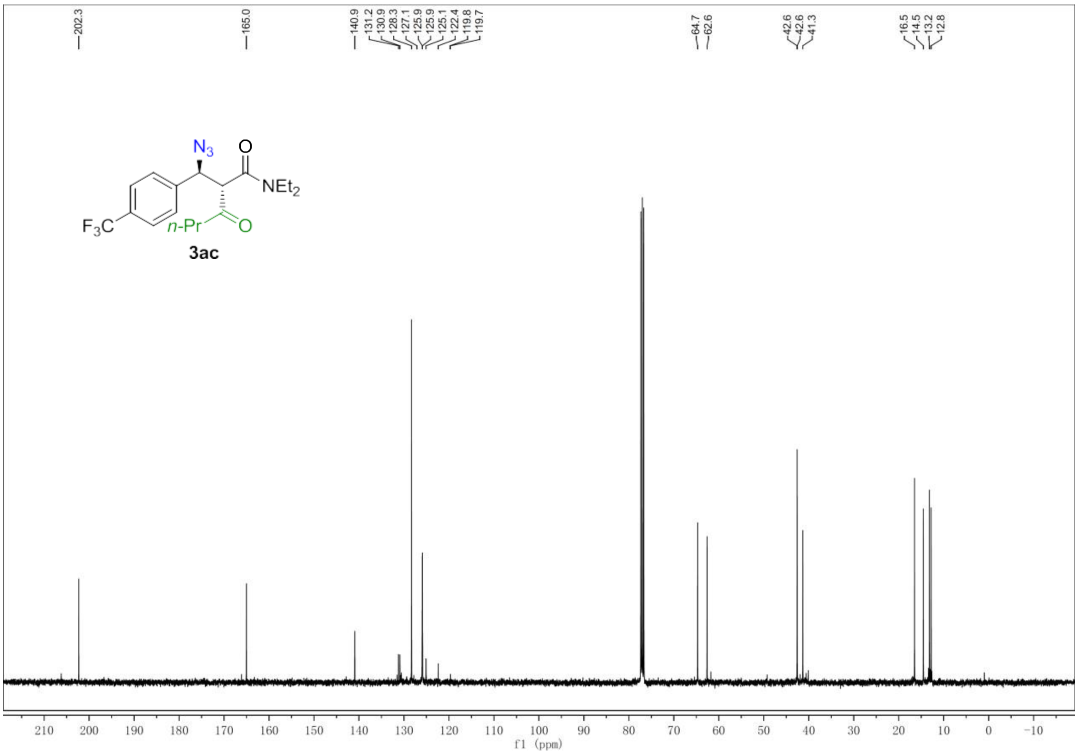

^{1}H NMR spectrum of compound **3ad** ($CDCl_3$, 400MHz)

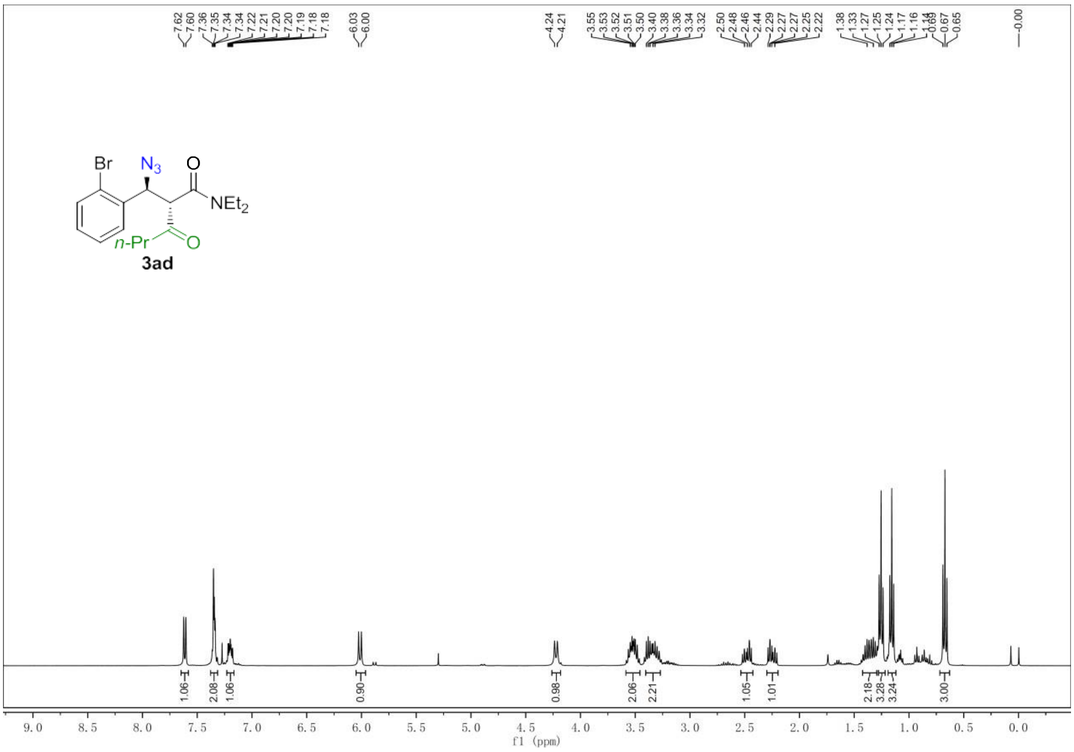

^{13}C NMR spectrum of compound **3ad** ($CDCl_3$, 100MHz)

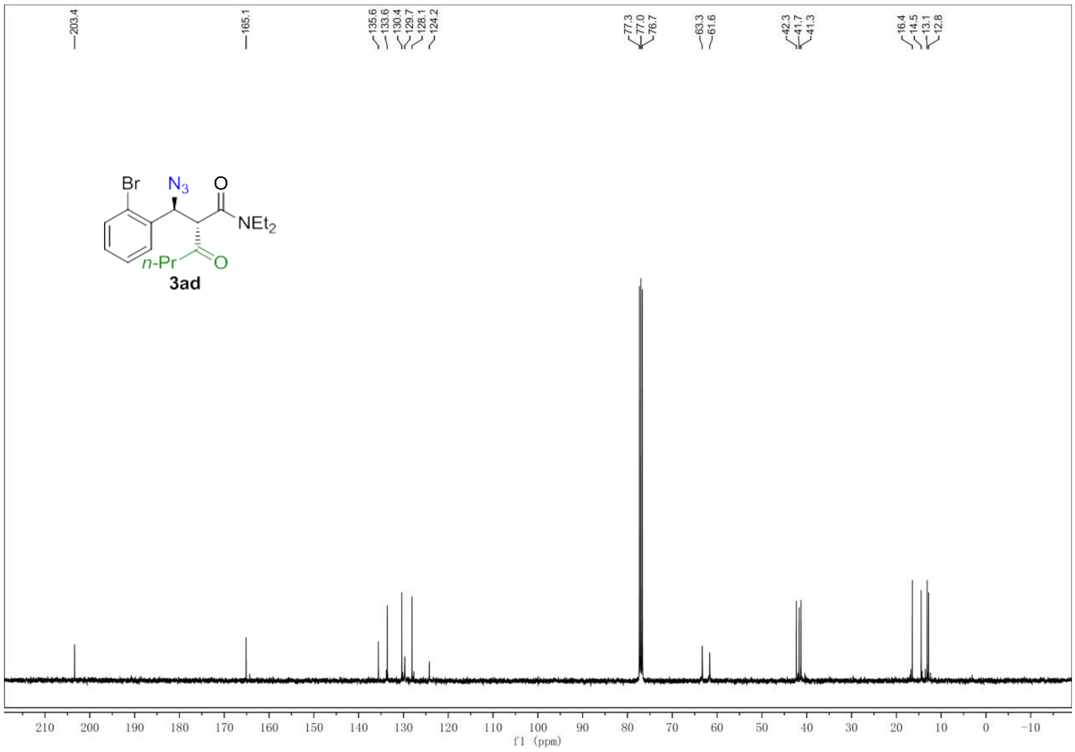

^{1}H NMR spectrum of compound **3ae** ($CDCl_3$, 400MHz)

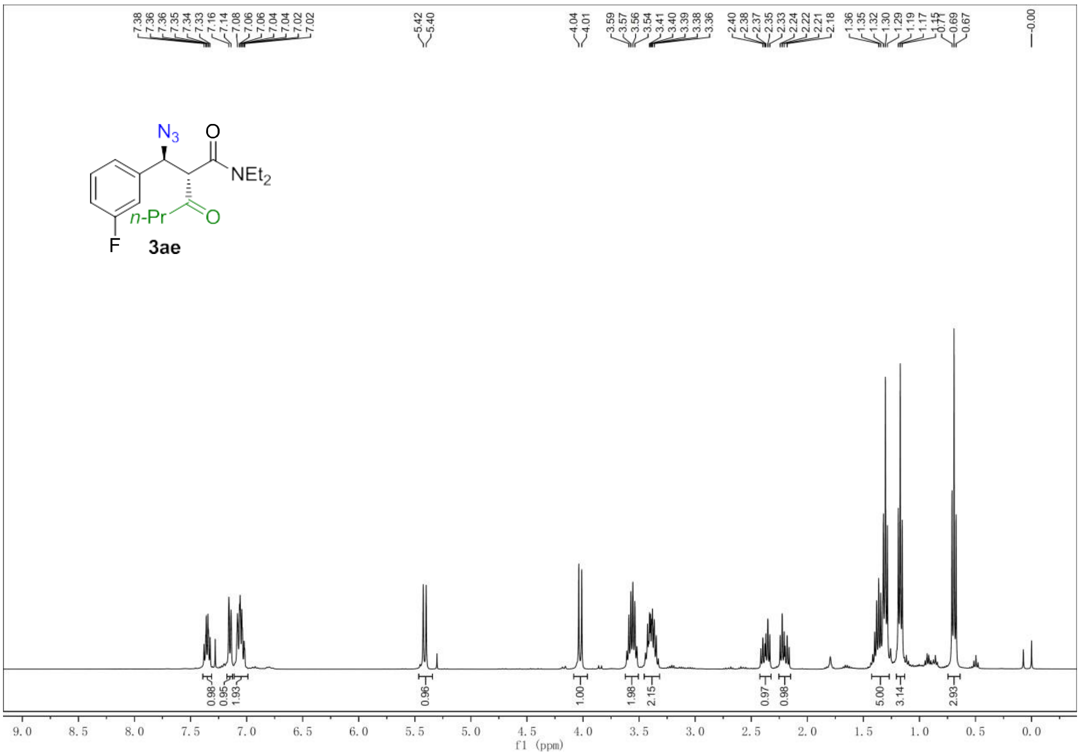

^{13}C NMR spectrum of compound **3ae** ($CDCl_3$, 100MHz)

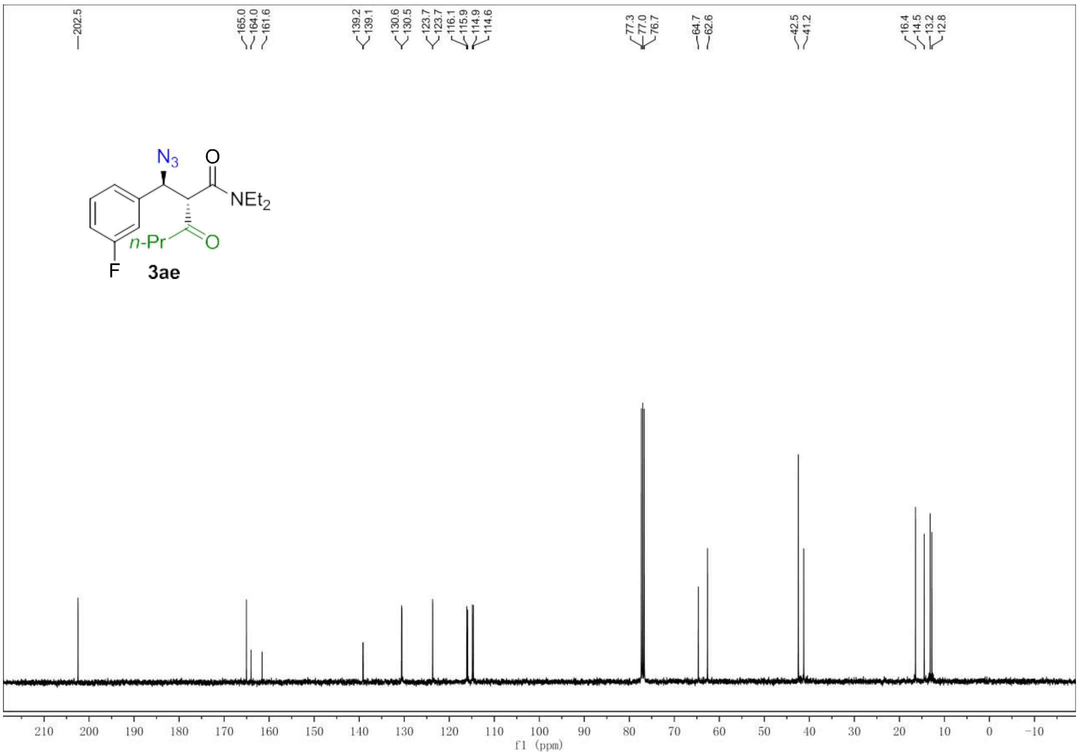

^{1}H NMR spectrum of compound **3af** ($CDCl_3$, 400MHz)

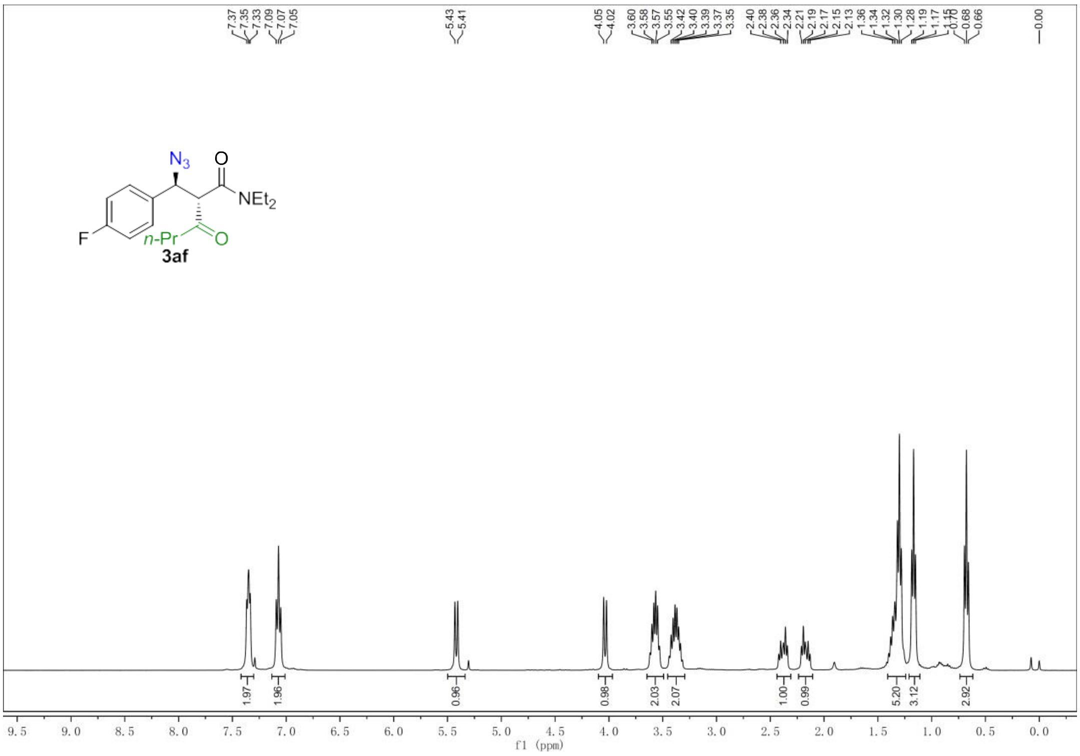

^{13}C NMR spectrum of compound **3af** ($CDCl_3$, 100MHz)

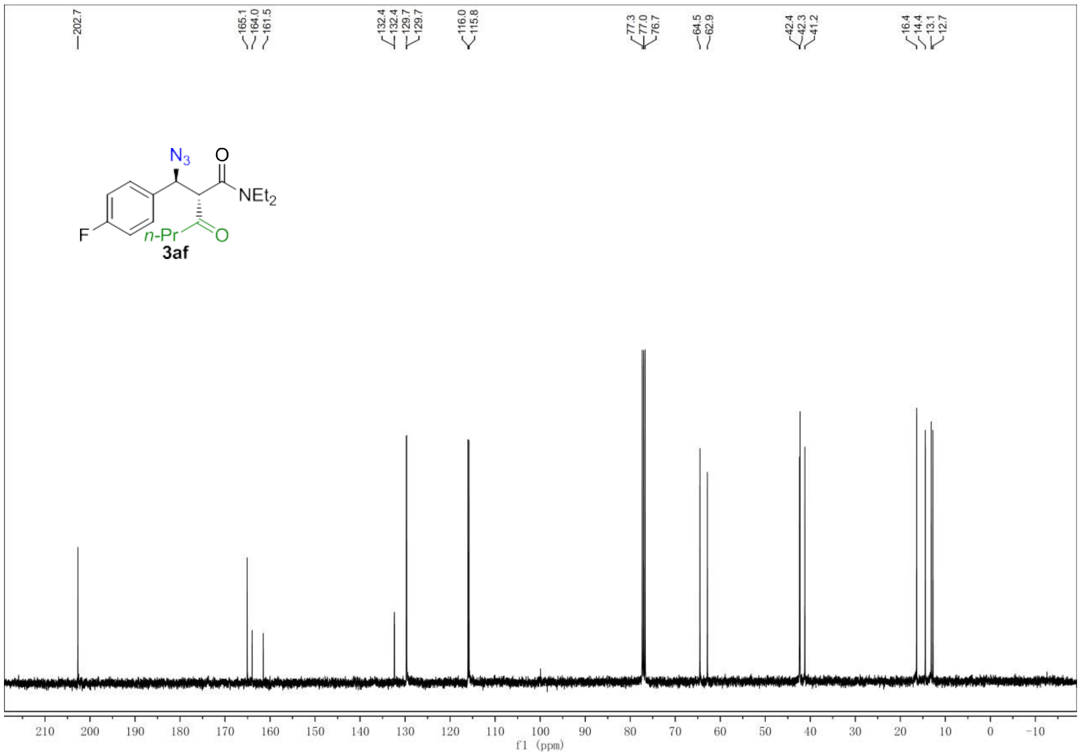

^{1}H NMR spectrum of compound **3ag** ($CDCl_3$, 400MHz)

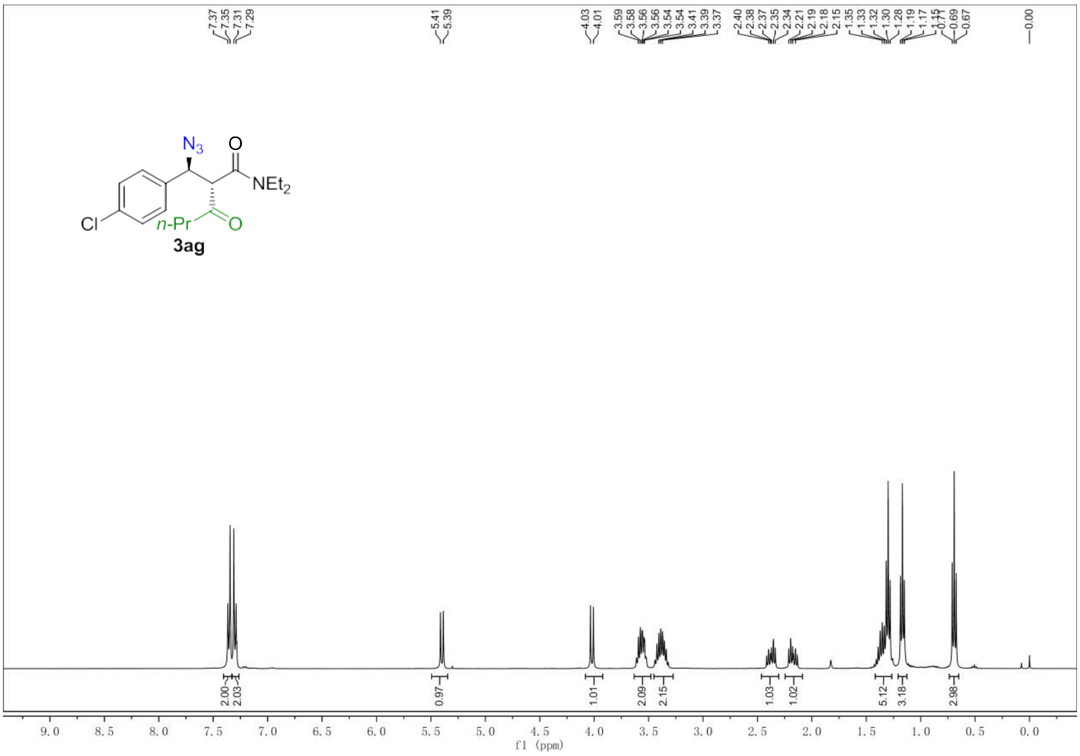

^{13}C NMR spectrum of compound **3ag** ($CDCl_3$, 100MHz)

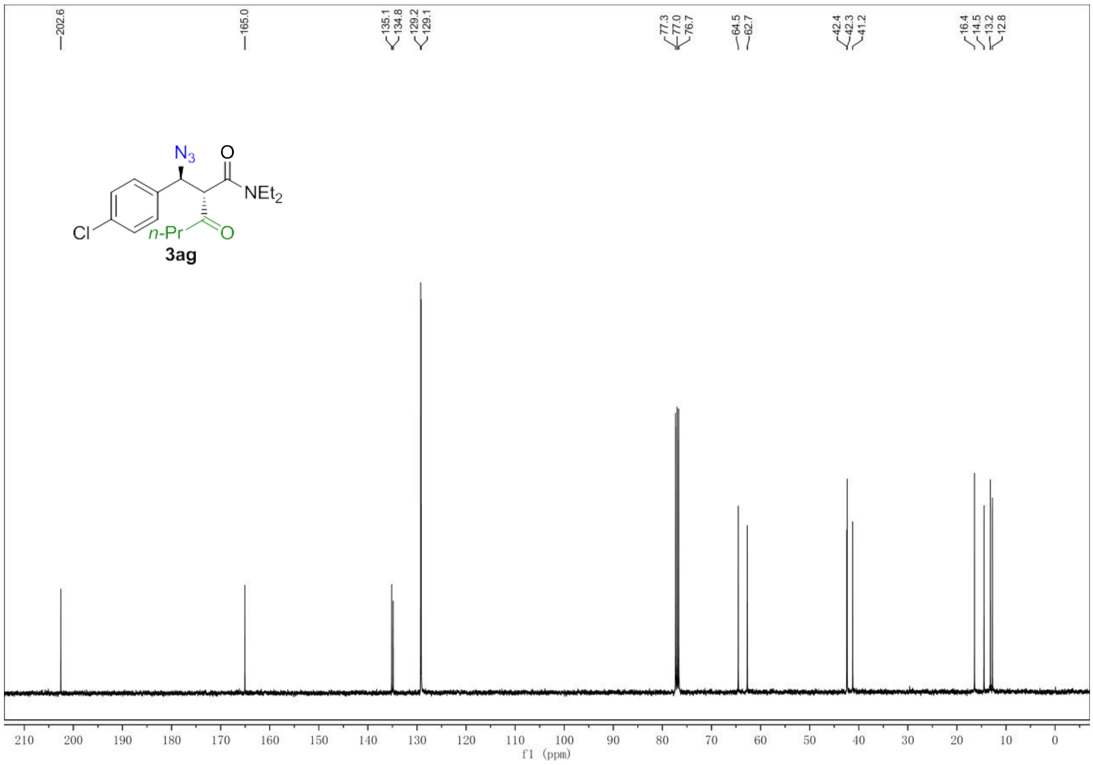

^{1}H NMR spectrum of compound **3ah** ($CDCl_3$, 400MHz)

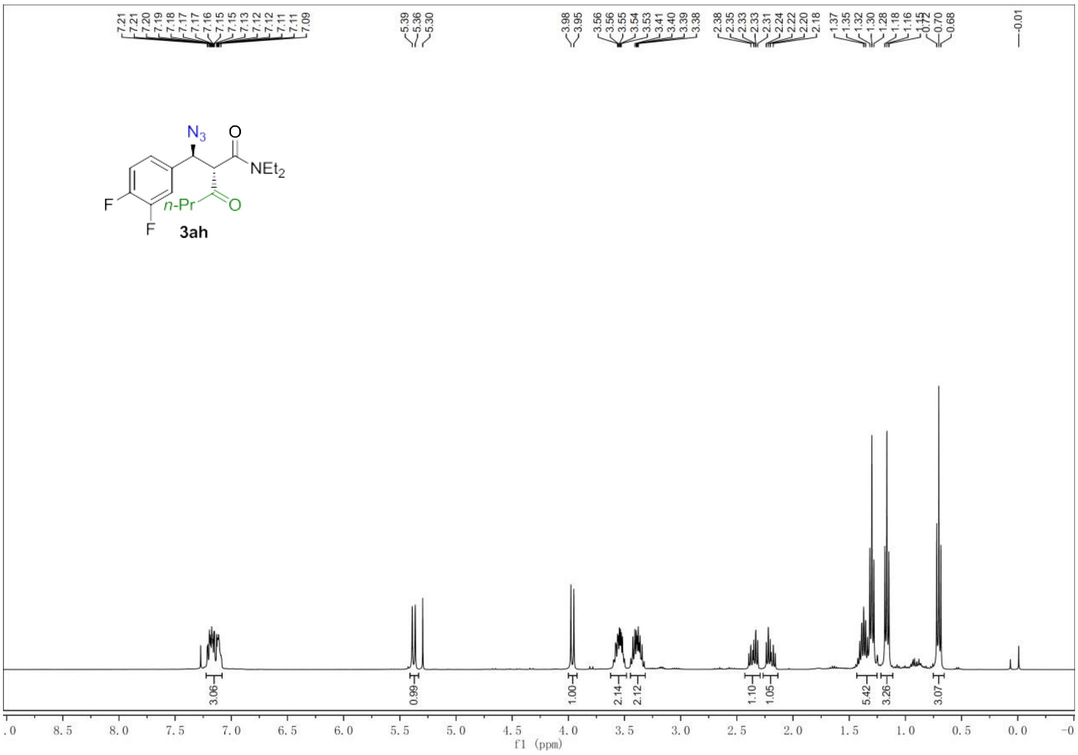

^{13}C NMR spectrum of compound **3ah** ($CDCl_3$, 100MHz)

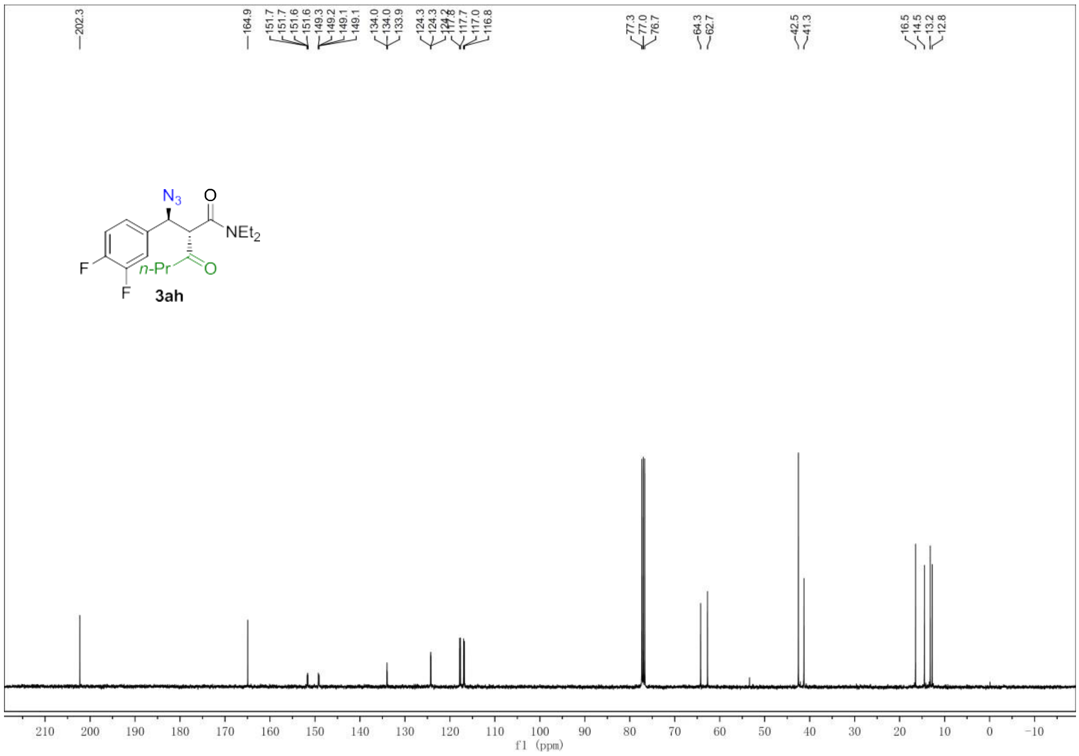

^{1}H NMR spectrum of compound **3ai** ($CDCl_3$, 400MHz)

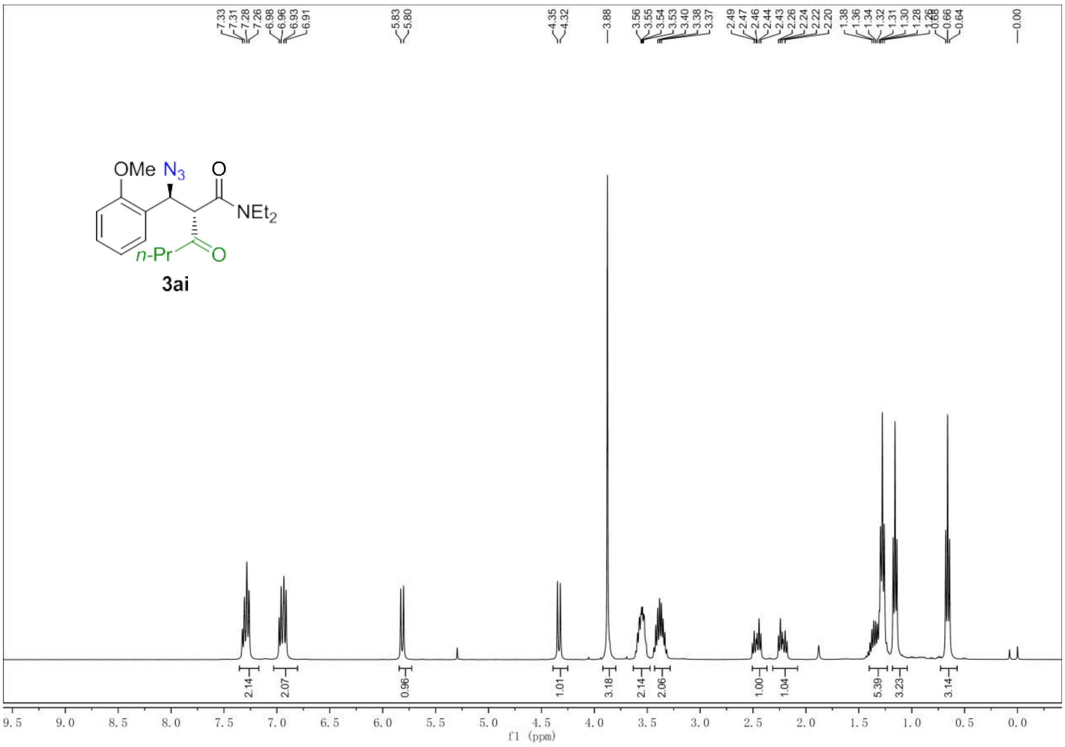

^{13}C NMR spectrum of compound **3ai** ($CDCl_3$, 100MHz)

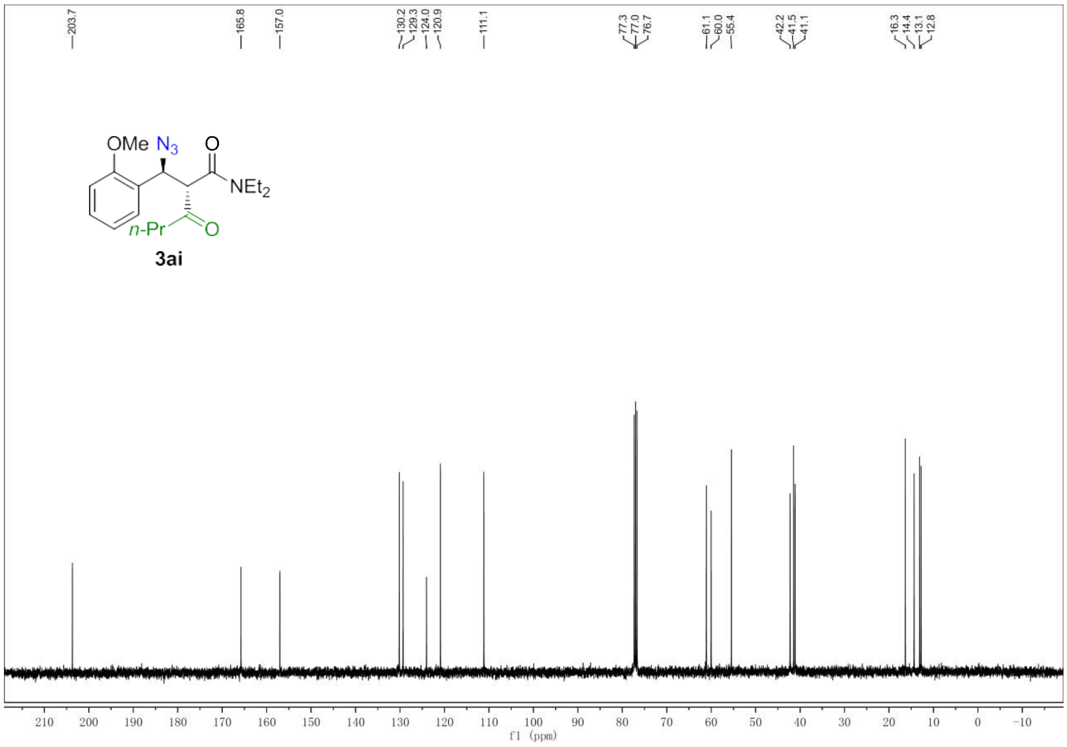

^{1}H NMR spectrum of compound **3aj** ($CDCl_3$, 400MHz)

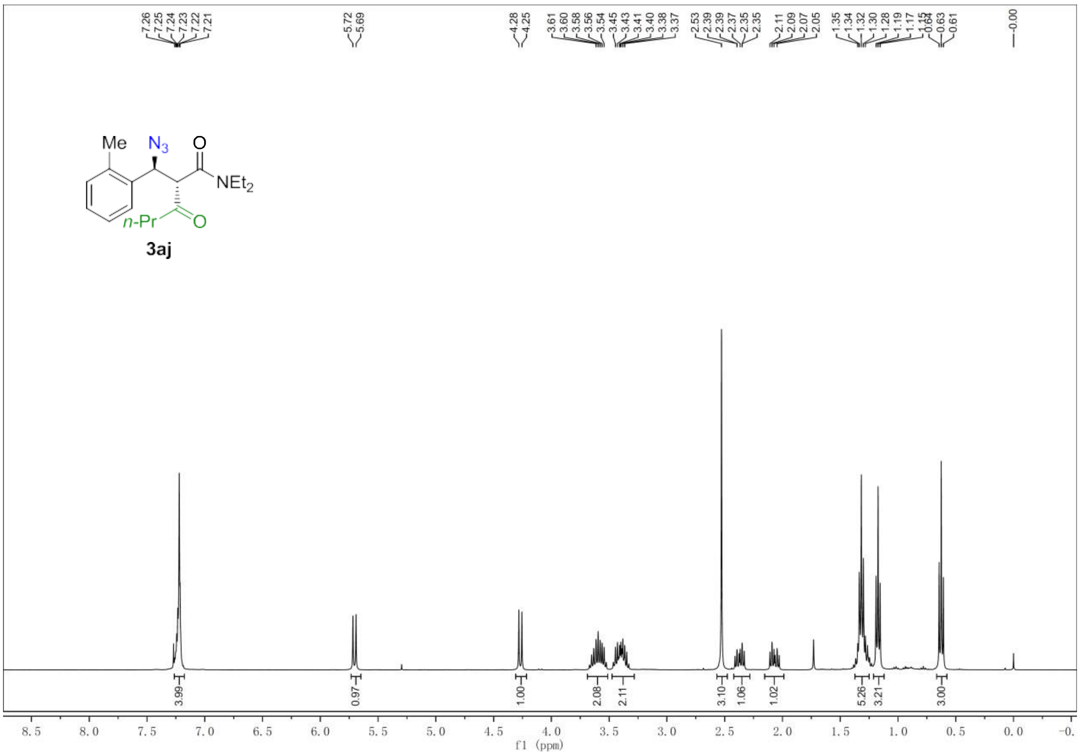

^{13}C NMR spectrum of compound **3aj** ($CDCl_3$, 100MHz)

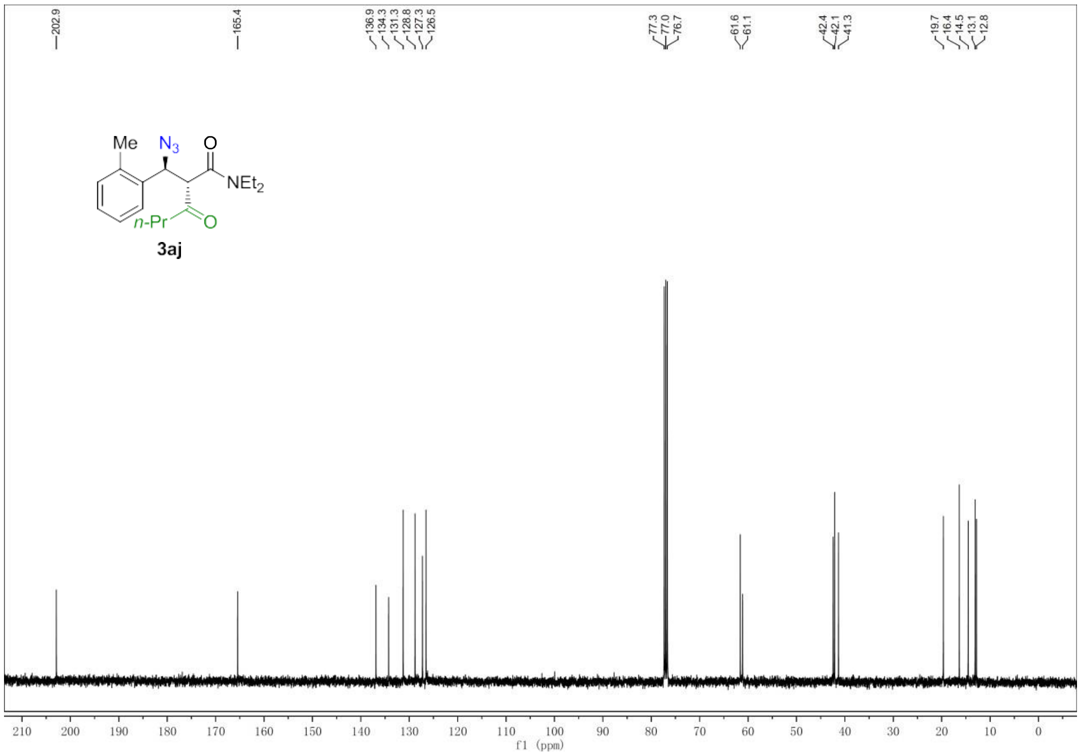

^{1}H NMR spectrum of compound **3ak** ($CDCl_3$, 400MHz)

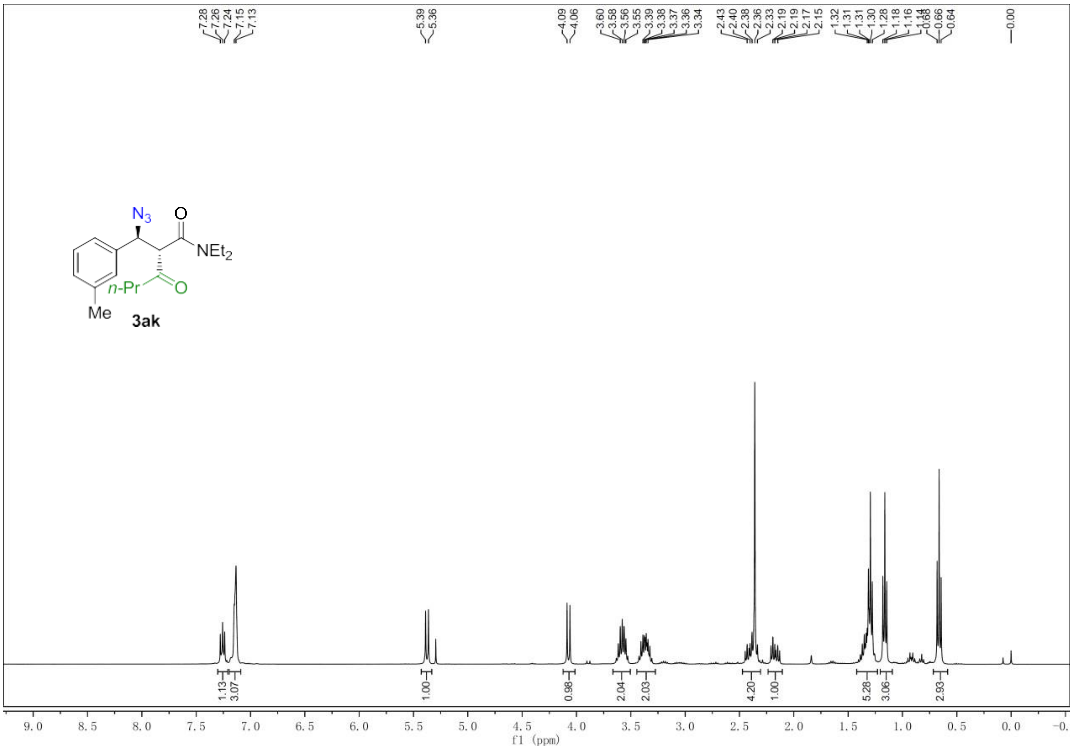

^{13}C NMR spectrum of compound **3ak** ($CDCl_3$, 100MHz)

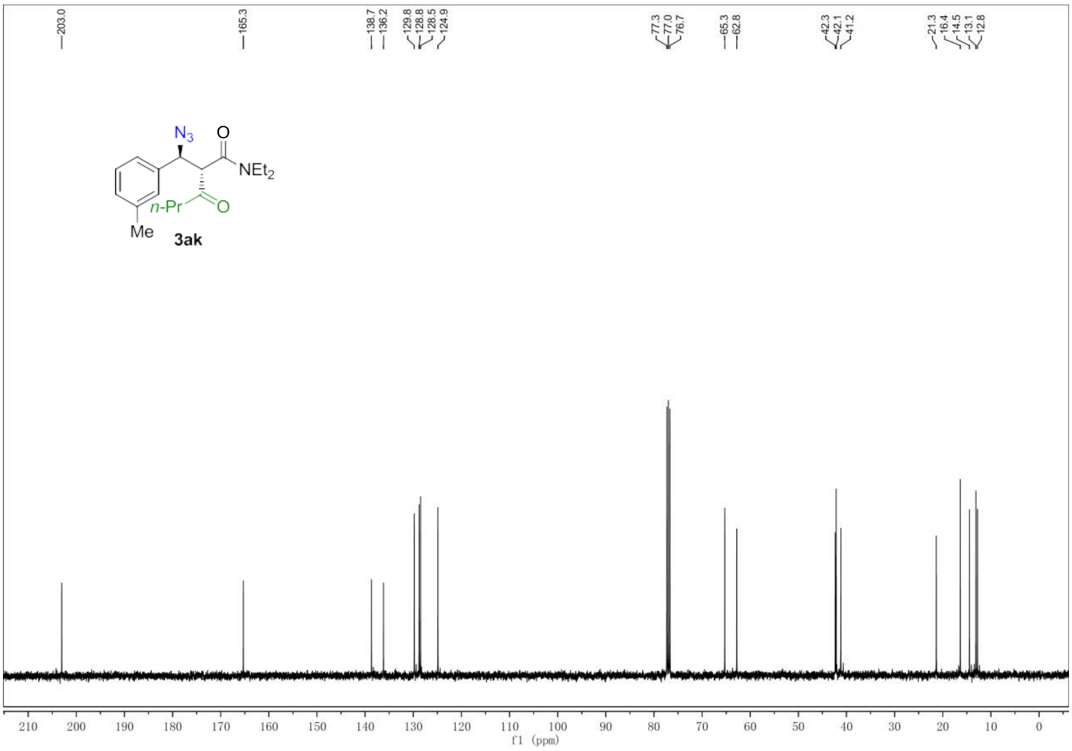

^{1}H NMR spectrum of compound **3al** ($CDCl_3$, 400MHz)

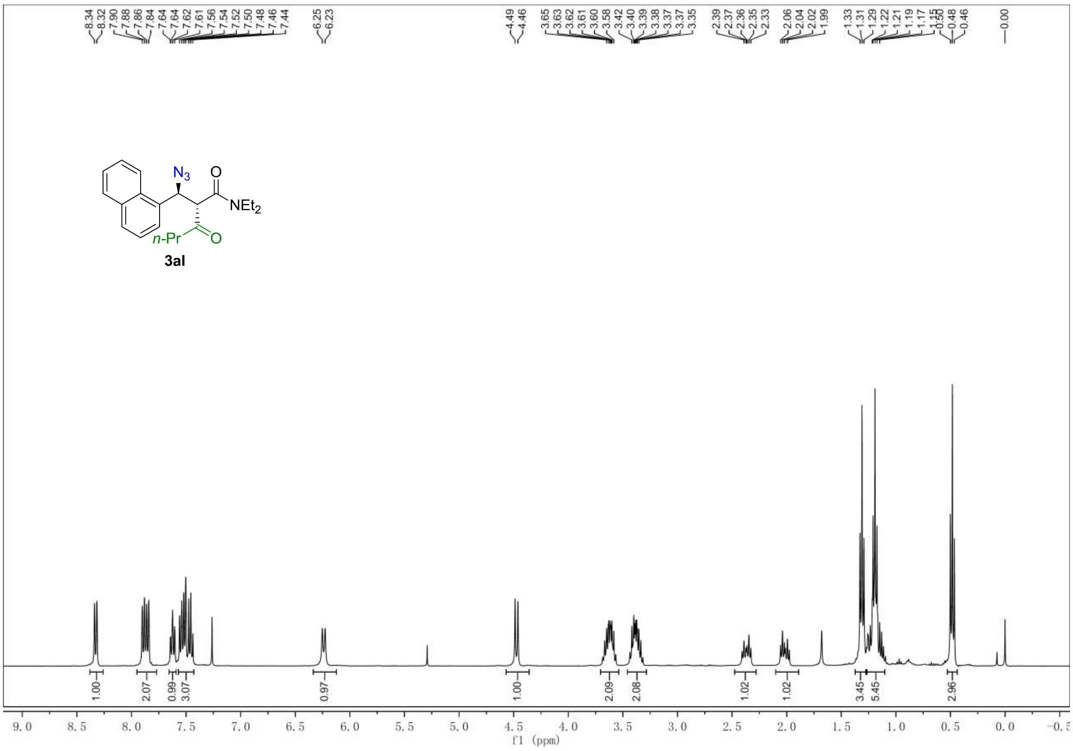

^{13}C NMR spectrum of compound **3al** ($CDCl_3$, 100MHz)

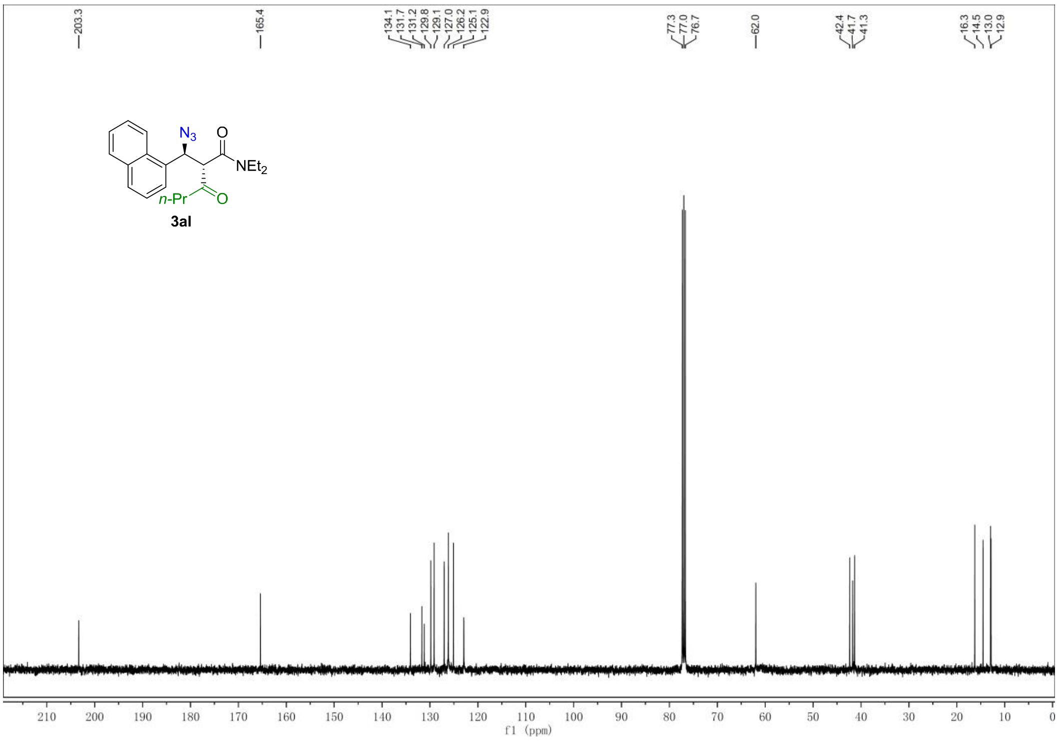

^{1}H NMR spectrum of compound **3am** ($CDCl_3$, 400MHz)

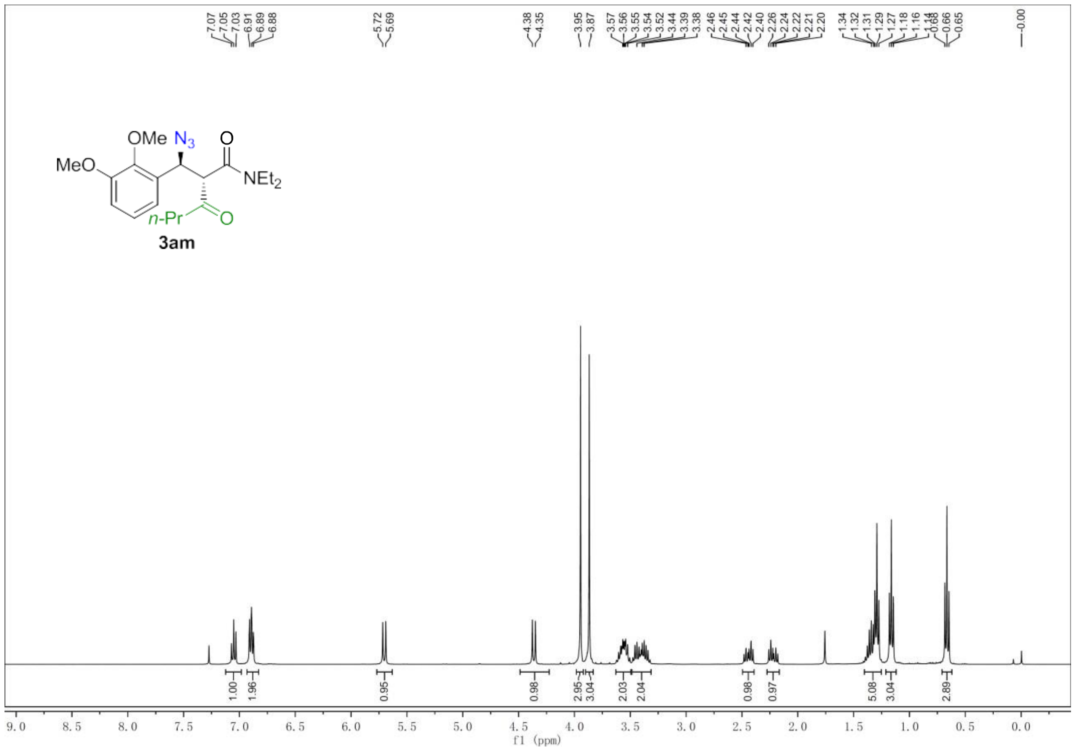

^{13}C NMR spectrum of compound **3am** ($CDCl_3$, 100MHz)

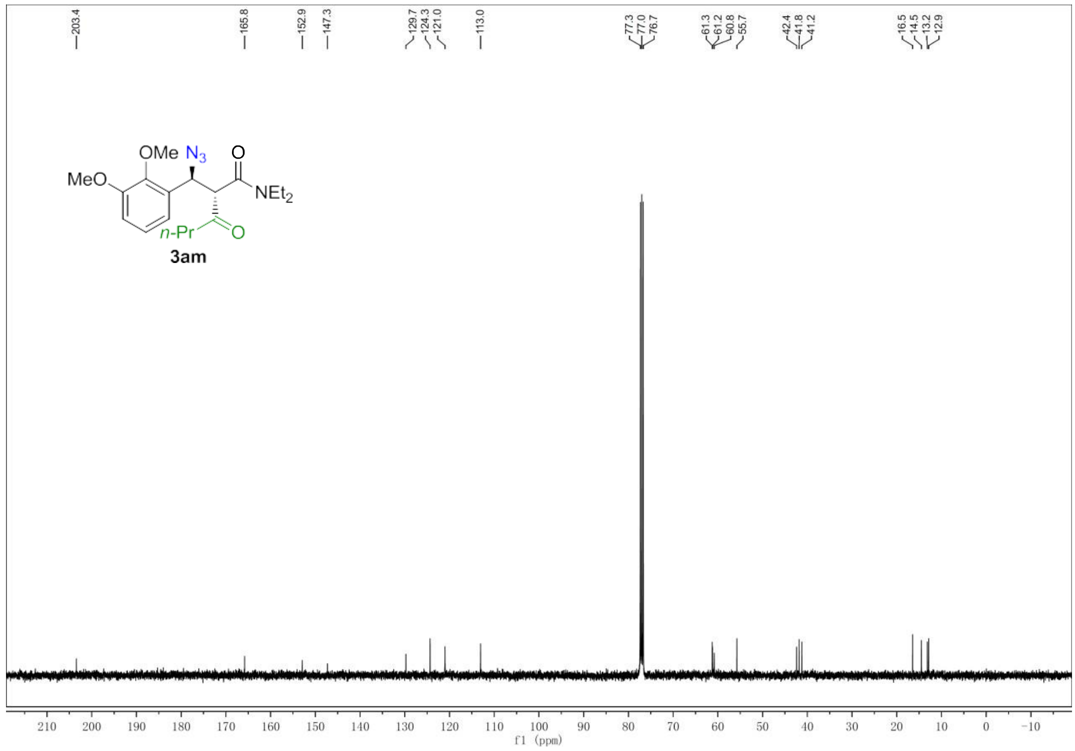

^{1}H NMR spectrum of compound **3an** ($CDCl_3$, 400MHz)

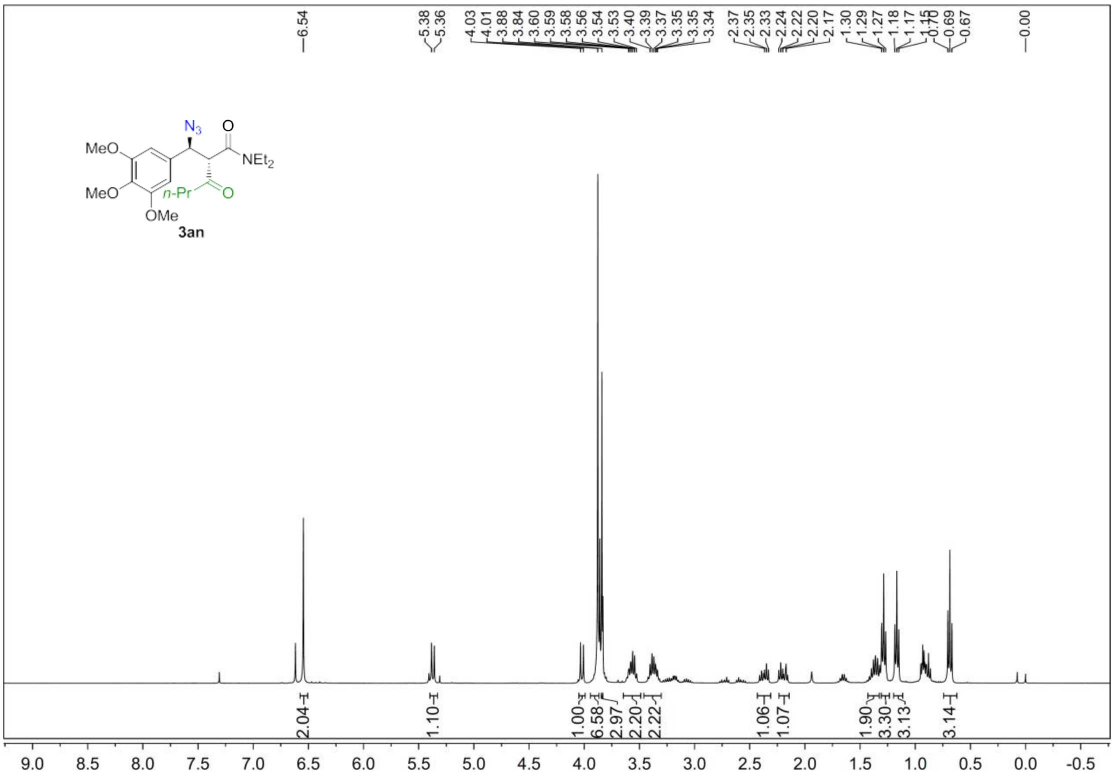

^{13}C NMR spectrum of compound **3an** ($CDCl_3$, 100MHz)

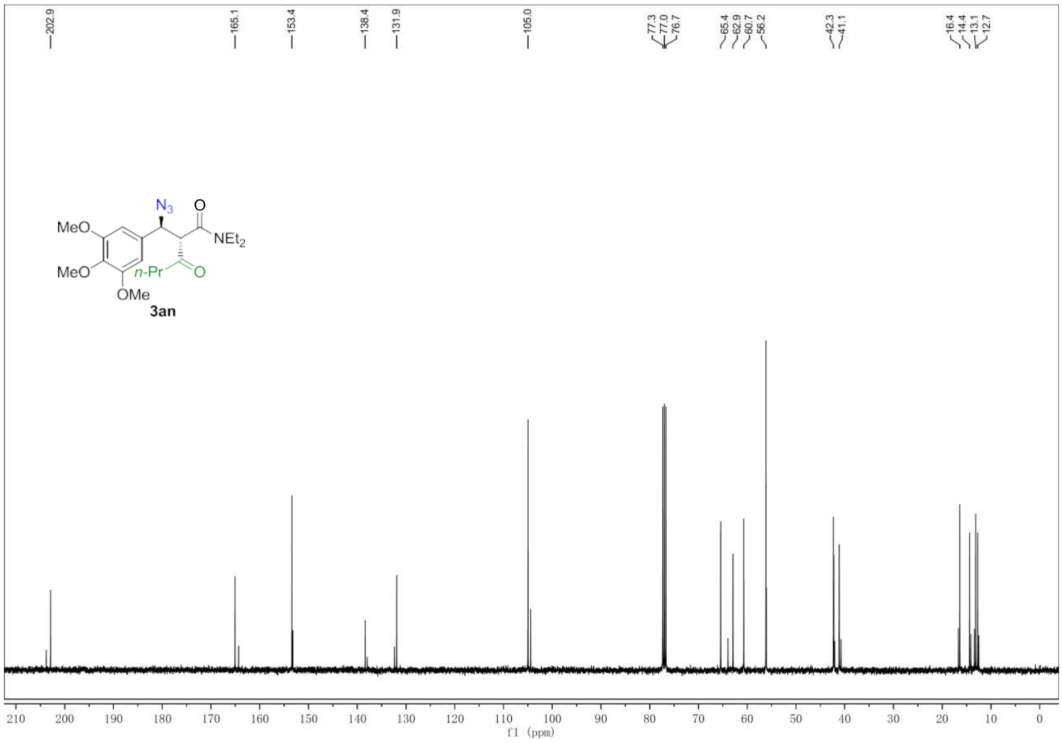

^{1}H NMR spectrum of compound **4a** ($CDCl_3$, 400MHz)

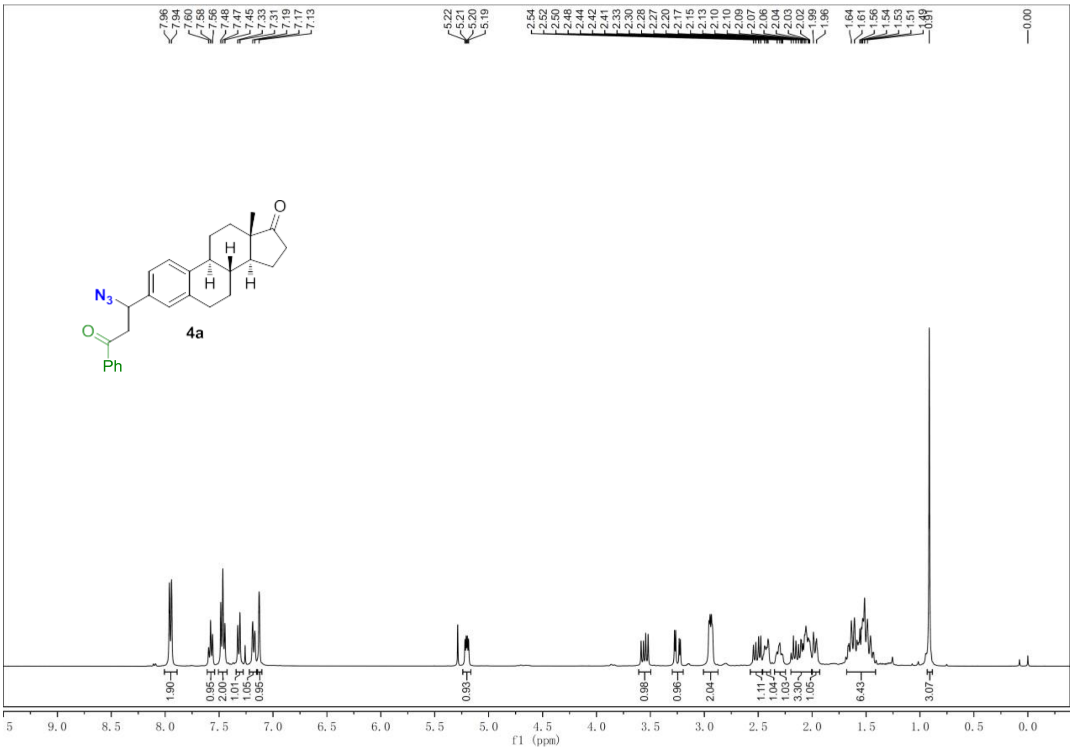

^{13}C NMR spectrum of compound **4a** ($CDCl_3$, 100MHz)

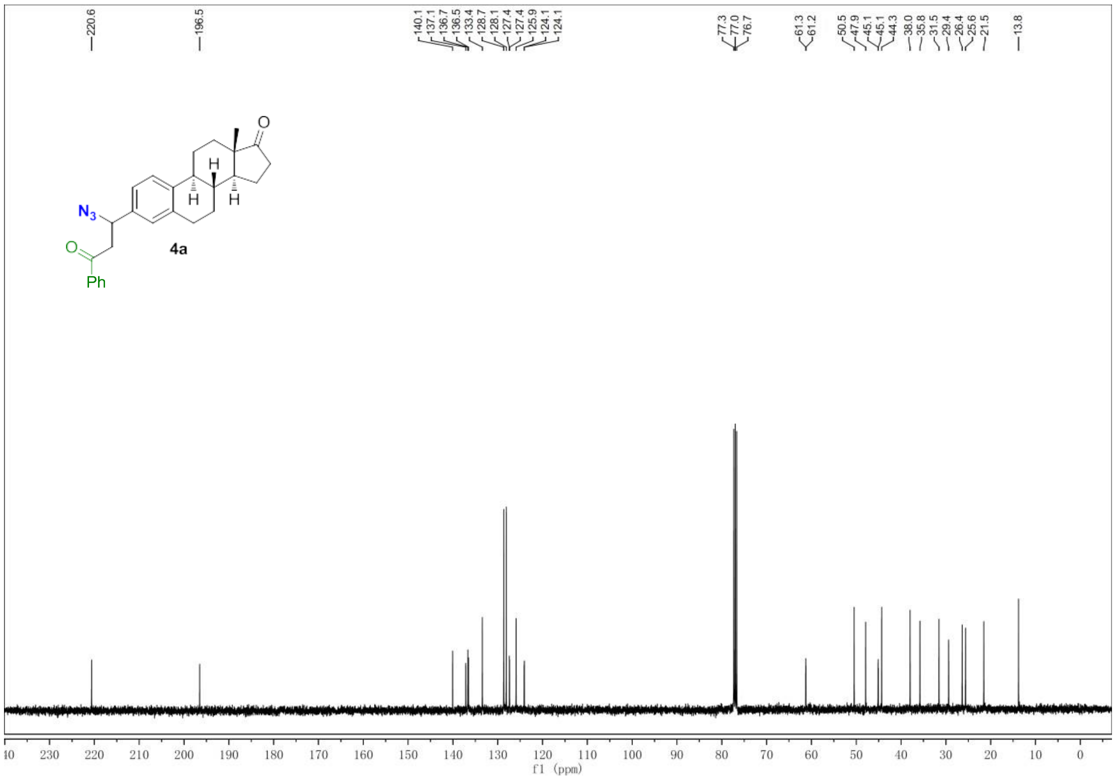

^{1}H NMR spectrum of compound **4b** ($CDCl_3$, 400MHz)

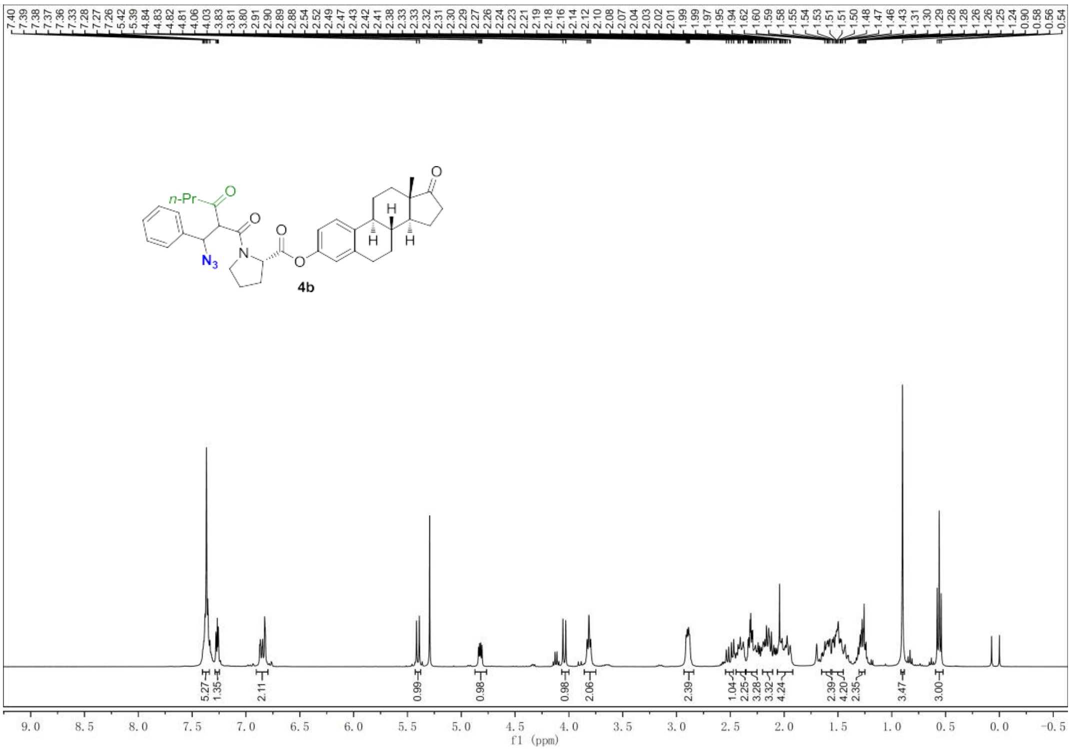

^{13}C NMR spectrum of compound **4b** ($CDCl_3$, 100MHz)

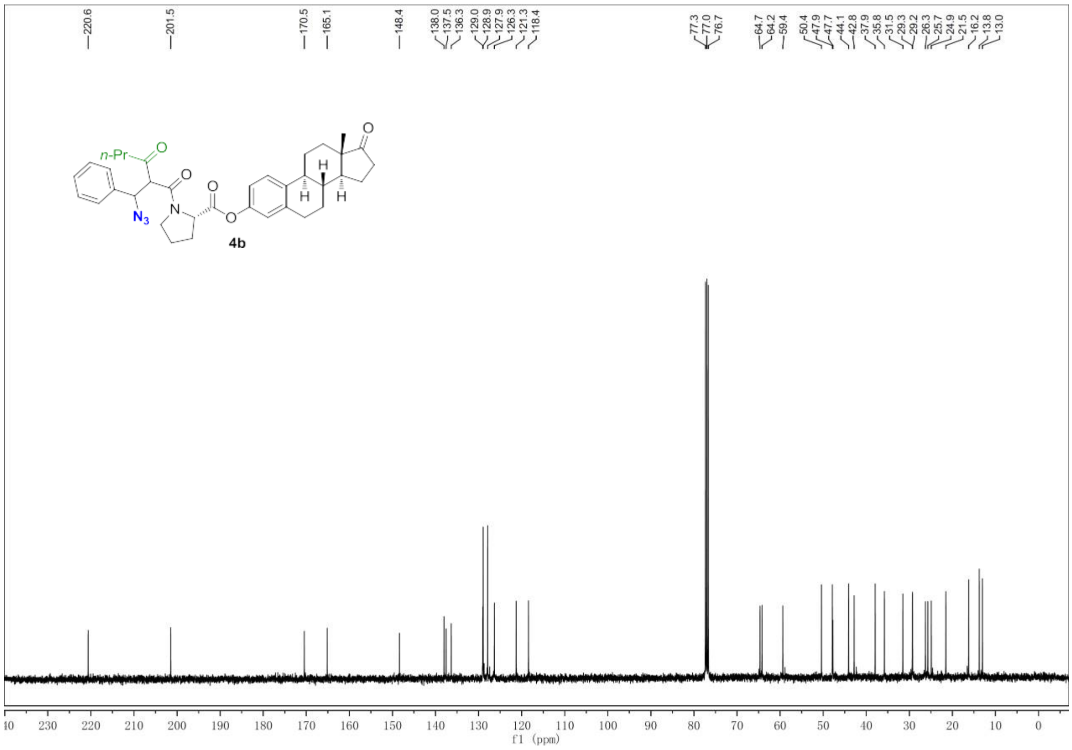

^{1}H NMR spectrum of compound **5a** ($CDCl_3$, 400MHz)

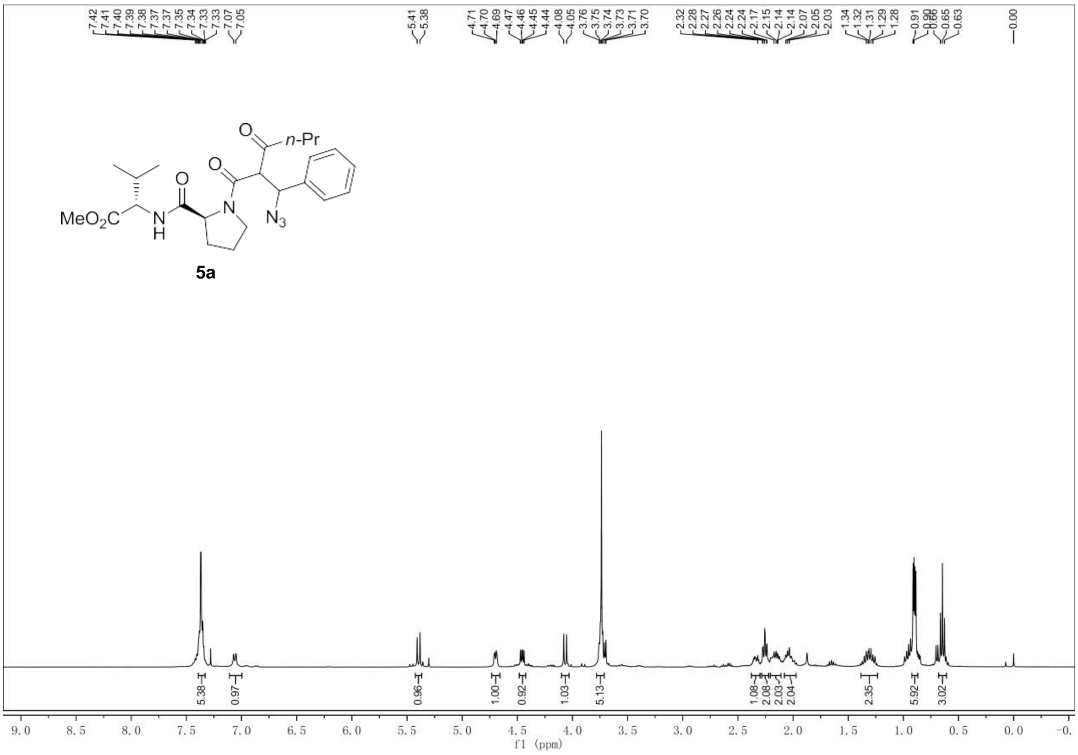

^{13}C NMR spectrum of compound **5a** ($CDCl_3$, 100MHz)

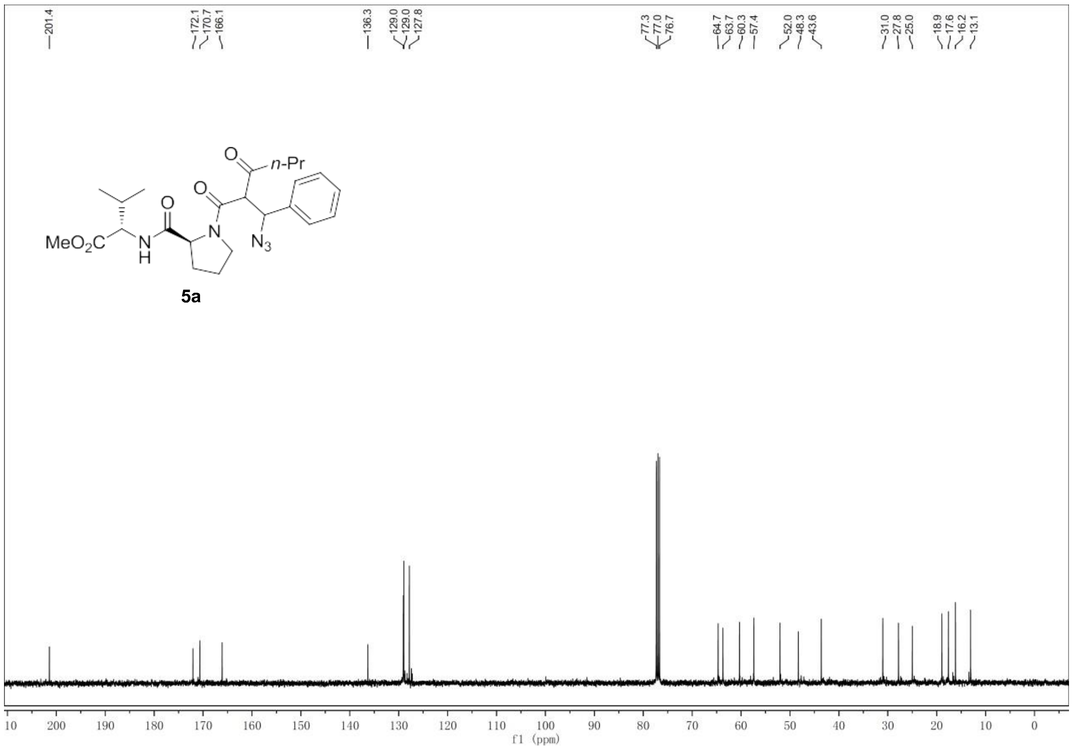

^{1}H NMR spectrum of compound **5b** ($CDCl_3$, 400MHz)

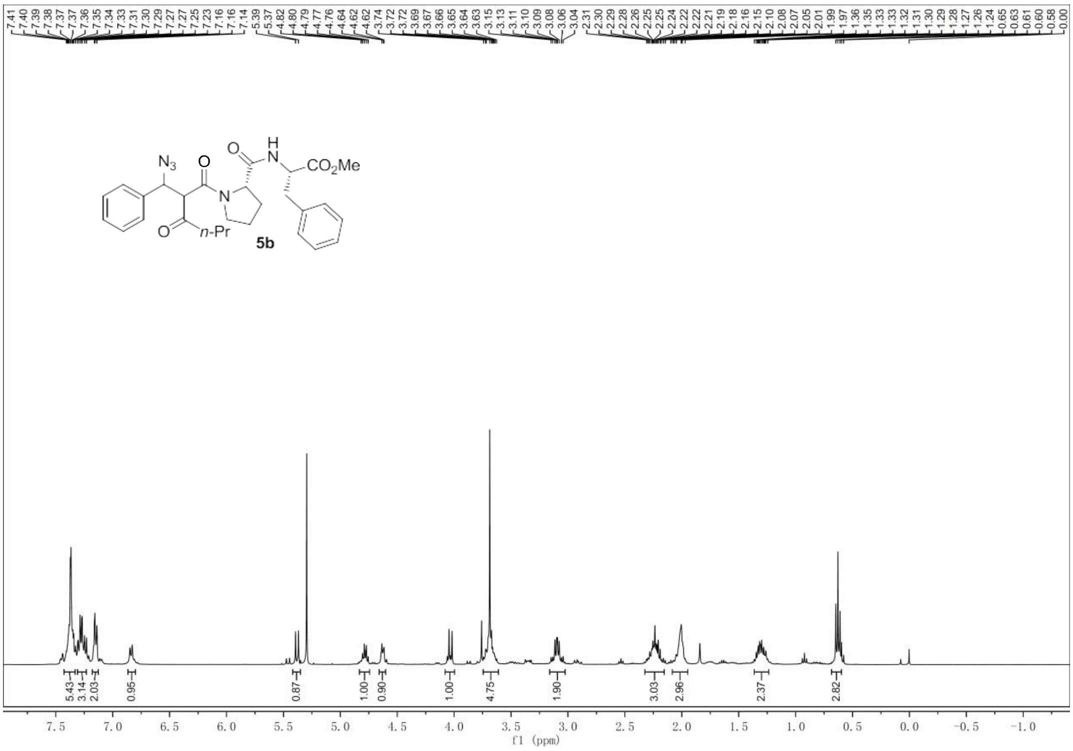

^{13}C NMR spectrum of compound **5b** ($CDCl_3$, 100MHz)

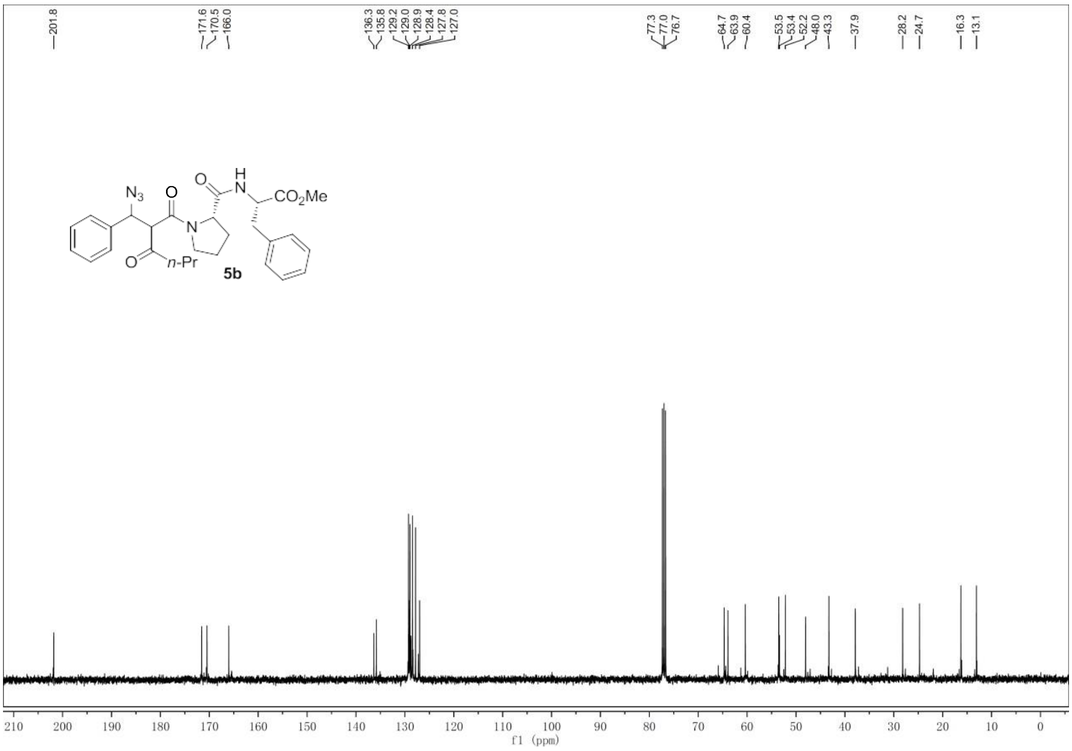

^1H NMR spectrum of compound **9** ($CDCl_3$, 400MHz)

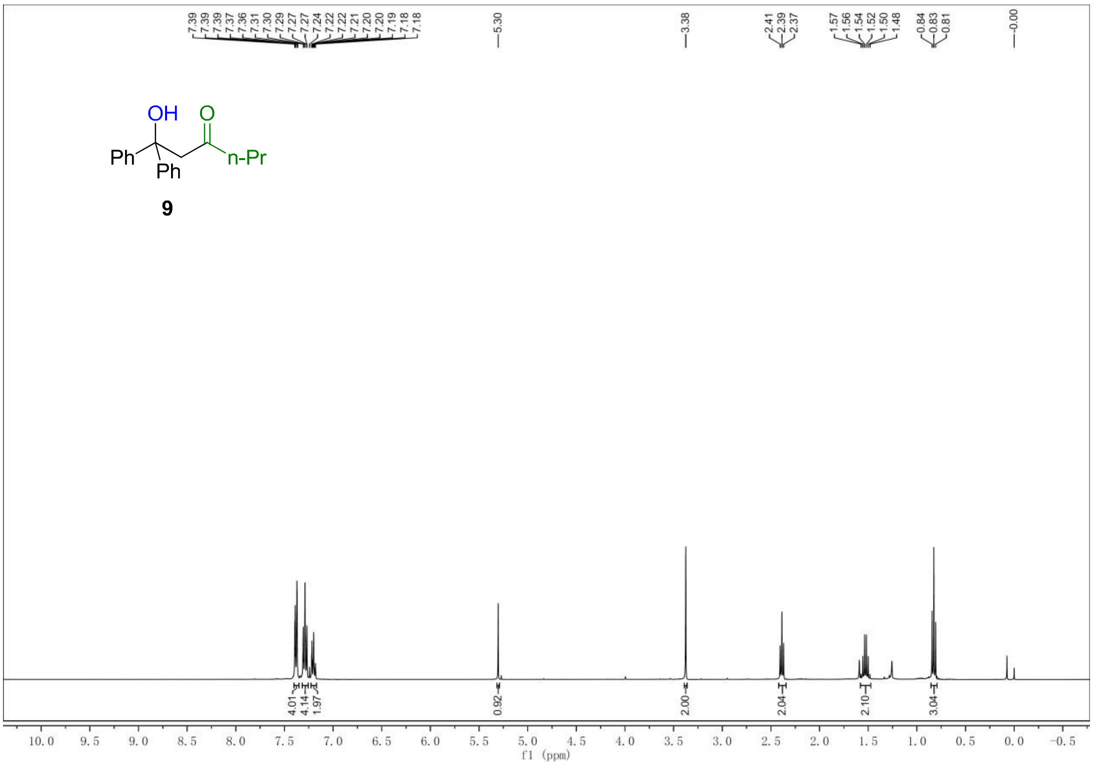

^{13}C NMR spectrum of compound **9** ($CDCl_3$, 100MHz)

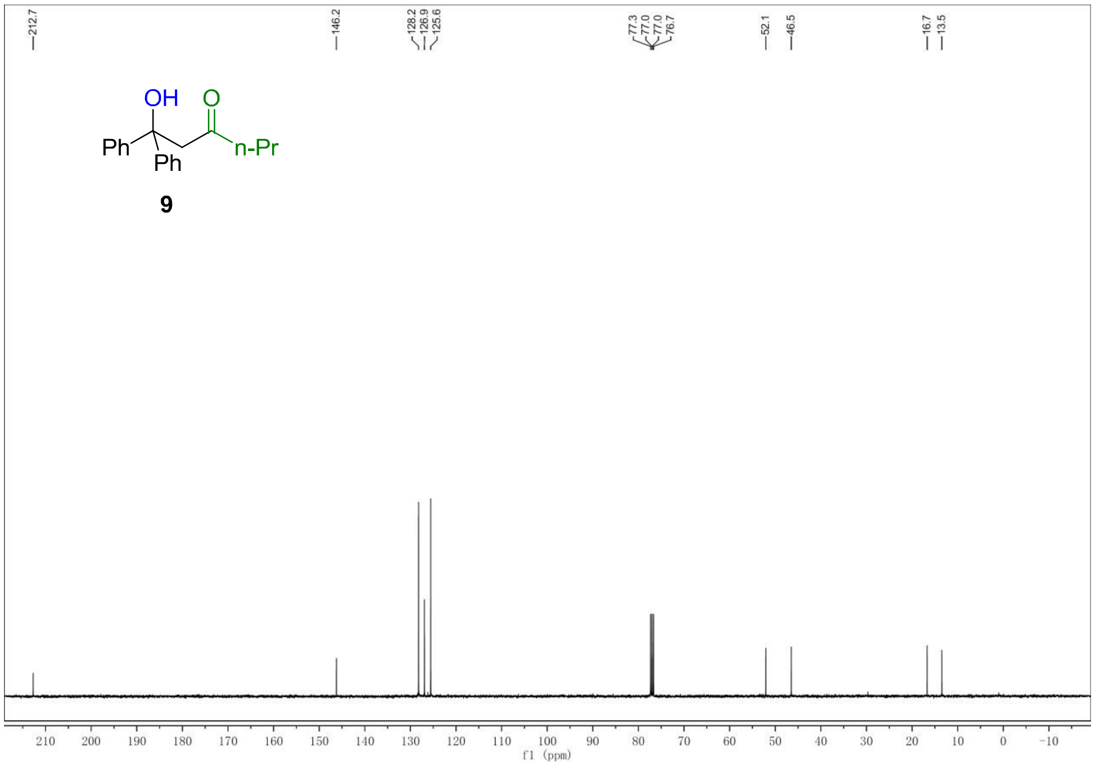

HPLC trace of **3a**

HPLC spectrum of racemic compound **3a:** IC column, hexane: *i*-PrOH = 99:1, 0.5 mL/min;

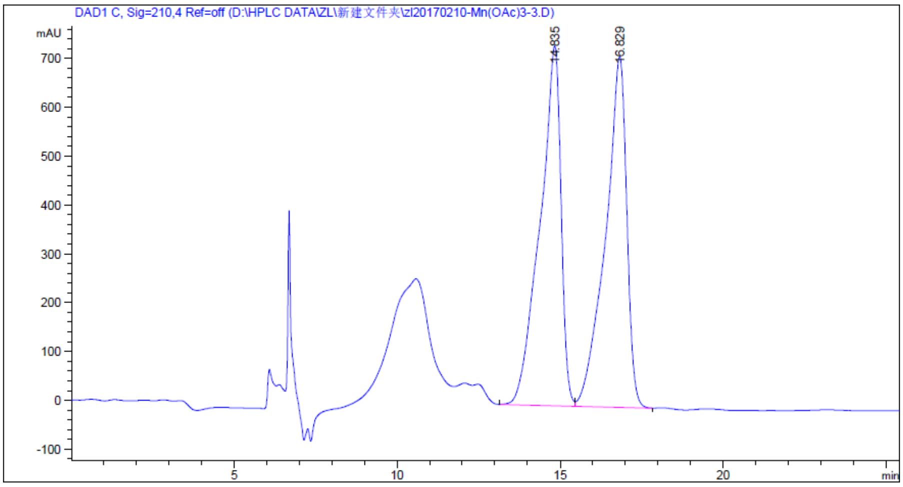

峰 #	保留时间 [min]	类型	峰宽 [min]	峰面积 [mAU*s]	峰高 [mAU]	峰面积 %
1	14.835	BV	0.6256	3.29110e4	736.17560	50.2739
2	16.829	VB	0.6449	3.25525e4	718.07111	49.7261

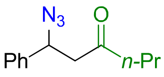

3a

总量 : 6.54635e4 1454.24670

HPLC spectrum of chiral compound **3a,** minor enantiomer *t* = 16.3 min, major enantiomer *t* = 14.4 min.)

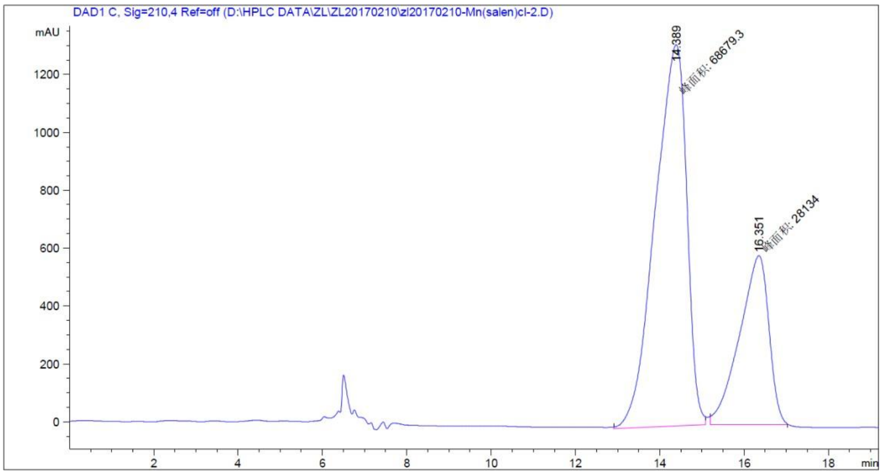

峰 #	保留时间 [min]	类型	峰宽 [min]	峰面积 [mAU*s]	峰高 [mAU]	峰面积 %
1	14.389	MM	0.8696	6.86793e4	1316.23926	70.9399
2	16.351	MM	0.8041	2.81340e4	583.10663	29.0601

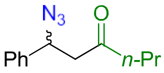

3a

总量 : 9.68133e4 1899.34589

HPLC spectrum of racemic compound **3d** (IC column, hexane: i-PrOH = 99:1, 0.5 mL/min)

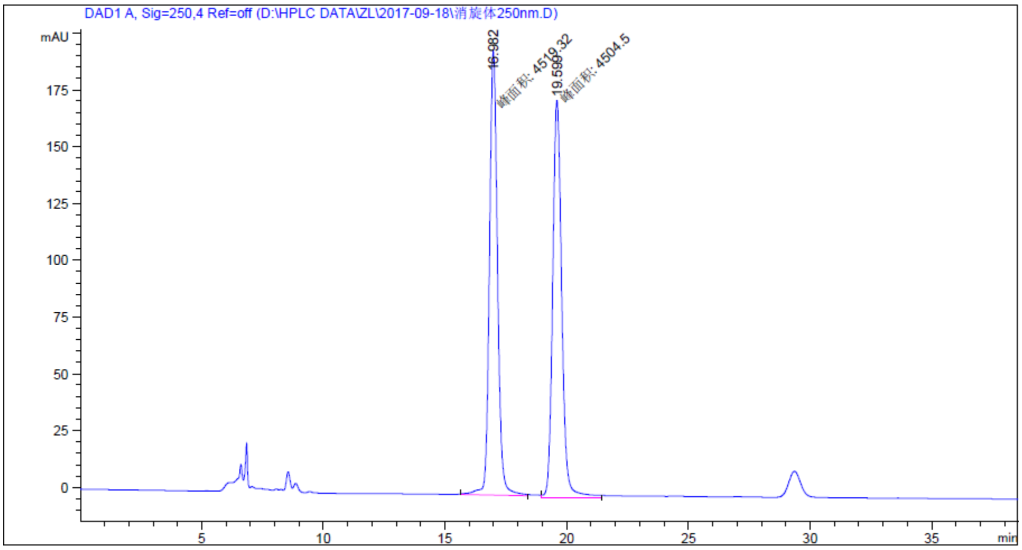

峰 #	保留时间 [min]	类型	峰宽 [min]	峰面积 [mAU*s]	峰高 [mAU]	峰面积 %
1	16.982	MM	0.3857	4519.31592	195.27078	50.0821
2	19.599	MM	0.4298	4504.49902	174.66217	49.9179

总量 : 9023.81494 369.93295

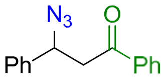

3d

HPLC spectrum of chiral compound **3d** (IC column, hexane: i-PrOH = 99:1, 0.5 mL/min)

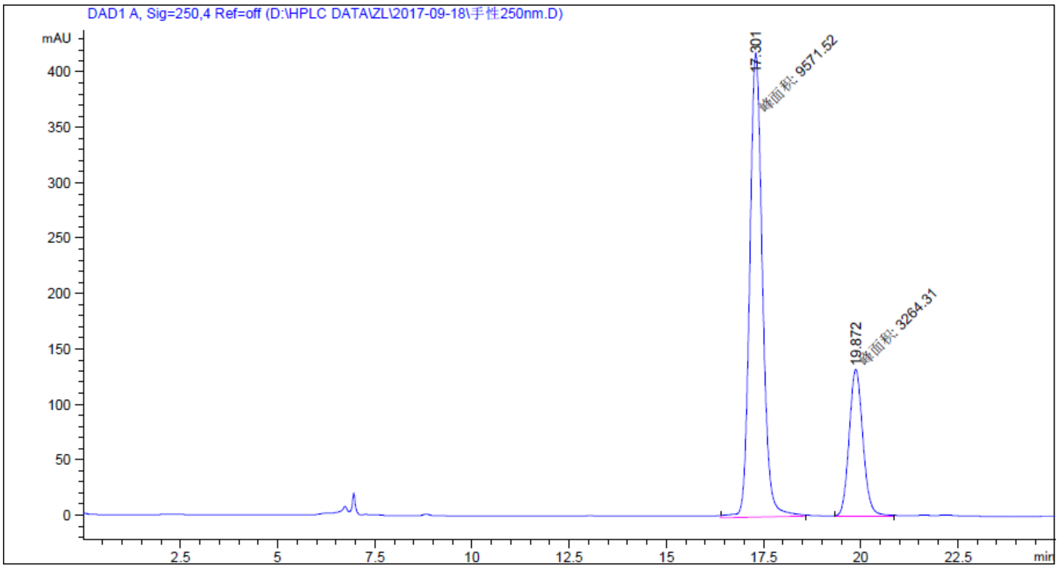

峰 #	保留时间 [min]	类型	峰宽 [min]	峰面积 [mAU*s]	峰高 [mAU]	峰面积 %
1	17.301	MM	0.3807	9571.51660	419.02264	74.5687
2	19.872	MM	0.4105	3264.31226	132.52913	25.4313

总量 : 1.28358e4 551.55177

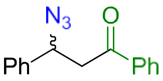

3d

9. References

1. Chatalova-Sazepi, C., Wang, Q., Sammis, G. M., Zhu, J. Copper-Catalyzed Intermolecular Carboetherification of Unactivated Alkenes by Alkyl Nitriles and Alcohols. *Angew. Chem. Int. Ed*. **54**, 5443–5446 (2015).

2. Nimse, S. B., Pal, D., Mazumder, A., Mazumder, R. Synthesis of Cinnamanilide Derivatives and Their Antioxidant and Antimicrobial Activity. *Journal of Chemistry*. **2015**, Article ID 208910, 5 pages.

3. Sanchez-Moreno, C. Review: Methods Used to Evaluate the Free Radical Scavenging Activity in Foods and Biological Systems. *Food Science and Technology International* **8**, 121–137 (2002).

4. Wang, T.-C., Chen, Y.-L., Lee, K.-H., Tzeng, C.-C. Lewis Acid Catalyzed Reaction of Cinnamanilides: Competition of Intramolecular and Intermolecular Friedel-Crafts Reaction. *Synthesis* **1997**, *1*, 87–90 (1997).

5. Furuya, T., Strom, A. E., Ritter, T. Silver-Mediated Fluorination of Functionalized Aryl Stannanes. *J. Am. Chem. Soc*. **131**, 1662–1663 (2009).

6. Straathof, N. J. W., Cramer, S. E., Hessel, V., Noel, T., *Practical Photocatalytic Trifluoromethylation and Hydrotrifluoromethylation of Styrenes in Batch and Flow. Angew. Chem. Int. Ed*. **55**, 15549 –15553 (2016).

7. Liao, S., List, B. Asymmetric Counteranion-Directed Transition-Metal Catalysis: Enantioselective Epoxidation of Alkenes with Manganese(III) Salen Phosphate Complexes. *Angew. Chem. Int. Ed*. **49**, 628–631 (2010).

8. Xi, X., Shao, J., Hu, X., Wu, Y. Structure and asymmetric epoxidation reactivity of chiral Mn(III) salen catalysts modified by different axial anions. *RSC Adv*. **5**, 80772–80778 (2015).

9. Lee, K. N., Lei, Z., Ngai, M.-Y. β -Selective Reductive Coupling of Alkenylpyridines with Aldehydes and Imines via Synergistic Lewis Acid/Photoredox Catalysis. *J. Am. Chem. Soc.* **139**, 5003–5006 (2017).

10. Nandy, J. P., Prabhakaran, E. N., Kumar, S. K., Kunwar, A. C., Iqbal, J. Reverse Turn Induced π-Facial Selectivity during Polyaniline-Supported Cobalt(II) Salen Catalyzed Aerobic Epoxidation of N-Cinnamoyl l-Proline Derived Peptides. *J. Org. Chem.* **68**, 1679–1692 (2003).

11. Zhang, H.-B., Wang, Y., Gu, Y., Xu, P.-F. Lewis- and Brønsted -acid cooperative catalytic radical coupling of aldehydes and azodicarboxylate. *RSC Adv.* **4**, 27796–27799 (2014).
